# Precise Distance Measurements in DNA G‐Quadruplex Dimers and Sandwich Complexes by Pulsed Dipolar EPR Spectroscopy

**DOI:** 10.1002/anie.202008618

**Published:** 2020-11-30

**Authors:** Lukas M. Stratmann, Yury Kutin, Müge Kasanmascheff, Guido H. Clever

**Affiliations:** ^1^ Faculty of Chemistry and Chemical Biology TU Dortmund University Otto-Hahn-Strasse 6 44227 Dortmund Germany

**Keywords:** DNA, G-quadruplexes, metal base-pairing, π-stacking, EPR spectroscopy

## Abstract

DNA G‐quadruplexes show a pronounced tendency to form higher‐order structures, such as π‐stacked dimers and aggregates with aromatic binding partners. Reliable methods for determining the structure of these non‐covalent adducts are scarce. Here, we use artificial square‐planar Cu(pyridine)_4_ complexes, covalently incorporated into tetramolecular G‐quadruplexes, as rigid spin labels for detecting dimeric structures and measuring intermolecular Cu^2+^–Cu^2+^ distances via pulsed dipolar EPR spectroscopy. A series of G‐quadruplex dimers of different spatial dimensions, formed in tail‐to‐tail or head‐to‐head stacking mode, were unambiguously distinguished. Measured distances are in full agreement with results of molecular dynamics simulations. Furthermore, intercalation of two well‐known G‐quadruplex binders, PIPER and telomestatin, into G‐quadruplex dimers resulting in sandwich complexes was investigated, and previously unknown binding modes were discovered. Additionally, we present evidence that free G‐tetrads also intercalate into dimers. Our transition metal labeling approach, combined with pulsed EPR spectroscopy, opens new possibilities for examining structures of non‐covalent DNA aggregates.

## Graphical abstract

Higher‐order G‐quadruplex structures such as dimers and sandwich complexes with G‐quadruplex binders are investigated with pulsed dipolar EPR spectroscopy. Incorporated Cu(pyridine)_4_ complexes allow precise intermolecular Cu^2+^‐Cu^2+^ distance measurements and reveal new binding motifs. High rigidity of the transition metal‐based spin labels results in unprecedentedly narrow peaks in the distance distributions.

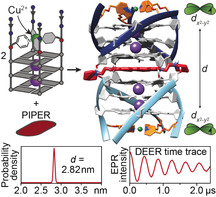

## Introduction

DNA G‐quadruplexes, formed from π‐stacked tetrads of Hoogsteen hydrogen‐bonded guanines, play important roles in several biological processes, including regulation of oncogene expression and maintenance of telomeric repeats upon cell division.[^1^, ^2^, ^3^, ^4^] Beyond their biological role, G‐quadruplexes gained a lot of interest as a structural motif in the field of DNA nanotechnology.[^5^, ^6^, ^7^] G‐quadruplexes tend to form higher‐order structures such as dimers,[^8^, ^9^, ^10^, ^11^] G‐wires,[^12^, ^13^] and other motifs, which are thought to influence their function in vivo.[^14^, ^15^, ^16^, ^17^] It is, for example, not yet understood if and how the multitude of G‐quadruplexes in direct neighborhood interact with each other within the telomeric overhangs.[^17^, ^18^, ^19^, ^20^, ^21^, ^22^]

Due to their regulatory function in pathological processes, G‐quadruplexes have been identified as interesting drug targets in anticancer research.[^23^, ^24^, ^25^] Many small molecules^[26]^ and metal complexes,^[27]^ most of them possessing flat π‐surfaces and positive charges, were found to bind and stabilize the folded secondary structure, thereby acting as potential anticancer drugs.^[28]^ One well‐known G‐quadruplex binder is the natural product telomestatin,[^29^, ^30^] which is a potent telomerase inhibitor due to its strong interaction with unimolecular G‐quadruplexes found in the human telomeric sequence.[^31^, ^32^, ^33^]

Another typical class of G‐quadruplex binders is based on perylene diimides, such as *N*,*N′*‐bis[2‐(1‐piperidino)ethyl]‐3,4,9,10‐perylenetetracarboxylic diimide dihydrochloride (PIPER).[^34^, ^35^, ^36^] An early NMR‐based investigation revealed the formation of a 1:2 sandwich complex of PIPER with tetramolecular G‐quadruplexes, where the dye intercalates between two terminal G‐quartet sites.^[37]^ Such ligand‐mediated or direct non‐covalent contacts between terminal G‐quartets are anticipated to form and play an important role for the overall stability of quadruplexes, and thus their biological function. Therefore, analytical methods that provide key structural information about these π‐stacked aggregates are required.

In the past years, pulsed dipolar electron paramagnetic resonance (PDEPR) methods[^38^, ^39^] have evolved into reliable and versatile tools for structure determination in structural and chemical biology. Pairs of paramagnetic centers based on either organic radicals or open‐shell transition metal complexes are required for distance measurements in the nanometer range, providing valuable information on the structure of biomolecules.^[40]^ The potential of Cu^2+^ ions as spin labels for PDEPR‐based studies has been well documented in the literature over the past years.[^41^, ^42^, ^43^, ^44^, ^45^, ^46^, ^47^, ^48^, ^49^, ^50^, ^51^] While PDEPR is most commonly used to determine structural constraints in protein systems, its application to distance measurements within nucleic acids has been steadily expanding.[^51^, ^52^, ^53^, ^54^, ^55^, ^56^, ^57^, ^58^] So far, few reports describe PDEPR‐based investigations on G‐quadruplexes,[^59^, ^60^, ^61^] including in‐cell spin‐labeled G‐quadruplexes^[62]^ and quadruplex‐metal complex adducts.[^63^, ^64^]

Recently, we incorporated new Cu^2+^‐based spin labels into tetramolecular DNA G‐quadruplexes of varying G‐tetrad count, which allowed us to determine intramolecular distances within the secondary structure with unprecedented accuracy.^[65]^ Key to obtain such precise data was a label design in which the magnetic orbitals of the square‐planar coordinated Cu^2+^ cations were fixed in defined spatial orientations by equipping the four‐stranded DNA construct with a set of four nitrogen donor ligands, forming a rigid chelate environment.

The approach was based on the concept of metal‐mediated base pairing, where canonical nucleotides are replaced by artificial ones, carrying a ligand functionality to coordinate to transition metal cations.[^66^, ^67^] In earlier studies, we transferred this concept from duplex DNA to G‐quadruplexes. Pyridine or imidazole donors were covalently incorporated into defined positions of G‐quadruplex structures, equipping the folded strands with prearranged chelate environments suitable for binding transition metal ions such as Co^2+^, Ni^2+^, Cu^2+^, or Zn^2+^.[^68^, ^69^, ^70^, ^71^, ^72^] Substantial thermal stabilization of the metal ion‐bound structures was observed, and the system allows for metal‐induced control of its folding topology and protein binding behavior.^[73]^

Here, we extend the use of the Cu(pyridine)_4_ tetrad as a rigid spin label to detect intermolecular Cu^2+^–Cu^2+^ distances in G‐quadruplex dimers of different spatial dimensions. This approach also revealed new binding modes in related sandwich complexes with PIPER, telomestatin, and G‐quartets assembled from free guanines. Our study delivers valuable information on binding stoichiometry and key structural parameters, which are in excellent agreement with MD simulation results.

## Results and Discussion

### Synthesis and Characterization of Cu^2+^‐Binding G‐Quadruplexes

**Figure 1:**
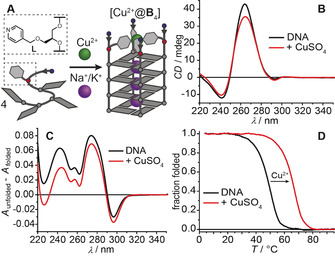
A) Self‐assembly and Cu^2+^ binding of ligand‐modified G‐quadruplex [Cu^2+^@**B**_4_]. The structure of ligandoside **L** is shown. Grey circles and tiles: guanosines; red circles and grey hexagons: ligand modifications; blue circles: thymidines; arrows indicate strand orientations. B) CD spectra, C) UV‐based thermal difference spectra, and D) thermal denaturation profiles of the G‐quadruplex in the absence or presence of Cu^2+^ ions. Sample composition: 16 μm oligonucleotide (4 μm G‐quadruplex), 4 μm CuSO_4_, 100 mm NaCl, 10 mm lithium cacodylate buffer (pH 7.2).

For this study, six short modified oligonucleotides were synthesized by solid‐phase DNA synthesis (oligos **A**–**F**, Table 1). The sequences were chosen based on two known tandem repeat units found in the telomeric regions of different species (TTA GGG and TTG GGG), as these oligonucleotides and related tetramolecular G‐quadruplexes are already well investigated.[^9^, ^11^] Pyridine‐modified nucleotides (**L**, Figure 1 A) were incorporated next to the 5′‐G‐quartets, forming rigid Cu^2+^ spin labels at the 5′‐ends while leaving the 3′‐G‐quartets exposed (oligos **A** and **B**) or blocked by additional thymidines (oligo **C**). Also, isomeric sequences of reverse order, carrying an unobstructed G‐stack at the 5′‐end and the Cu^2+^ modification at the 3′‐termini, were synthesized (oligos **D**–**F**).

**Table 1:** Sequences of ligand‐modified oligonucleotides used in this work.

Name	Sequence (5′→3′)		Name	Sequence (5′→3′)
Oligo **A**	TT**L** GGG		Oligo **D**	GGG **L**TT
Oligo **B**	T**L**G GGG		Oligo **E**	GGG G**L**T
Oligo **C**	TT**L** GGG T		Oligo **F**	TGG G**L**T T

We first examined the formation of G‐quadruplex structures using CD spectroscopy. For all six oligonucleotides, with a high Na^+^ or K^+^ concentration at pH 7.2, formation of a parallel G‐quadruplex topology was indicated in the absence as well as in the presence of Cu^2+^ ions (Figure 1). In addition, UV‐based thermal difference spectra and thermal denaturation profiles confirmed G‐quadruplex formation. For all oligonucleotides, the presence of Cu^2+^ ions caused a significant increase of the thermal denaturation temperatures, as the formation of a Cu(pyridine)_4_ complex was previously shown to raise the overall stability of the hybridized quadruplex structure[^68^, ^69^] (e.g. Δ*T*_1/2_=17 °C for [Cu^2+^@**B**_4_] in NaCl‐containing solution, Figure 1 D; for further CD and UV spectroscopy results see Figures S3–S23). However, we note that the usual set of CD and UV experiments gave no hints on the potential presence of dimeric species.

In the next step, EPR‐based investigations were performed. Samples containing 250 μm G‐quadruplex monomers (1 mm oligonucleotides) and 375 μm CuSO_4_ (1.5 equiv per quadruplex) were prepared in 50 mm potassium phosphate buffer (pH 7.0), then mixed with glycerol (1:1 v/v) and immediately frozen in liquid N_2_. The final G‐quadruplex monomer concentration was 125 μm for all EPR samples investigated in this work.

**Figure 2:**
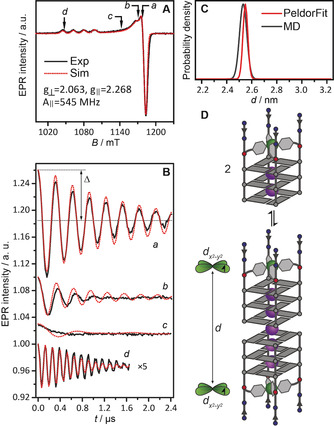
A) Derivative pulsed EPR spectrum of dimer [Cu^2+^@**A**_4_]_2_ (black line) recorded at 34 GHz and 19 K with its corresponding simulation (red line). B) Background‐corrected orientation‐selective DEER time traces measured at four field positions (black lines), overlaid with the best fit results obtained from PeldorFit (red lines). Observer positions are marked with *a*–*d* in panel (A) and correspond to *g*_eff_=2.061, 2.071, 2.121, and 2.315, respectively. The modulation depth parameter Δ is shown in trace *a*; Δ of trace *d* has been increased for clarity. C) Distance distribution obtained from the experiment using PeldorFit (red line) and the MD simulation result (gray line). D) Equilibrium between monomeric G‐quadruplex [Cu^2+^@**A**_4_] and its dimeric species [Cu^2+^@**A**_4_]_2_, formed through tail‐to‐tail stacking of the 3′‐terminal G‐tetrads. The distance between the *d* ${}_{x{}^{2}-y{}^{2}}$ orbitals of the Cu^2+^ ions containing the unpaired electrons in the dimer is indicated.

A typical field‐swept EPR spectrum of the Cu^2+^ spin label fixed in its spatial position by the four pyridine ligands is shown in Figure 2 A (for [Cu^2+^@**A**_4_]). The best fit was obtained using the following spin‐Hamiltonian parameters: g_∥_=2.268, g_⊥_=2.063, A_∥_=545 MHz. These values are in good agreement with parameters for a Cu^2+^ ion coordinated by four nitrogen atoms.[^74^, ^75^, ^76^] We note that the choice of the modified oligonucleotide resulted in slight variations in the EPR line shape of the spin label (Figure S25). In agreement with previous studies,[^51^, ^77^] unbound Cu^2+^ ions in solution were nearly EPR‐silent at pH ≥7 and provided only a negligible contribution to the overall EPR line shape (Figure S26).

### Orientation‐Selective PDEPR Reveals G‐Quadruplex Dimers

In each G‐quadruplex, one terminal site was modified to carry the metal complex, while the other end remained unmodified to allow stacking of the terminal G‐tetrads as observed in unmodified sequences.[^8^, ^9^, ^11^] PDEPR experiments were performed to investigate dimer formation by detecting dipole–dipole interactions, and thus measuring distances between the two paramagnetic Cu^2+^ ions residing in the π‐stacked G‐quadruplex monomers. Both double electron–electron resonance (DEER, also known as PELDOR)[^78^, ^79^] and relaxation‐induced dipolar modulation enhancement (RIDME)[^80^, ^81^] techniques were employed, the former providing more robust background correction and the latter resulting in larger modulation depths (Δ) and being generally less prone to orientation selectivity.[^40^, ^50^]

For rigid, orientationally correlated spin pairs, orientation selectivity in PDEPR leads to a deviation of dipolar spectra from a Pake pattern and to the dependence of the dipolar frequency on the selected *g*‐tensor orientations.^[82]^ Therefore, these experiments provide atomic‐level structural information on the geometry of the spin system. Some examples include tyrosyl radicals in ribonucleotide reductase,^[83]^ spin‐labeled DNA,^[57]^ Co^2+^‐ and Fe^3+^‐containing synthetic systems.[^84^, ^85^]

Since the Cu^2+^ spin label within a G‐quadruplex monomer is fixed in a highly rigid fashion,^[65]^ and the total width of a Cu^2+^ EPR spectrum (148 mT, about 4 GHz) is significantly larger than a typical pulse bandwidth (ca. 50 MHz), orientation selectivity is expected to affect PDEPR dipolar spectra. Indeed, both DEER and RIDME data acquired at different field positions for quadruplexes composed of oligos **A**, **B**, **D** and **E** showed strong dependence of the dipolar frequency on the excited *g*‐tensor orientation, displaying the rigidity of the systems (Figures 2 B and S28–S33). While there was a clear difference between RIDME and DEER modulation depths, the two methods produced almost identical dipolar spectra, particularly at orientations that correspond to the distance vector perpendicular and parallel to the magnetic field vector (Figure S29). In the text below, we focus on the analysis of DEER‐derived Cu^2+^–Cu^2+^ distances.

PeldorFit,^[86]^ which explicitly takes orientation selectivity into account when deriving distances, was used to analyze the PDEPR data. It allowed us to reproduce DEER time traces including both perpendicular and parallel orientations of the dipolar coupling tensor with one set of parameters (Figure 2 B). Importantly, this analysis showed that *g_z_* axes of the two Cu^2+^ spin labels within a G‐quadruplex dimer are aligned collinearly, perfectly fitting the expected structure for two rigid coplanar Cu^2+^ complexes and a tight π‐stacking interface between the monomers (see Figures S34–S35 and Table S2 for details).

Analysis of DEER data from the [Cu^2+^@**A**_4_]_2_ sample revealed a single Cu^2+^–Cu^2+^ mean distance of *d*_A_=2.55 nm with a very narrow distance distribution of *σ*=0.02 nm (*σ* is the standard deviation of the distribution, which is assumed Gaussian, Figures 2 and S30). The detected distance was in the expected range for a G‐quadruplex dimer formed through tail‐to‐tail stacking of the 3′‐terminal G‐tetrads. For a sample of dimer [Cu^2+^@**B**_4_]_2_, a larger mean distance of *d*_B_=3.21 nm (*σ*=0.02 nm, Figure S31) was obtained, which was about two π‐stacking distances longer than that of the shorter [Cu^2+^@**A**_4_]_2_ dimer (*d*_B_−*d*_A_=0.66 nm). Since each [Cu^2+^@**B**_4_] monomer contains one additional G‐tetrad, this result is in perfect agreement with the expected distance.

Next, isomeric G‐quadruplex dimers with inverted sequences, [Cu^2+^@**D**_4_]_2_ and [Cu^2+^@**E**_4_]_2_, were investigated, with the Cu(pyridine)_4_ complex located at the 3′‐end and the exposed terminal G‐tetrad at the 5′‐end. In this case, the hyperfine structure of the Cu^2+^ EPR spectrum was less pronounced, indicating a somewhat different magnetic environment at the 3′‐end (Figure S25). Additionally, DEER‐derived distances of *d*_D_=2.48 nm (*σ*=0.04 nm, Figure S32) for [Cu^2+^@**D**_4_]_2_ and *d*_E_=3.17 nm (*σ*=0.04 nm, Figure S33) for [Cu^2+^@**E**_4_]_2_ were slightly shorter than those of their respective isomers (*d*_A_−*d*_D_=0.07 nm and *d*_B_−*d*_E_=0.04 nm), most probably caused by small structural differences between the Cu(pyridine)_4_ complexes embedded at the 5′‐ or 3′‐end.

As controls, G‐quadruplexes [Cu^2+^@**C**_4_] and [Cu^2+^@**F**_4_], both carrying obstructing thymidines next to the terminal G‐quartets, were probed. DEER time traces showed no dipolar modulation of the Cu^2+^ EPR signal (Figure S36), indicating that no dimers were present in these samples. This result confirms that extra 3′‐ or 5′‐terminal thymidines prevent G‐quadruplex aggregation in solution.[^8^, ^9^, ^11^]

Our PDEPR experiments demonstrate that a variety of Cu^2+^‐binding G‐quadruplex monomers of different lengths, carrying an exposed terminal G‐tetrad at either 3′‐ or 5′‐end, readily assemble dimers. Mean Cu^2+^–Cu^2+^ distances and corresponding distributions for all dimers are listed in Table 2. The distance distributions achieved in this work were approximately 5 to 10 times narrower than those obtained for DNA and RNA structures labeled either with nitroxide‐[^53^, ^55^, ^57^, ^58^, ^59^] or other, less structurally confined Cu^2+^‐based[^51^, ^63^] spin labels. Such narrow distance distributions not only highlight the pronounced rigidity of our Cu(pyridine)_4_ spin label within its G‐quadruplex environment, but also demonstrate the overall defined structure adopted by the G‐quadruplex dimers investigated in this work.

**Table 2:** Comparison of Cu^2+^–Cu^2+^ distances obtained from DEER experiments and MD simulations in G‐quadruplex dimers and related sandwich complexes.

G‐quadruplex adduct	⟨*d*⟩ [nm], PeldorFit^[a]^	⟨*d*⟩ [nm], MD^[b]^
[Cu^2+^@**A**_4_]_2_	2.55 (0.02)	2.53 (0.03)
[Cu^2+^@**B**_4_]_2_	3.21 (0.02)	3.23 (0.03)
[Cu^2+^@**C**_4_]	No dimerization^[c]^	–
[Cu^2+^@**D**_4_]_2_	2.48 (0.04)	2.55 (0.04)
[Cu^2+^@**E**_4_]_2_	3.17 (0.04)	3.34 (0.04)
[Cu^2+^@**F**_4_]	No dimerization^[c]^	–
PIPER@[Cu^2+^@**A**_4_]_2_	2.82 (0.03)	2.84 (0.03)
PIPER@[Cu^2+^@**B**_4_]_2_	3.48 (0.05)	3.46 (0.05)
2PIPER@[Cu^2+^@**A**_4_]_2_	3.21 (0.05)	3.18 (0.03)
telomestatin@[Cu^2+^@**A**_4_]_2_	2.88 (0.04)	2.88 (0.04)
guanine_4_@[Cu^2+^@**A**_4_]_2_	2.88 (0.03)	–^[d]^
guanine_4_@[Cu^2+^@**B**_4_]_2_	3.54 (0.03)	–^[d]^
guanosine_4_@[Cu^2+^@**A**_4_]_2_	2.88 (0.02)	2.88 (0.03)
guanosine_4_@[Cu^2+^@**B**_4_]_2_	3.54 (0.01)	3.56 (0.03)

[a] Mean values with standard deviations in parenthesis of PeldorFit‐derived distance distributions, which were assumed Gaussian (see SI 5.6). [b] Mean values with standard deviations of MD‐derived distance distributions. [c] Additional thymidines prevent dimerization by blocking the terminal G‐tetrad. [d] Not determined.

### Sandwich Complexes Based on G‐Quadruplex Dimers

The ability to easily distinguish between dimeric species of different lengths with high resolution and accuracy prompted us to employ DEER to investigate intercalation of two well‐known quadruplex binders.

Upon addition of 0.5 equiv of PIPER per G‐quadruplex, CD and UV/Vis spectroscopy again confirmed formation of a parallel G‐quadruplex topology for all samples in the absence and presence of Cu^2+^ ions. However, the addition of PIPER increased the thermal stability of the secondary structures (Figure 3). Furthermore, in the absence of G‐quadruplex DNA, PIPER was not soluble at pH 7.2 and precipitated (Figure S1). In the presence of folded G‐quadruplex DNA, red‐colored PIPER stayed in solution, giving rise to a distinct absorbance signature at 450–600 nm (Figure 3). After thermal denaturation of the secondary structure, PIPER precipitated, and the absorbance signal in the visible region vanished. This observation supported a selective interaction of PIPER with G‐quadruplex DNA as compared to single‐stranded DNA^[87]^ (see Figures S3–S24 for further CD and UV/Vis spectroscopy results).

**Figure 3:**
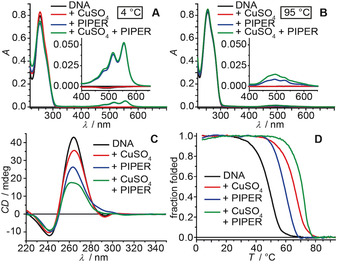
A) UV/Vis spectra at 4 °C and B) at 95 °C, C) CD spectra, and D) thermal denaturation profiles of G‐quadruplexes composed of oligo **B** in the absence or presence of Cu^2+^ ions and/or PIPER. Sample composition: 16 μm oligonucleotide (4 μm G‐quadruplex), 4 μm CuSO_4_, 2 μm PIPER, 100 mm NaCl, 10 mm lithium cacodylate buffer (pH 7.2).

**Figure 4:**
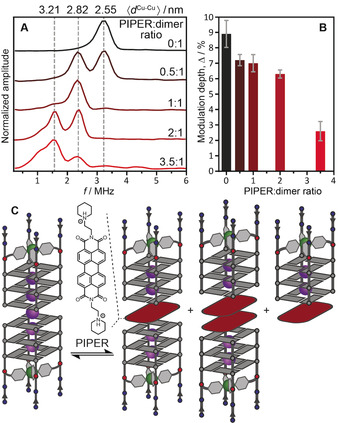
A) DEER dipolar spectra (*g*_eff_=2.061) and B) modulation depths (mean values and standard deviations based on three samples per point) of [Cu^2+^@**A**_4_]‐containing samples with varying PIPER‐to‐G‐quadruplex dimer ratios. Corresponding mean Cu^2+^–Cu^2+^ distances are shown on the top axis of panel (A). C) Equilibrium between [Cu^2+^@**A**_4_]_2_ and its different complexes with the PIPER dye (PIPER@[Cu^2+^@**A**_4_]_2_, 2PIPER@[Cu^2+^@**A**_4_]_2_, PIPER@[Cu^2+^@**A**_4_]). G‐quadruplex monomer concentration was 125 μm in all samples.

EPR samples containing [Cu^2+^@**A**_4_]_2_ dimers and stoichiometric PIPER concentration showed a new, lower modulation frequency in the DEER data and revealed a Cu^2+^–Cu^2+^ distance of *d*_P_=2.82 nm, larger than that of pure [Cu^2+^@**A**_4_]_2_ dimers (*d*_P_−*d*_A_=0.27 nm, Figures S37–S38 and Figure 4 A). A similar result was obtained with samples containing PIPER and [Cu^2+^@**B**_4_]_2_ dimers (Figure S39). The increase in the spatial separation between the two Cu^2+^ ions demonstrated the formation of sandwich complexes (PIPER@[Cu^2+^@**A**_4_]_2_ and PIPER@[Cu^2+^@**B**_4_]_2_), in which the flat organic molecule intercalates between the 3′‐faces of the two G‐quadruplex monomers.^[37]^ However, the addition of PIPER to both [Cu^2+^@**D**_4_]_2_ and [Cu^2+^@**E**_4_]_2_ dimers did not affect the obtained distance distributions, demonstrating that PIPER does not intercalate into 5′‐5′‐stacked dimers (Figure S41), but prefers a different binding mode. This result is in agreement with the observed differences in CD signals of PIPER induced by the 3′‐ and 5′‐modified quadruplexes (Figure S24).

Next, we investigated the effect of PIPER concentration on the sandwich complex formation. At a PIPER‐to‐[Cu^2+^@**A**_4_]_2_ ratio of 0.5:1, the peak intensities corresponding to pure dimer (*d*_A_=2.55 nm) and PIPER@[Cu^2+^@**A**_4_]_2_ complex (*d*_P_=2.82 nm) in the dipolar spectrum were equivalent (Figure 4 A), revealing approximately equal concentrations of pure and PIPER‐containing dimers. Once the PIPER‐to‐dimer ratio was raised to 1:1, the frequency component of the pure dimer disappeared and only the distance of *d*_P_=2.82 nm was detected, indicating a high binding constant for PIPER to the [Cu^2+^@**A**_4_]_2_ dimer. These results suggest a monomer–dimer equilibrium far on the side of the dimer, in agreement with literature data for (TTAGGG)_4_ at high K^+^ concentrations.^[9]^ The same conclusion may be reached based on the DEER modulation depth parameter Δ, which reflects the number of dimers present in the sample.^[88]^ In our case, since Δ obtained in the g_⊥_ region is not affected by the excess Cu^2+^ (Figure S27), it provides information on the dimerization efficiency of G‐quadruplexes. Upon addition of one PIPER molecule per dimer, Δ did not increase, suggesting that the whole G‐quadruplex population was already present in the dimeric form prior to the PIPER addition.

Surprisingly, further increase in the PIPER‐to‐dimer ratio led to the appearance of a new Cu^2+^–Cu^2+^ distance of *d*_2P_=3.21 nm, about one π‐stacking distance longer than that of the PIPER@[Cu^2+^@**A**_4_]_2_ complex (*d*_2P_−*d*_P_=0.39 nm). This distance was assigned to a species where two PIPER ligands intercalate between the monomers of a tail‐to‐tail arranged G‐quadruplex dimer (2PIPER@[Cu^2+^@**A**_4_]_2_, Figures 4 C and S40). To the best of our knowledge, this binding mode of PIPER to DNA G‐quadruplexes has never been described before. It resembles a reported motif found in the solid state, where two naphthalene diimide derivatives intercalate into a head‐to‐head arranged dimer of unimolecular G‐quadruplexes.^[89]^ The modulation depth strongly decreased with an increase in the PIPER‐to‐dimer ratio beyond 2:1 (Figures 4 B and S37). This suggests a disruption of the 2PIPER@[Cu^2+^@**A**_4_]_2_ complex and the formation of a monomeric species, presumably PIPER@[Cu^2+^@**A**_4_], which contains only one Cu^2+^ center, and thus cannot be detected with PDEPR. There appears to be a marginal decrease in Δ upon PIPER addition up to 2:1 PIPER‐to‐dimer ratio. Same has been observed for 5′‐5′‐stacked dimers, in which PIPER does not intercalate (Figure S41). These data suggest that up to two equivalents PIPER addition reduces the number of dimers by forming a small amount of monomeric adducts with quadruplexes.

EPR‐based distance measurements were also performed in the presence of telomestatin,[^29^, ^30^] another well‐known G‐quadruplex binder. The natural product was described to bind to unimolecular G‐quadruplexes, interacting with terminal G‐quartets via π‐stacking.[^31^, ^90^] Interestingly, samples of the [Cu^2+^@**A**_4_]_2_ dimer containing telomestatin (1 equiv per dimer, Figure S42) revealed a second peak in the distance distribution with an increased Cu^2+^–Cu^2+^ distance of *d*_T_=2.88 nm, with respect to that of the pure dimer (*d*_T_−*d*_A_=0.33 nm). This observation strongly suggests that telomestatin can indeed intercalate into a 3′‐3′ π‐stacked dimer formed from two tetramolecular [Cu^2+^@**A**_4_] monomers with parallel topology to build a telomestatin@[Cu^2+^@**A**_4_]_2_ sandwich complex. To the best of our knowledge, this is the first time such a sandwich binding mode of telomestatin has been observed. Since human telomeric sequences are able to form (unimolecular) parallel G‐quadruplexes with exposed terminal G‐tetrads,[^18^, ^22^] the discovered binding mode might play a role in the ability of telomestatin to inhibit telomerase.

### Intercalation of Free G‐Quartets into G‐Quadruplex Dimers

**Figure 5:**
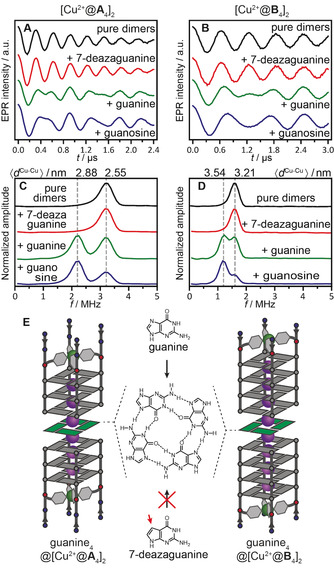
A,B) Background‐corrected DEER time traces (*g*_eff_=2.061) for [Cu^2+^@**A**_4_]_2_ and [Cu^2+^@**B**_4_]_2_, respectively, in the absence and presence of 7‐deazaguanine, guanine or guanosine (4 equiv per dimer). C,D) Corresponding dipolar spectra, with mean Cu^2+^–Cu^2+^ distances shown on the top axis. E) Sandwich complexes guanine_4_@[Cu^2+^@**A**_4_]_2_ and guanine_4_@[Cu^2+^@**B**_4_]_2_ with a free G‐tetrad intercalating between the two G‐quadruplex monomers.

Moreover, we investigated the interaction of derivatives of free guanine with G‐quadruplex dimers. First, we added guanine (4 equiv per dimer) to samples of [Cu^2+^@**A**_4_]_2_ and [Cu^2+^@**B**_4_]_2_. For both dimers, this resulted in the appearance of a second peak in the distance distribution, accounting for an increase in length of Δ*d*=0.33 nm as compared to the pure dimers (Figures S43–S44). The concentration ratio of the new extended dimeric species to the original one was approximately 1:1 for both samples (Figure 5, green traces). The new distance can be explained by a species where a whole untethered G‐tetrad assembled from four free guanines intercalates between the two quadruplex monomers (guanine_4_@[Cu^2+^@**A**_4_]_2_ and guanine_4_@[Cu^2+^@**B**_4_]_2_, Figure 5 E and Table 2).

Next, the experiments were repeated with 7‐deazaguanine (Figure 5, red traces), guanosine (blue traces), and guanosine monophosphate (GMP) under the same conditions (Figures S45–S47). 7‐Deazaguanine lacks a nitrogen atom necessary for Hoogsteen hydrogen bonding, and thus is unable to form tetrads.^[91]^ As anticipated, its addition did not alter the distance distributions of the pure dimers, indicating that not a single nucleobase, but a quartet acts as intercalating species. The addition of GMP did not alter the distance distribution either, since tetrads formed from GMP are highly negatively charged at pH 7, and thus suffer from electrostatic repulsion. In contrast, guanosine (as free guanosine quartet) was found to intercalate even more efficiently than a guanine tetrad, as judged by the relative intensities in the dipolar spectra, presumably due to its higher solubility in water. Thus, all additional results support the hypothesis of an untethered G‐quartet assembled from free guanines intercalating into the G‐quadruplex dimers.

### MD Simulation of Dimeric G‐Quadruplex Structures and Sandwich Complexes

**Figure 6:**
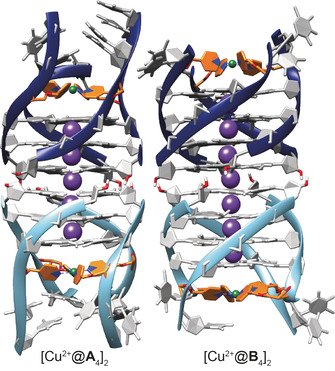
Structural models derived from MD simulations of G‐quadruplex dimers [Cu^2+^@**A**_4_]_2_ and [Cu^2+^@**B**_4_]_2_. Phosphate backbone: dark or light blue ribbon; K^+^ and Cu^2+^ ions: violet and green spheres, respectively; pyridine ligand modification: orange.

In order to relate experimentally determined distances to structural models, MD simulations were performed for each dimeric system. Starting structures were created assuming a tail‐to‐tail or head‐to‐head stacking of the terminal G‐tetrads, and 50 ns MD runs in explicit TIP3P water with 100 mm KCl concentration were conducted (for more details, see the Supporting Information). The dimeric structures were preserved throughout the simulation time (Figures 6 and S48–S51) and the obtained intermolecular Cu^2+^–Cu^2+^ distances and distance distributions matched the experimental values very well (Table 2).

**Figure 7:**
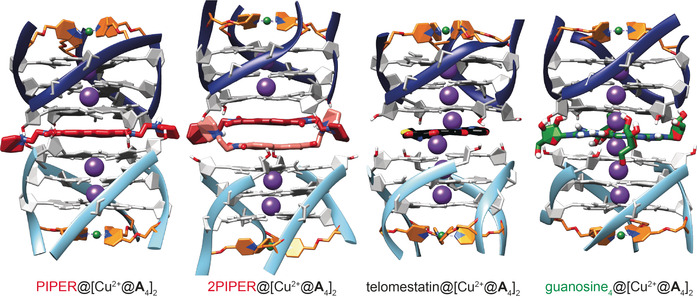
MD‐derived structural models of sandwich complexes PIPER@[Cu^2+^@**A**_4_]_2_, 2PIPER@[Cu^2+^@**A**_4_]_2_, telomestatin@[Cu^2+^@**A**_4_]_2_, and guanosine_4_@[Cu^2+^@**A**_4_]_2_. PIPER is highlighted in red, telomestatin in black, and the free guanosine tetrad in green. Thymidine nucleotides are omitted for clarity (for complete structural models see Figures S52–S57).

Sandwich adducts with PIPER, telomestatin or free guanosine quartets as intercalating species were also simulated. Starting structures were created with typical π‐stacking distances between the respective intercalator and the G‐quadruplex monomers. Again, the sandwich structures were preserved during the whole MD run, and the Cu^2+^–Cu^2+^ distances agreed extremely well with DEER‐derived ones (Table 2, Figures 7 and S52–S58). In the special case of adduct 2PIPER@[Cu^2+^@**A**_4_]_2_, the simulation gave information on the relative orientation of the two PIPER molecules with respect to each other. Throughout the MD run, the relative rotation angle was quite flexible at around 40–60° (Figure S57).

The overall agreement between experimentally obtained and MD‐derived distances confirmed that PDEPR spectroscopy allows measuring intermolecular Cu^2+^–Cu^2+^ distances in π‐stacked G‐quadruplex dimers and related sandwich complexes with high accuracy.

## Conclusion

We have shown that paramagnetic Cu(pyridine)_4_ spin labels incorporated into DNA G‐quadruplexes are suitable for the investigation of higher‐order G‐quadruplex structures such as dimers, by means of intermolecular Cu^2+^–Cu^2+^ distance measurements using PDEPR techniques. Due to the unprecedented rigidity of the spin label and the concomitant sharp peaks in the distance distributions, the method provides a simple readout for the unambiguous characterization of dimers of different lengths and composition. Moreover, intercalation of G‐quadruplex‐binding ligands such as PIPER and telomestatin was clearly demonstrated with the new method, revealing previously undescribed binding modes.

Surprisingly, we were also able to show that untethered G‐quartets, composed of free guanines or guanosines, intercalate into G‐quadruplex dimers. This observation may be relevant for applications exploiting the supramolecular interaction of functionalized G‐quartet probes with G‐quadruplexes of biological origin.^[92]^

Furthermore, the introduced methodology herein, based on an easy to synthesize DNA modification and an established EPR protocol, showcases the possibility to investigate more complex G‐quadruplex systems, other higher‐order oligonucleotide architectures, as well as DNA–protein interactions. We expect this method to provide valuable contributions to the investigation of structure and dynamics in both biological and DNA‐nanotechnological contexts.

## Conflict of interest

The authors declare no conflict of interest.

## Supporting information

As a service to our authors and readers, this journal provides supporting information supplied by the authors. Such materials are peer reviewed and may be re‐organized for online delivery, but are not copy‐edited or typeset. Technical support issues arising from supporting information (other than missing files) should be addressed to the authors.

Supplementary
