# Supplementary material for: Precise Distance Measurements in DNA G‐Quadruplex Dimers and Sandwich Complexes by Pulsed Dipolar EPR Spectroscopy
Source: Angew Chem Int Ed Engl. 2020 Nov 30;60(9):4939–47. doi: 10.1002/anie.202008618 (PMC7984025; doi:10.1002/anie.202008618)
Supplement: Supplementary file 1 — Supplementary [file ANIE-60-4939-s001.pdf]

Angewandte Chemie
Eine Zeitschrift der Gesellschaft Deutscher Chemiker

Supporting Information

Precise Distance Measurements in DNA G-Quadruplex Dimers and Sandwich Complexes by Pulsed Dipolar EPR Spectroscopy

Lukas M. Stratmann, Yury Kutin, Müge Kasanmascheff, and Guido H. Clever**

anie_202008618_sm_miscellaneous_information.pdf

Supporting Information

1 Syntheses 2
1.1 General Information 2
1.2 Synthesis of PIPER 2
1.3 Phosphoramidite Synthesis 3
1.4 Oligonucleotide Synthesis and Purification 3
2 Analytics of Oligonucleotides 5
2.1 Analytical RP-HPLC 5
2.2 ESI Mass Spectrometry 6
3 UV-VIS-based Thermal Denaturation Studies of G-Quadruplexes 8
3.1 Sample Preparation 8
3.2 Spectrometer and Methods 8
3.3 UV-VIS Spectra, Thermal Difference Spectra and Thermal Denaturation Profiles 9
4 CD Spectroscopy of G-Quadruplexes 16
4.1 Sample Preparation 16
4.2 Spectrometer and Methods 16
4.3 CD Spectra 16
4.4 Induced Circular Dichroism 18
5 EPR and PDEPR Spectroscopy 19
5.1 Sample Preparation 19
5.2 Spectrometer and Methods 19
5.3 EPR/DEER of Cu^{2+}-based Spin Labels 20
5.4 Comparison of Orientation-selective DEER and RIDME Datasets 26
5.5 Orientation-selective DEER Data 27
5.6 Structural Details of Dimers Obtained from PeldorFit Simulations 31
5.7 Obstruction of Dimer Formation 36
5.8 Intercalation of PIPER into 3’-3’ Stacked Dimers 37
5.9 No Intercalation of PIPER into 5’-5’ Stacked Dimers 41
5.10 Intercalation of Telomestatin into 3’-3’ Stacked Dimers 42
5.11 Intercalation of Free G-Tetrads into 3’-3’ Stacked Dimers 43
6 Molecular Dynamics (MD) Simulations 47
6.1 Generation of Missing Parameters 47
6.2 Generation of Initial Structures 48
6.3 MD Simulation Procedure 48
6.4 MD-derived Structures, Cu^{2+}–Cu^{2+} Distances and RMSD Trajectories 49
7 References 55

1 Syntheses

1.1 General Information

Chemicals and solvents were purchased from *Sigma Aldrich*, *Acros Organics*, *Carl Roth*, *TCI Europe*, *VWR*, *ABCR* or other suppliers and used as received. Telomestatin was kindly provided by Dr. Kazuo Shin-ya, National Institute of Advanced Industrial Science and Technology (AIST), Tokyo, and Prof. Dr. Takayuki Doi, Tohoku University, Sendai, Japan and purity was checked with mass spectrometry and UV spectroscopy. Ultrapure water (type I, 18.2 MΩ cm) was produced with a *VWR Puranity TU 3 UV* apparatus. Reactions under microwave irradiation were performed in a *CEM Discovery SP* microwave reactor. NMR spectra were recorded on a *Bruker AV 500 Avance NEO* spectrometer. For ^{1}H spectra, chemical shifts were calibrated to the solvent lock signal. Chemical shifts δ are given in ppm. Mass spectrometry was performed on *Bruker ESI-timsTOF* and *Compact* mass spectrometers. For calibration of the TOF device, *Agilent ESI-Low Concentration Tuning Mix* was used. Melting points were determined on a *Stuart SMP30* melting point apparatus.

1.2 Synthesis of PIPER

The G-quadruplex-binding ligand **PIPER** was synthesized and purified using modified published procedures.[1,2]

A mixture of perylene-3,4,9,10-tetracarboxylic dianhydride (1.00 g, 2.55 mmol, 1.0 equiv.) and 1-(2-aminoethyl)piperidine (0.80 mL, 5.61 mmol, 2.2 equiv.) in acetonitrile (10 mL) was stirred and irradiated in a sealed vessel in a microwave reactor (150 watts, 95 °C, 1.5 h). The obtained reaction mixture was extracted with chloroform (300 mL) and the solvent and traces of the primary amine were removed under reduced pressure. *N,N'*-bis[2-(1-piperidino)ethyl]-3,4,9,10-perylenetetra-carboxylic diimide (**1**) was obtained as a purple solid (1.31 g, 2.14 mmol, 84%) with a melting point >300 °C.

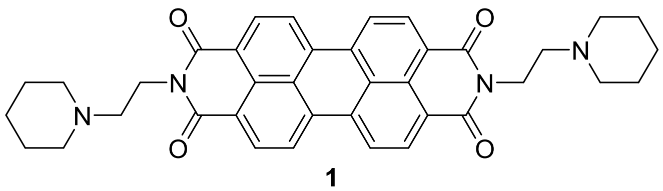

1

^{1}H NMR (500 MHz, chloroform-d): δ 8.66 (d, J = 7.9 Hz, 4H), 8.57 (d, J = 7.9 Hz, 4H), 4.38 (t, J = 7.2 Hz, 4H), 2.70 (t, J = 7.2 Hz, 4H), 2.63 – 2.51 (m, 8H), 1.64 – 1.58 (m, 8H), 1.48 – 1.41 (m, 4H).

HR-ESI MS (positive mode): *m/z* calc. for $C_{38}H_{37}N_4O_4$ 613.2809 $[M+H]^+$; found (%) 613.2802 (100).

Compound **1** (44 mg, 72 µmol, 1 equiv.) was treated with conc. aq. HCl (2 mL) and triturated with a glass rod until a bright brick red solid formed (~5 min). Water (1 mL) was added and the solid was collected by filtration, washed with ether (10 mL) and air-dried at 65 °C for 3 days. *N,N'*-bis[2-(1-piperidino)ethyl]-3,4,9,10-perylenetetracarboxylic diimide dihydrochloride (**PIPER**) was obtained as a dark red solid (46 mg, 67 µmol, 93%) with a melting point >300 °C.

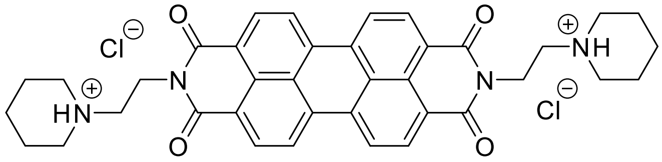

PIPER

^{1}H NMR (500 MHz, deuterium oxide): δ 8.20 – 6.40 (br m, 8H), 4.60 – 4.23 (br m, 4H), 3.95 – 3.65 (br m, 4H), 3.60 – 3.38 (br m, 4H), 3.32 – 3.02 (br m, 4H), 2.25 – 1.50 (br m, 12H).

HR-ESI MS (positive mode): *m/z* calc. for $C_{38}H_{38}N_4O_4$ 307.1441 $[M\text{-}2Cl]^{2+}$, $C_{38}H_{37}N_4O_4$ 613.2809 $[M\text{-}H\text{-}2Cl]^{+}$, $C_{76}H_{75}ClN_8O_8$ 631.2693 $[2M\text{-}H\text{-}3Cl]^{2+}$, $C_{76}H_{76}Cl_2N_8O_8$ 649.2576 $[2M\text{-}2Cl]^{2+}$, $C_{76}H_{73}N_8O_8$ 1225.5546 $[2M\text{-}3H\text{-}4Cl]^{+}$, $C_{76}H_{74}ClN_8O_8$ 1261.5313 $[2M\text{-}2H\text{-}3Cl]^{+}$; found (%) 307.1455 (100), 613.2821 (100), 631.2692 (7), 649.2570 (3), 1225.5536 (3), 1261.5302 (3).

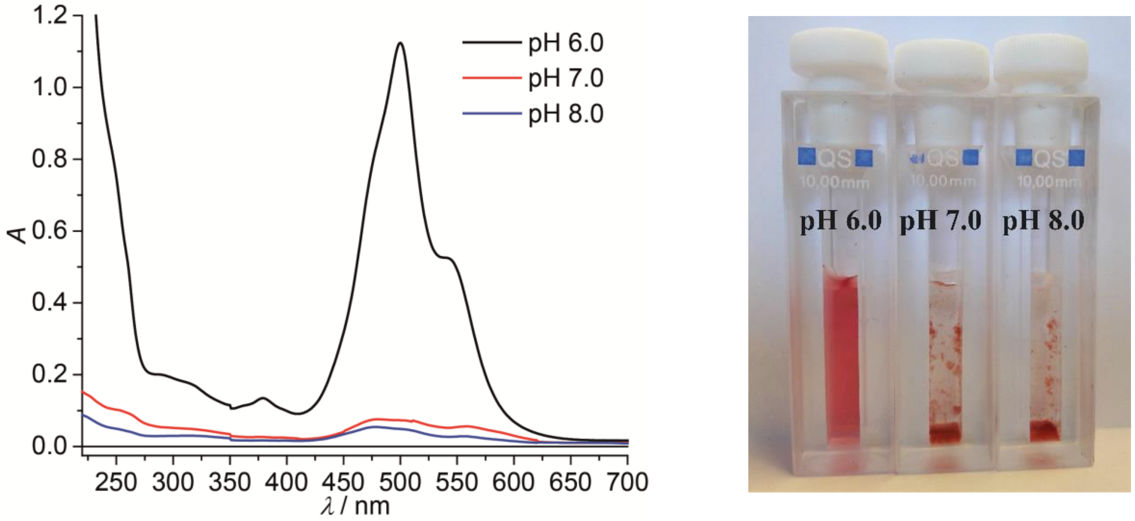

Fig. S1: UV-VIS spectra of 50 µM **PIPER** in 50 mM potassium phosphate buffer of different pH. **PIPER** is only soluble at pH 6. In buffered solutions at pH 7 and 8, **PIPER** is not soluble and precipitates.[3]

A 1 mM stock solution of the **PIPER** dihydrochloride in water was prepared and stored at 6 °C for several months.

1.3 Phosphoramidite Synthesis

The phosphoramidite building block for the artificial nucleotide **L** that was needed for DNA synthesis was synthesized as previously reported.[4] In contrast to earlier studies, only one enantiomer (*S*) of the artificial nucleotide **L** was used in this study.

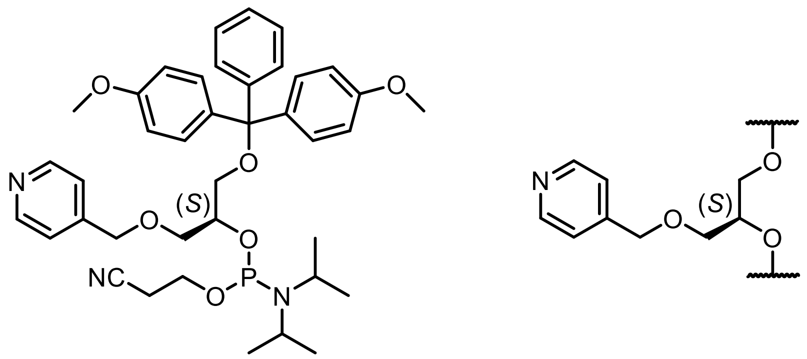

Fig. S2: Structural formulas of the phosphoramidite building block (left) and the corresponding artificial nucleoside **L** (right) as incorporated into oligonucleotides.

1.4 Oligonucleotide Synthesis and Purification

All oligonucleotides were synthesized on a *K&A Laborgeraete GbR H-8* synthesizer on a 1 µmol scale using the standard phosphoramidite methods on CPG and following previously published procedures for synthesis.[4,5]

Table 1: Reagents for DNA synthesis.

name	reagent	composition
DCA	detritylation	3% (v/v) dichloroacetic acid in anhydrous dichloromethane
ACT	activator	0.3 M 5-(benzylthio)-1H-tetrazole in anhydrous acetonitrile
Cap A	capping A	10% (v/v) N-methyl imidazole in anhydrous tetrahydrofurane
Cap B	capping B	2,6-lutidine / acetic anhydride / anhydrous tetrahydrofurane 1:1:8 (v/v/v)
OXI	oxidizer	0.02 M iodine in tetrahydrofurane / pyridine / water 7:2:1 (v/v/v)
ACN		anhydrous acetonitrile

Standard phosphoramidites (DMT-dT-CEP and DMT-dG(*i*Bu)-CEP) were used and the cartridges with controlled pore glass (CPG) solid supports (1000 Å, 25-35 µmol/g, DMT-dT-CPG and DMT-dG(*i*Bu)-CPG) were manually packed. The oligonucleotide synthesis followed the built-in methods of the DNA synthesizer and was slightly modified. First, the cartridges were treated three times with DCA to deprotect the 5'-OH groups. Second, coupling was achieved by mixing the respective phosphoramidite building block (0.1 M in ACN) with ACT (1:1, v/v). The coupling time was ~0.5 min for standard phosphoramidites and ~3.5 min for the ligand-modified phosphoramidite. Third, the cartridge was treated with a 1:1 (v/v) mixture of Cap A and Cap B to acetylate unreacted 5'-OH groups, which was followed, by the oxidation with OXI. Here, an additional washing step with ACN was introduced compared to the standard routine. After each individual step of the cycle, the cartridge was washed with ACN followed by a drying step with argon. The described cycle was repeated for every incorporated nucleotide.

After DNA synthesis, the solid supports were removed from the cartridges and treated with concentrated aqueous NH_3 solution at 55 °C overnight for cleavage and deprotection. The supernatant solution was filtered (*VWR Centrifugal filters*) and the solid support was washed with 100 µL H_2O. NH_3 was removed from the filtrate under reduced pressure using a *H. Saur Laborbedarf S-Concentrator BA-VC-300H* vacuum concentrator and the volume of the solution was reduced to ~300 µL.

Purification of the oligonucleotides was performed with reversed-phase HPLC on an *Agilent Technologies 1260 Infinity II* HPLC system equipped with an autosampler, column oven, DAD detector and a *Macherey-Nagel VP 250/10 NUCLEODUR 100-5 C18ec* column (oven temperature: 60 °C, flow rate: 2.5 mL/min, solvent A: 50 mM TEAA pH 7, solvent B: 70:30 MeCN/50 mM TEAA pH 7, gradient: from 100% solvent A to 20% solvent A and 80% solvent B in 30 min). To remove ACN from the sample, the volume of the solution was again reduced to ~300 µL in the vacuum concentrator and then the sample was diluted with 100 mM TEAA pH 7 to a volume of 2 mL.

Subsequently, the cleavage of the 5′-OH DMT protecting groups (with 2% TFA) and desalting were accomplished using *Waters Sep-Pak* C18 cartridges. Desalted oligonucleotides were lyophilized using a *Christ Alpha 2-4 LSCbasic* lyophilisation device and stored either as a solid or as 0.5–2.5 mM stock solutions in different buffers (100 mM lithium cacodylate pH 7.2, 75 mM potassium phosphate pH 7.0) at –20 °C.

The concentrations of all oligonucleotide stock solutions were determined *via* the absorbance at 260 nm at 25 °C with a *Thermo Scientific Nanodrop One* instrument and using revised extinction coefficients for the nucleosides.[4,6]

2 Analytics of Oligonucleotides

2.1 Analytical RP-HPLC

To check the purity of the synthesized and purified oligonucleotides, samples (10 µL) with DNA concentrations of around 500 µM in 20 mM TMAA pH 7 or TEAA pH 7 were prepared and analytical RP-HPLC was performed on an *Agilent Technologies 1260 Infinity II* system equipped with an autosampler, column oven, DAD detector and a *Macherey-Nagel EC 250/4.6 NUCLEODUR 100-5 C18ec* column (oven temperature: 60 °C, flow rate: 0.75 mL/min, solvent A: 50 mM TEAA pH 7, solvent B: 70:30 MeCN/50 mM TEAA pH 7). The RP-HPLC traces including the used solvent gradient are shown below. Purity is given in percent relative to the trace total peak areas.

Oligo A (5'-TT**L** GGG (95%))

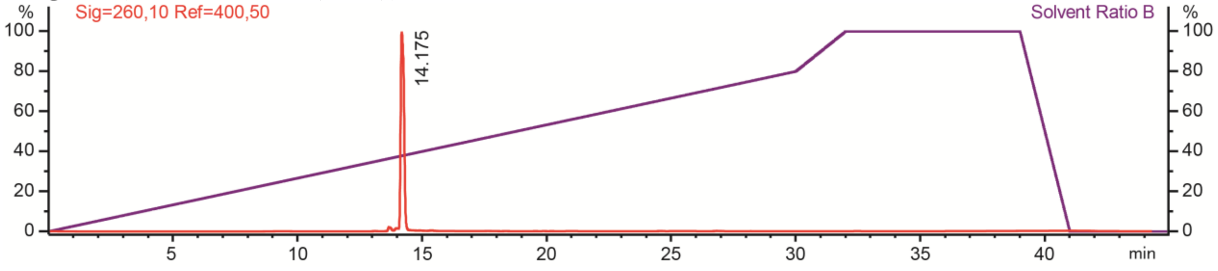

Oligo B (5'-T**L**G GGG (96%))

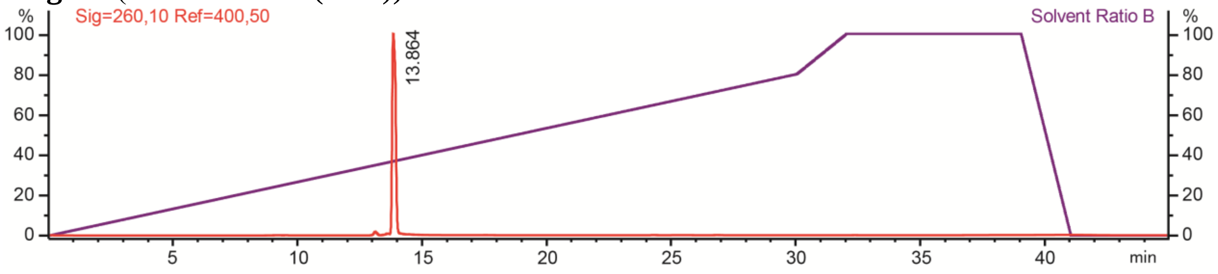

Oligo C (5'-TT**L** GGG T (91%))

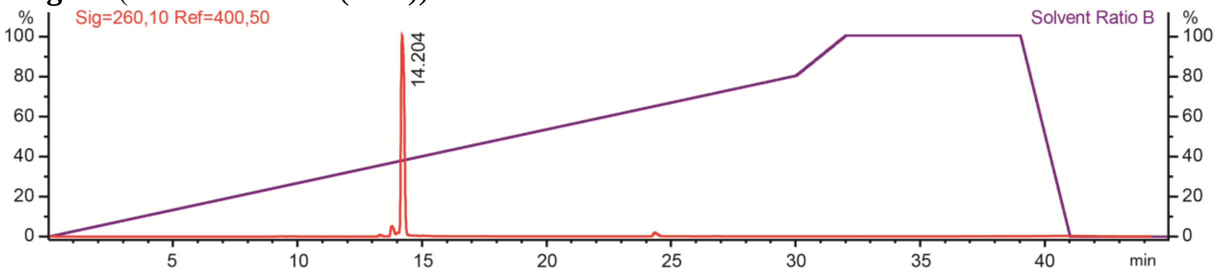

Oligo D (5'-GGG **L**TT (95%))

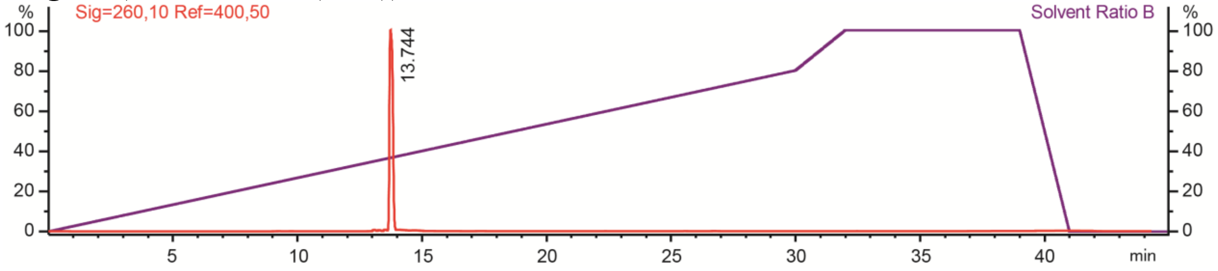

Oligo E (5'-GGG G**L**T (92%))

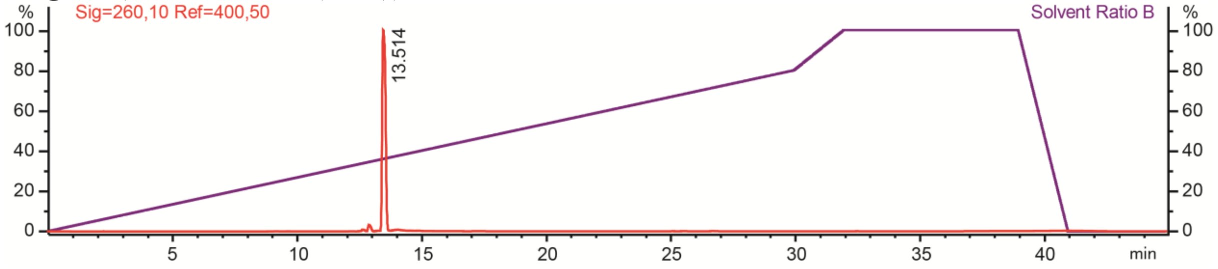

Oligo F (5'-TGG G**L**T T (93%))

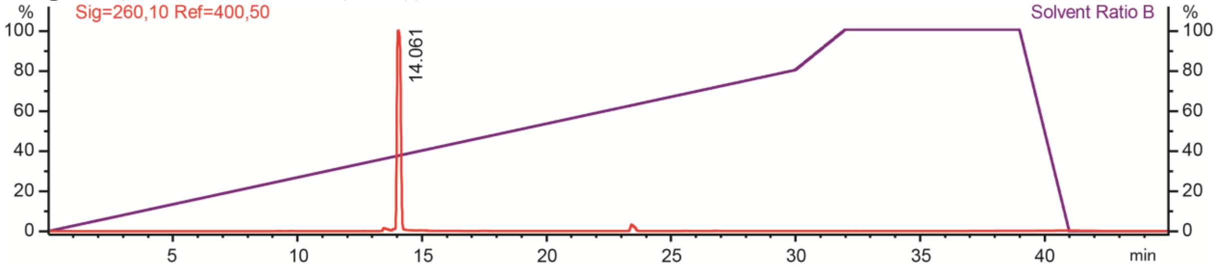

2.2 ESI Mass Spectrometry

To further check the purity of the oligonucleotides, samples (5 µL) with DNA concentrations of 150 – 300 µM in 15 mM TMAA pH 7 or TEAA pH 7 were prepared. Mass spectrometry was then performed on *Bruker ESI-timsTOF* and *Compact* mass spectrometers (negative mode). For calibration of the TOF device, *Agilent ESI-Low Concentration Tuning Mix* was used. Automatic injection of the samples was achieved with the autosampler of an *Agilent Technologies 1260 Infinity* system (flow rate: 0.3 mL/min, solvent: MeCN/H_2O 1:1, v/v). The ESI mass spectra are shown below.

Oligo A (5'-TT**L** GGG)

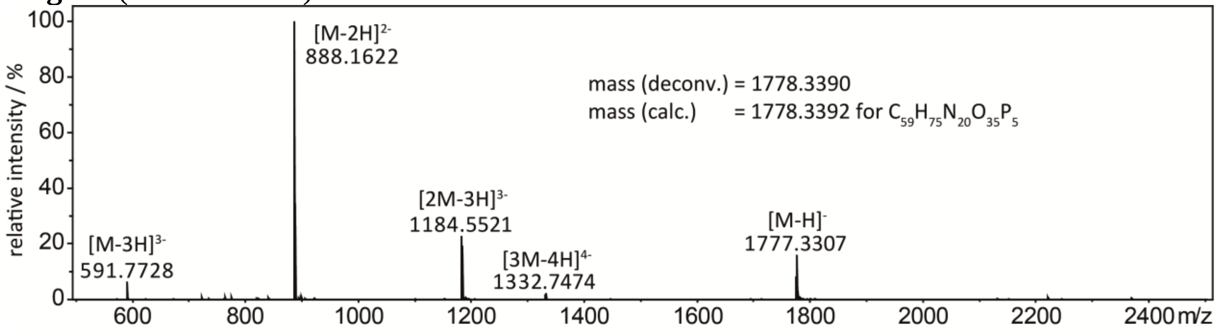

Oligo B (5'-T**L**G GGG)

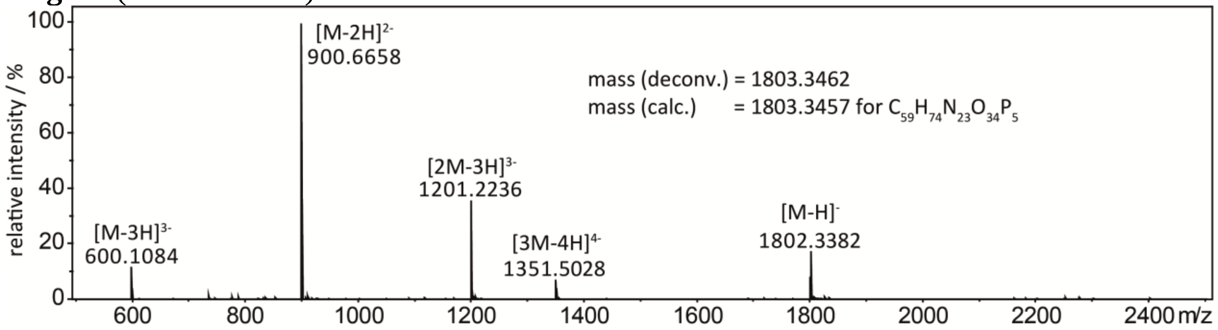

Oligo C (5’-TT**L** GGG T)

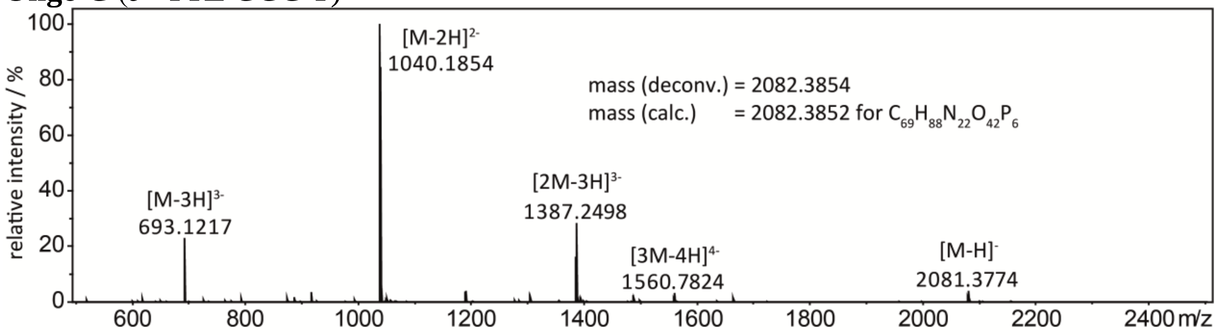

Oligo D (5’-GGG **L**TT)

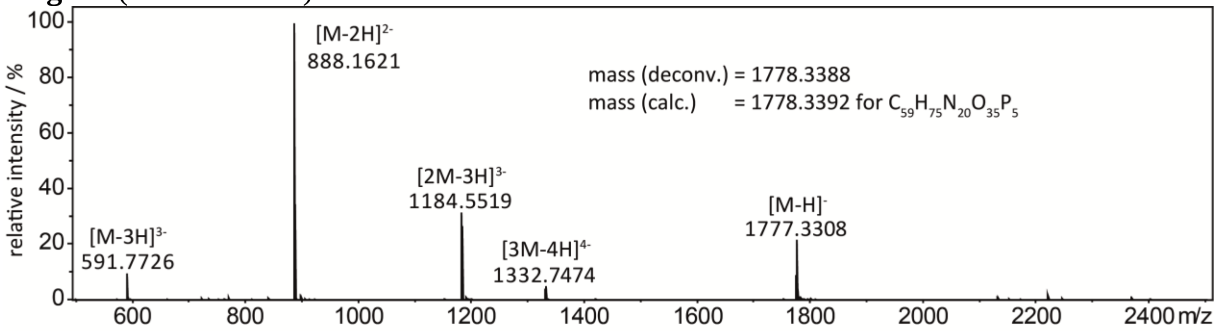

Oligo E (5’-GGG G**L**T)

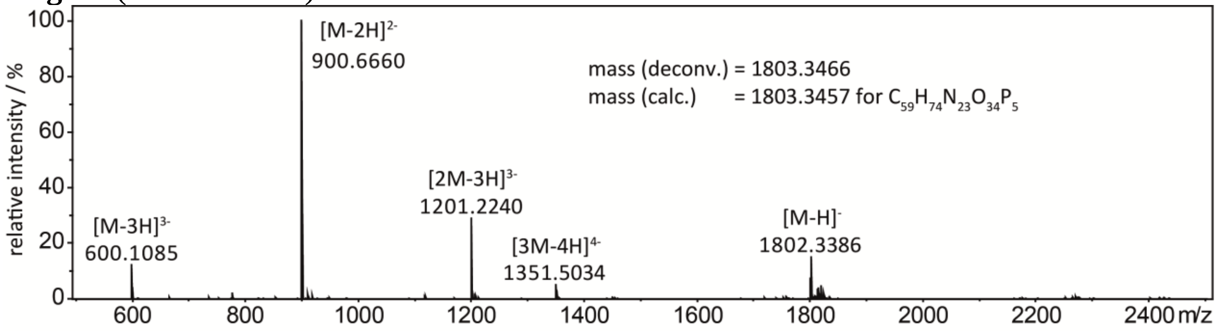

Oligo F (5’-TGG G**L**T T)

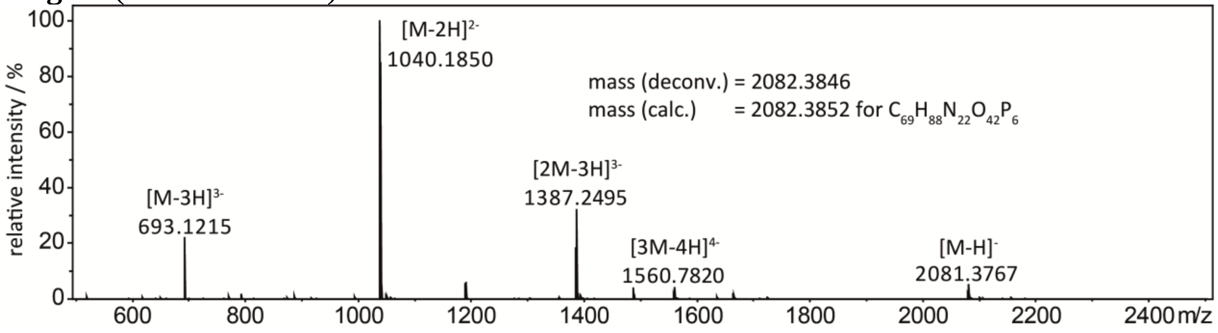

The purity of the oligonucleotides was also regularly checked after longer storage with mass spectrometry. Therefore, stock solutions were desalted with *Millipore Ziptips 0.6 µL* C_{18}.

3 UV-VIS-Based Thermal Denaturation Studies of G-Quadruplexes

3.1 Sample Preparation

For all UV-VIS-based thermal denaturation studies, G-quadruplex samples contained 16 µM single-stranded DNA (4 µM G-quadruplex DNA), 100 mM KCl or NaCl, 10 mM lithium cacodylate buffer pH 7.2 and, if present, 4 µM $CuSO_4$ or/and 2 µM PIPER. For all experiments, samples were prepared with ultrapure water (type I, 18.2 MΩ cm), obtained with a *VWR Puranity TU 3 UV*.
Samples were heated to 85 °C for 10 min, slowly cooled to 4 °C with a cooling rate of 0.5 °C/min and then left at this temperature for several hours (typically overnight). To ensure full formation of G-quadruplexes, the samples were frozen at –20 °C for 1 h[7] and thawed again to 4 °C.

3.2 Spectrometer and Methods

Both UV-VIS spectra and thermal denaturation profiles (melting curves) were recorded on a *Jasco V-750 UV-Visible Spectrophotometer* equipped with a *PAC-743 6-cell thermostat* for temperature control. The temperature was measured in the measurement cell in a water-filled cuvette. Quartz glass cuvettes (*Hellma Analytics114-QS*, 1 cm path length) were used. In order to avoid condensation of water on the cuvette surface or cell window at low temperatures, a constant flow of air was pumped through the measurement cell. Evaporation of water at high temperatures and resulting changes in the absorption behaviour were minimized by a thin layer of silicon oil placed onto the sample and by tightly stoppering the cuvette.
UV-VIS spectra were recorded from 700 to 220 nm with a scan rate of 200 nm/min both before (4 °C) and after thermal denaturation (95 °C). The data interval was set to 1 nm, bandwidth to 2.0 nm and the response time to 0.96 sec. All UV-VIS spectra were background corrected (cuvette, buffer and electrolyte) and zeroed using the absorption at 700 nm. To obtain the thermal difference spectra (TDS), the spectrum before denaturation (at 4 °C) was subtracted from the one after denaturation (at 95 °C). A negative band (hypochromic shift) at 295±2 nm and positive bands at 243±2 nm and 273±2 nm (hyperchromic shift) indicated G-quadruplex formation.[8]
For the thermal denaturation profiles (melting curves), absorption of the samples at 295 nm was recorded in a 0.5 °C interval with a temperature gradient set to 0.5 °C/min, which corresponds to ~0.174 °C/min including the measurement time. Data points were recorded from 4 °C to 95 °C. Melting curves were background corrected using the absorption at 700 nm and converted to the fraction folded values by linear fitting of the low and high temperature baselines.[9]

3.3 UV-VIS Spectra, Thermal Difference Spectra and Thermal Denaturation Profiles

Oligo A (5’-TT**L** GGG) in KCl

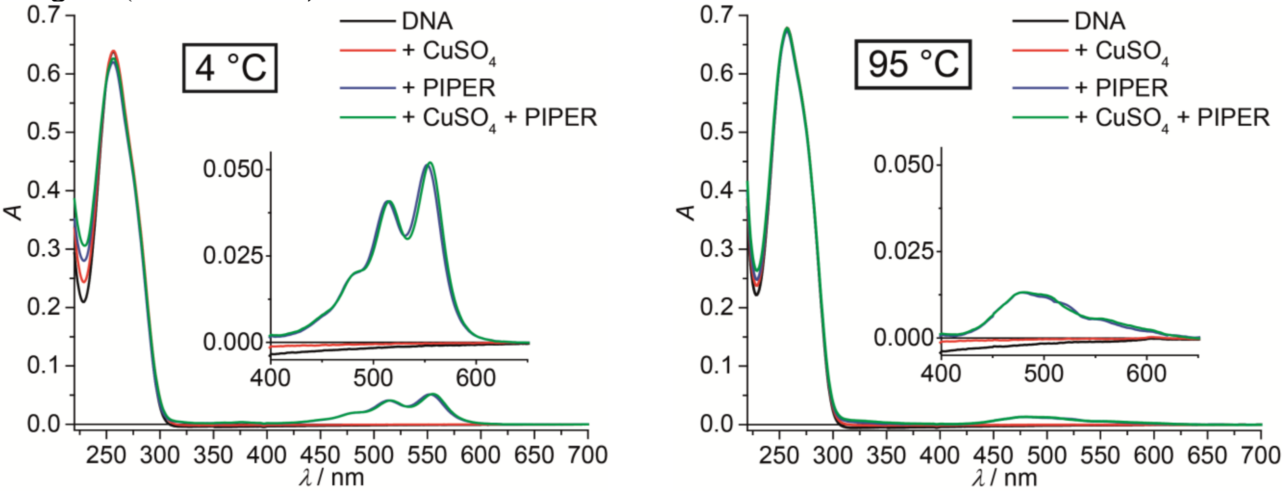

Fig. S3: UV-VIS spectra of folded (4 °C, left) and unfolded (95 °C, right) G-quadruplex (TT**L** GGG)$_4$ in absence or presence of 1 equiv. of $CuSO_4$ or/and 0.5 equiv. of PIPER.

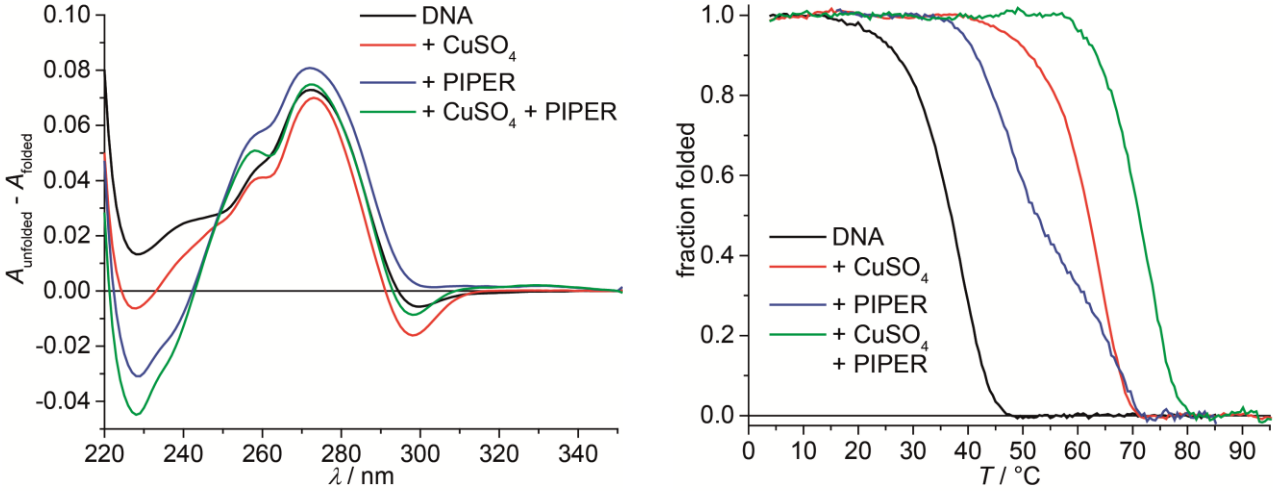

Fig. S4: Thermal difference spectra (left) and thermal denaturation profiles (right) of G-quadruplex (TT**L** GGG)$_4$ in absence or presence of 1 equiv. of $CuSO_4$ or/and 0.5 equiv. of PIPER.

Oligo B (5’-T**L**G GGG) in KCl

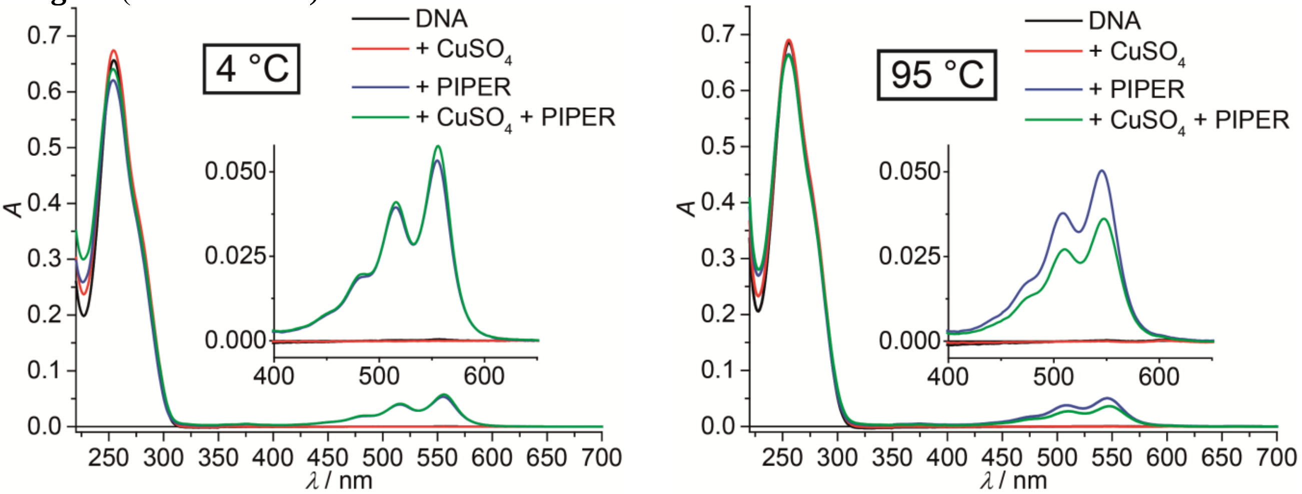

Fig. S5: UV-VIS spectra of folded G-quadruplex (T**L**G GGG)$_4$ at 4 °C (left) and at 95 °C (right) in KCl containing solution in absence or presence of 1 equiv. of $CuSO_4$ or/and 0.5 equiv. of PIPER. The thermal denaturation temperatures are >95 °C.

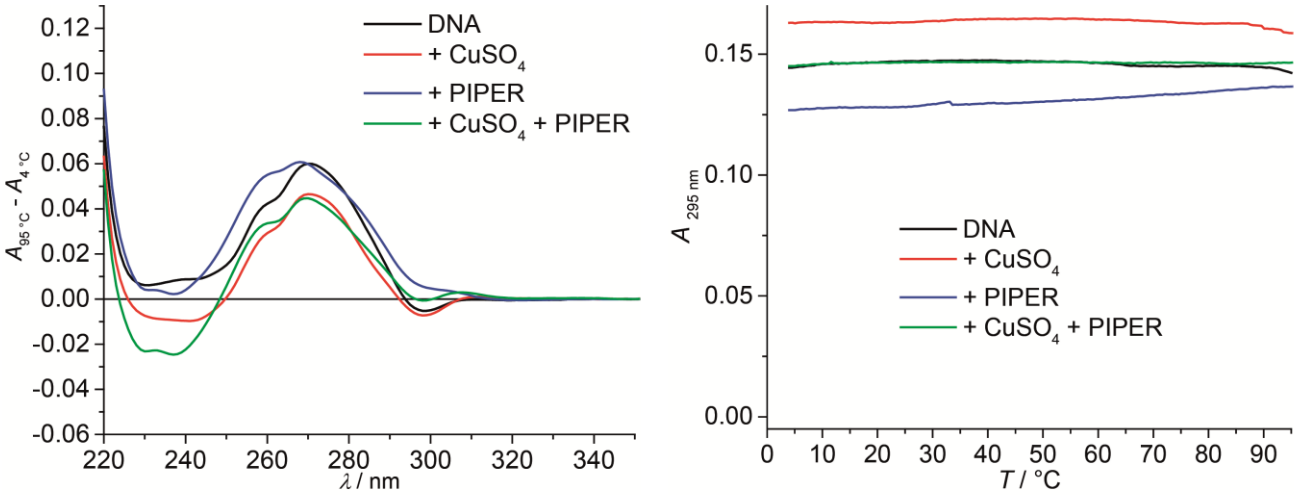

Fig. S6: Thermal difference spectra (left) and temperature-dependent absorption at 295 nm (right) of G-quadruplex (T**L**G GGG)$_4$ in KCl containing solution in absence or presence of 1 equiv. of $CuSO_4$ or/and 0.5 equiv. of PIPER. The thermal denaturation temperatures are >95 °C.

Oligo B (5'-T**L**G GGG) in NaCl

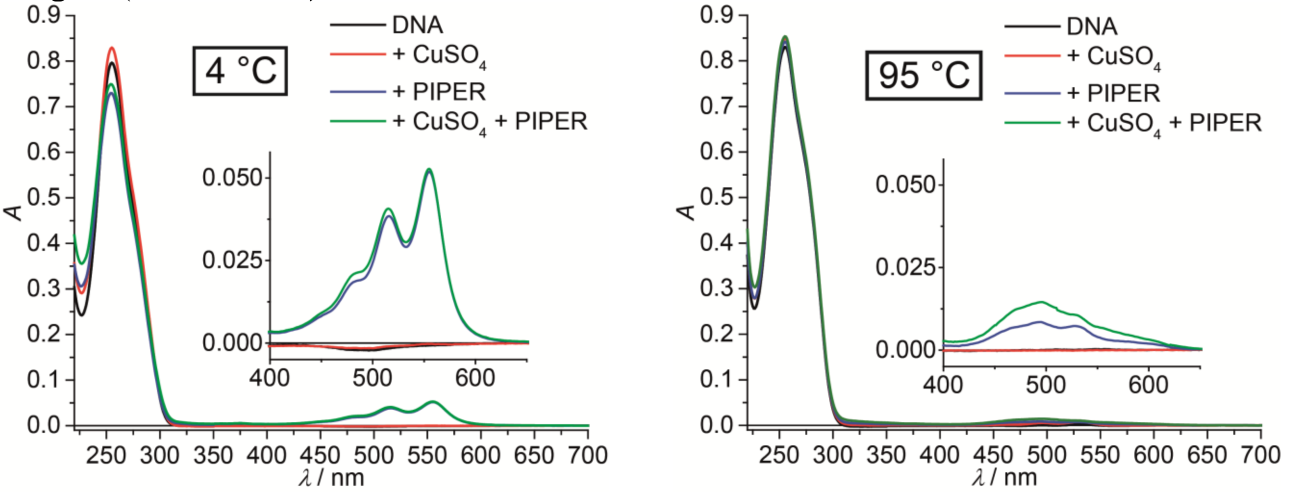

Fig. S7: UV-VIS spectra of folded (4 °C, left) and unfolded (95 °C, right) G-quadruplex (T**L**G GGG)$_4$ in NaCl containing solution in absence or presence of 1 equiv. of $CuSO_4$ or/and 0.5 equiv. of PIPER.

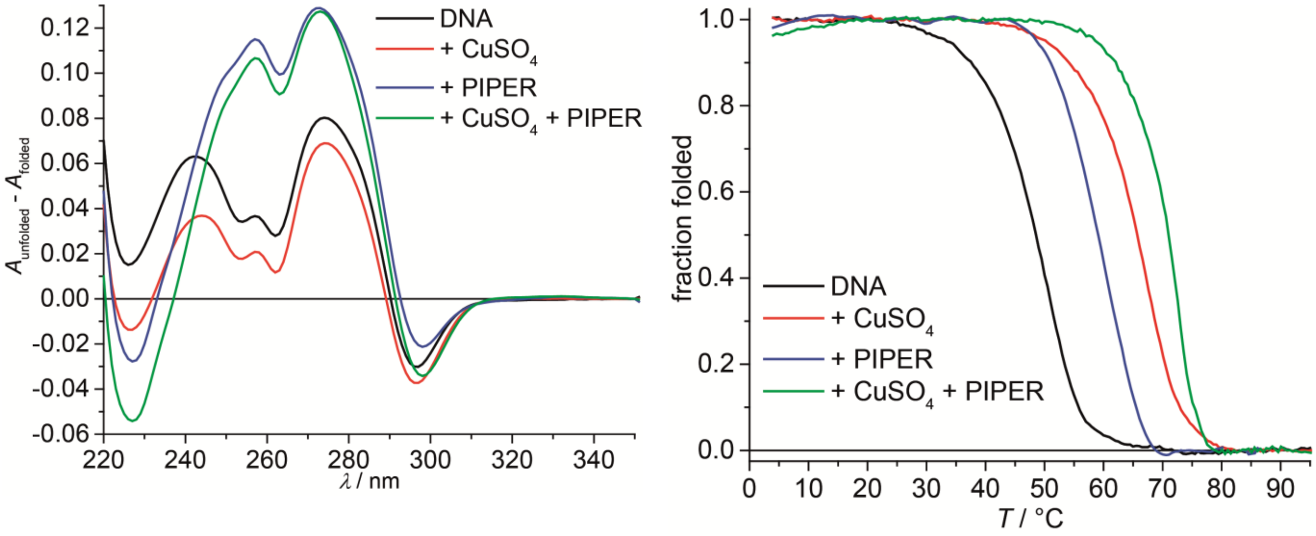

Fig. S8: Thermal difference spectra (left) and thermal denaturation profiles (right) of G-quadruplex (T**L**G GGG)$_4$ in NaCl containing solution in absence or presence of 1 equiv. of $CuSO_4$ or/and 0.5 equiv. of PIPER.

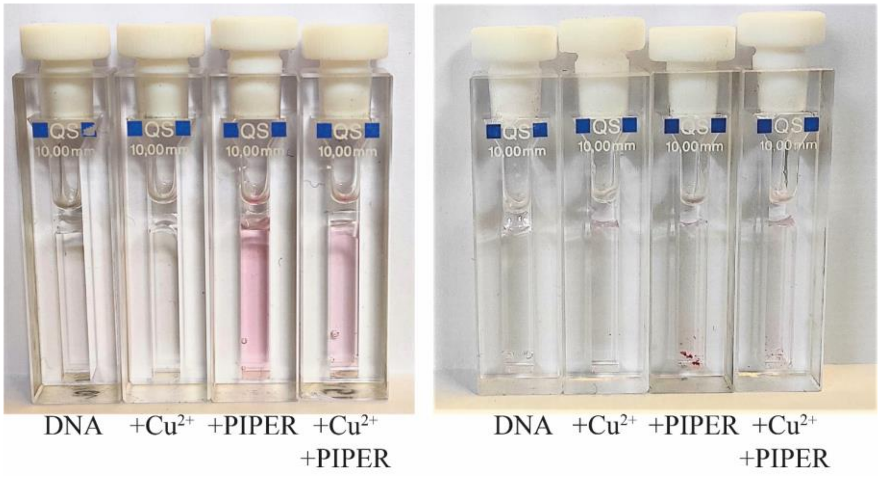

Fig. S9: Samples of quadruplex (5'-T**L**G GGG)$_4$ in KCl (left) or NaCl (right) containing solution after heating to 95 °C and cooling to r.t.. G-quadruplex (T**L**G GGG)$_4$ does not denature in presence of KCl and keeps PIPER in solution (red colored solutions, left). On the contrary, G-quadruplex (T**L**G GGG)$_4$ denatures at 95 °C in presence of NaCl and, hence, PIPER precipitates (red precipitate, right).

Oligo C (5’-TT**L** GGG T) in KCl

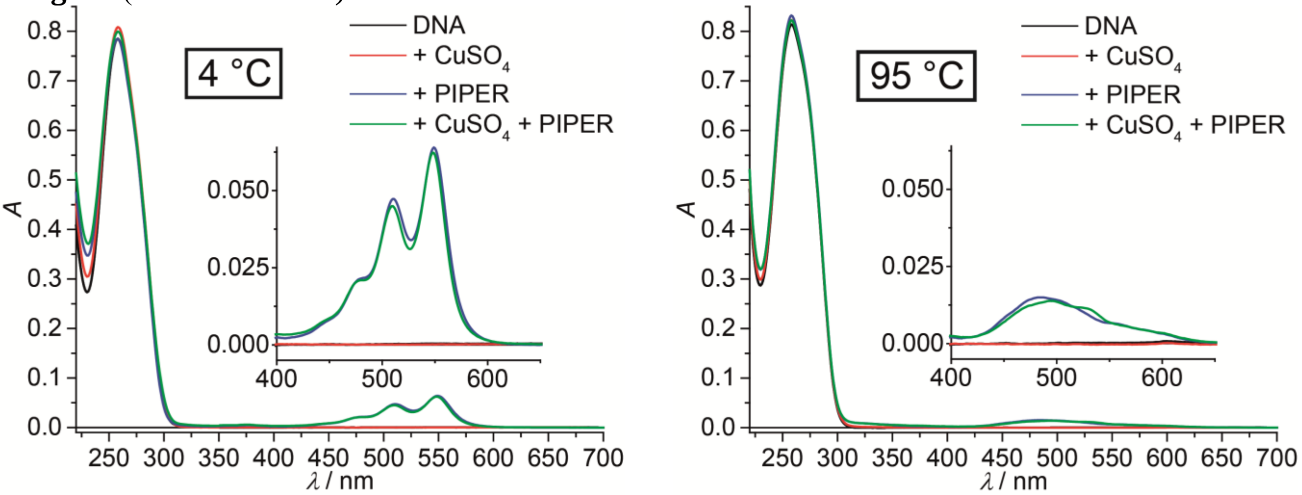

Fig. S10: UV-VIS spectra of folded (4 °C, left) and unfolded (95 °C, right) G-quadruplex (TT**L** GGG T)$_4$ in absence or presence of 1 equiv. of $CuSO_4$ or/and 0.5 equiv. of PIPER.

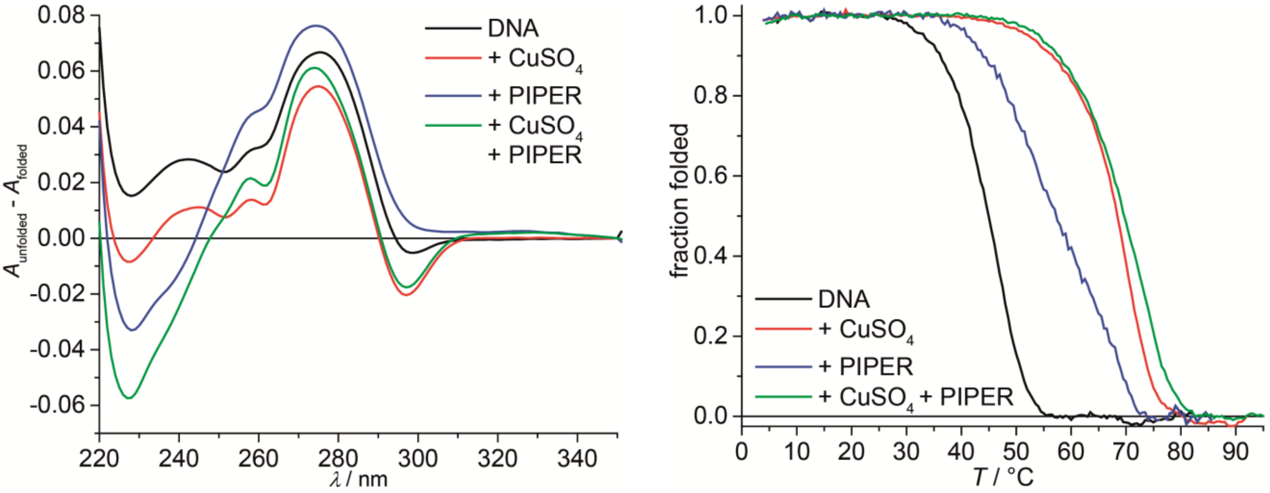

Fig. S11: Thermal difference spectra (left) and thermal denaturation profiles (right) of G-quadruplex (TT**L** GGG T)$_4$ in absence or presence of 1 equiv. of $CuSO_4$ or/and 0.5 equiv. of PIPER.

Oligo D (5’- GGG **L**TT) in KCl

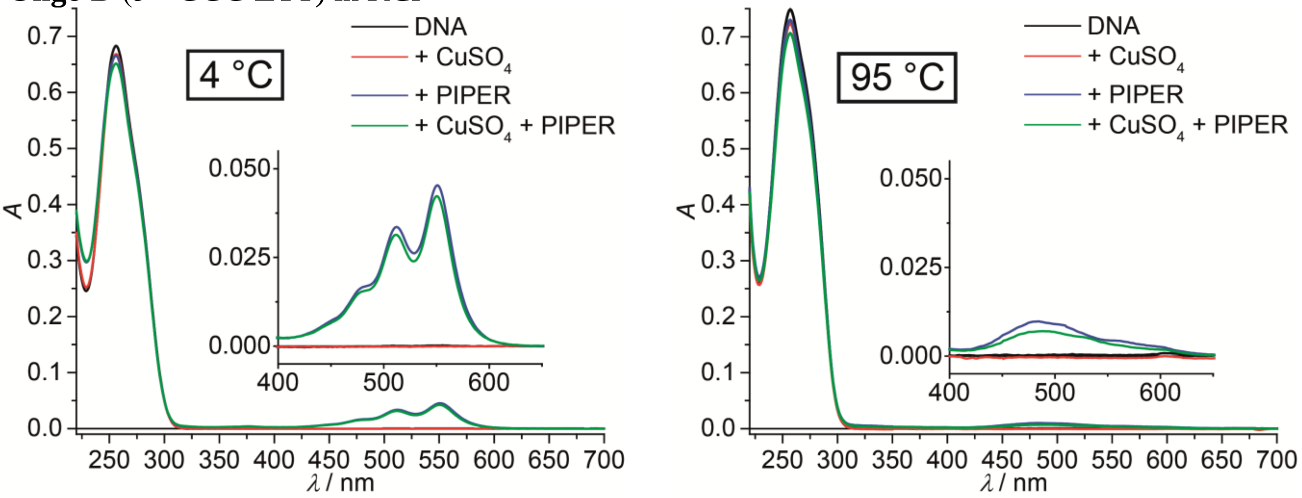

Fig. S12: UV-VIS spectra of folded (4 °C, left) and unfolded (95 °C, right) G-quadruplex (GGG **L**TT)$_4$ in absence or presence of 1 equiv. of $CuSO_4$ or/and 0.5 equiv. of PIPER.

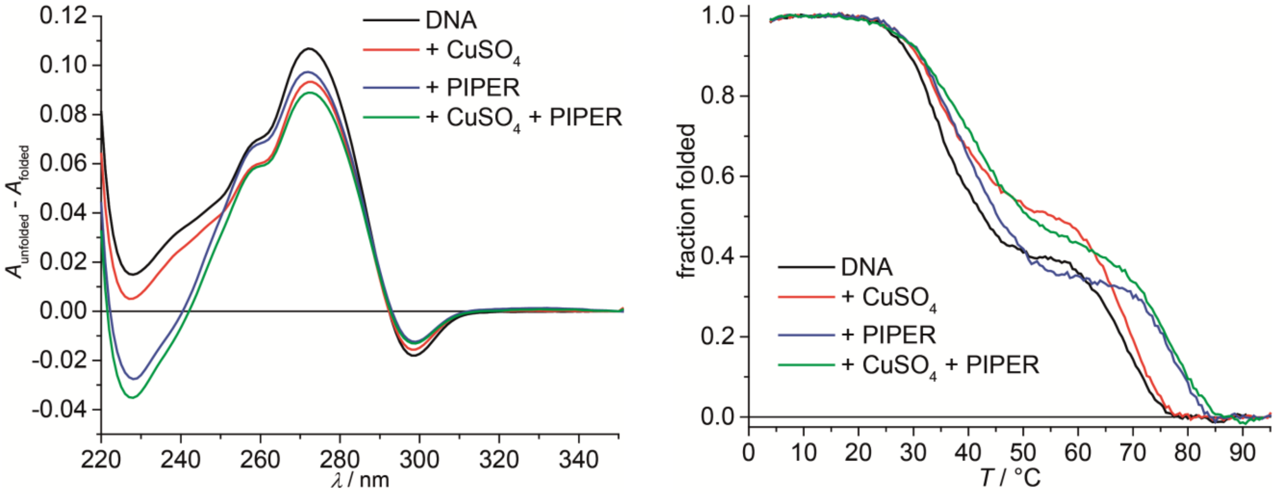

Fig. S13: Thermal difference spectra (left) and thermal denaturation profiles (right) of G-quadruplex (GGG **L**TT)$_4$ in absence or presence of 1 equiv. of $CuSO_4$ or/and 0.5 equiv. of PIPER.

G-quadruplexes containing oligo **D** show biphasic denaturation profiles suggesting either a more complicated, stepwise denaturation process of a single topology or the presence of a mixture of topologies (the usual parallel tetramolecular G-quadruplex and a different species) with different thermal stabilities in solution. The latter explanation is compatible with a subsequent observation in EPR-based distance measurements of respective dimeric quadruplex species [Cu^{2+}@**D**$_4$]$_2$, where a low modulation depth indicates that only a small fraction of G-quadruplex formed dimers (Figure S32). A different species that is unable to form dimers might be present.

Oligo E (5'-GGG G**L**T) in KCl

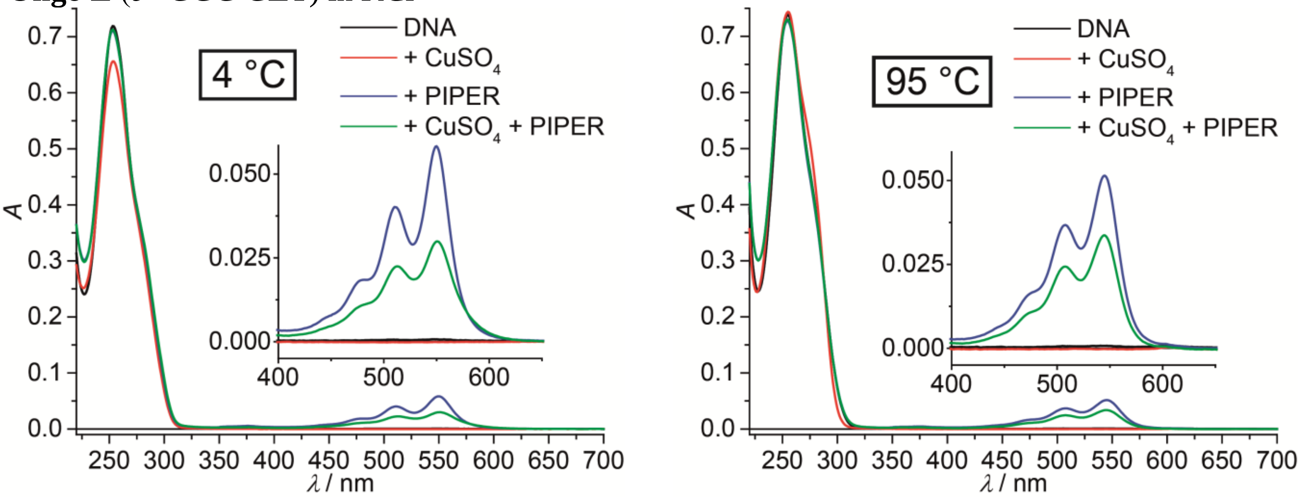

Fig. S14: UV-VIS spectra of folded G-quadruplex (GGG G**L**T)$_4$ at 4 °C (left) and at 95 °C (right) in KCl containing solution in absence or presence of 1 equiv. of $CuSO_4$ or/and 0.5 equiv. of PIPER. The thermal denaturation temperature is >95 °C.

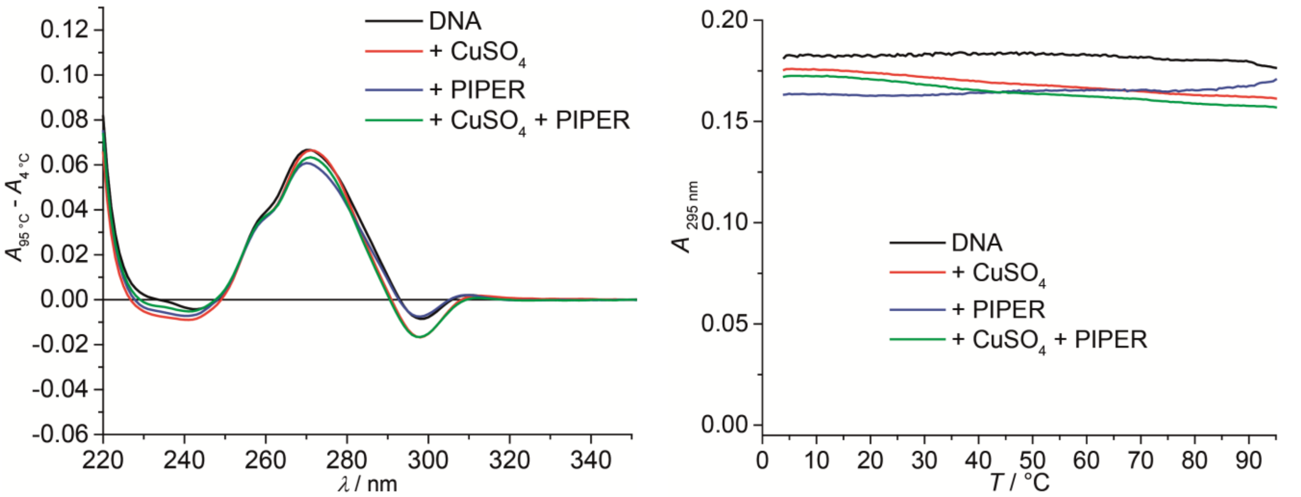

Fig. S15: Thermal difference spectra (left) and temperature-dependent absorption at 295 nm (right) of G-quadruplex (GGG G**L**T)$_4$ in KCl containing solution in absence or presence of 1 equiv. of $CuSO_4$ or/and 0.5 equiv. of PIPER. The thermal denaturation temperature is >95 °C.

Oligo E (5’- GGG G**L**T) in NaCl

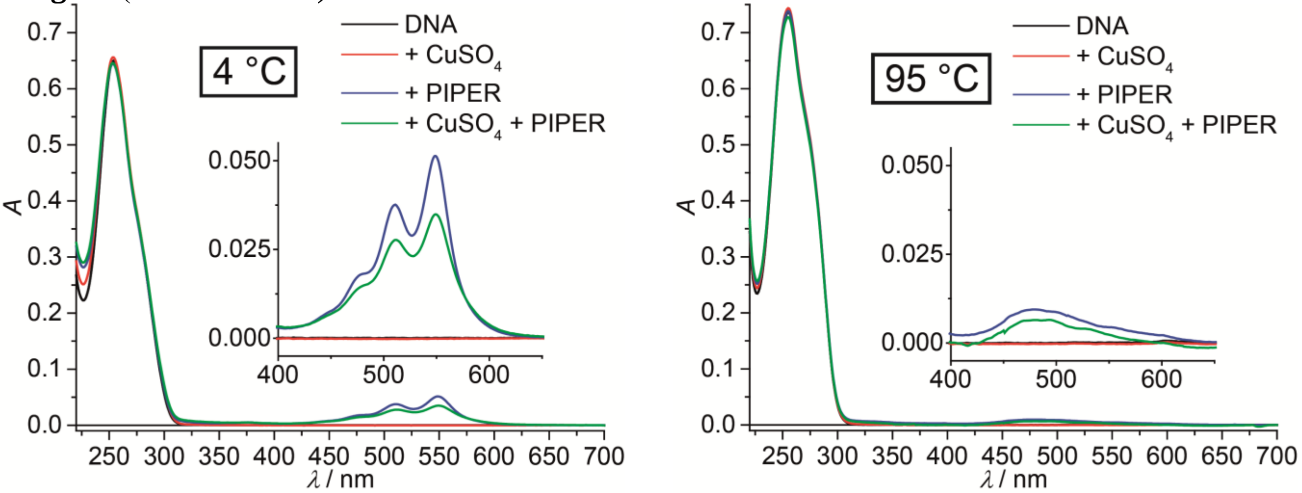

Fig. S16: UV-VIS spectra of folded (4 °C, left) and unfolded (95 °C, right) G-quadruplex (GGG G**L**T)$_4$ in NaCl containing solution in absence or presence of 1 equiv. of $CuSO_4$ or/and 0.5 equiv. of PIPER.

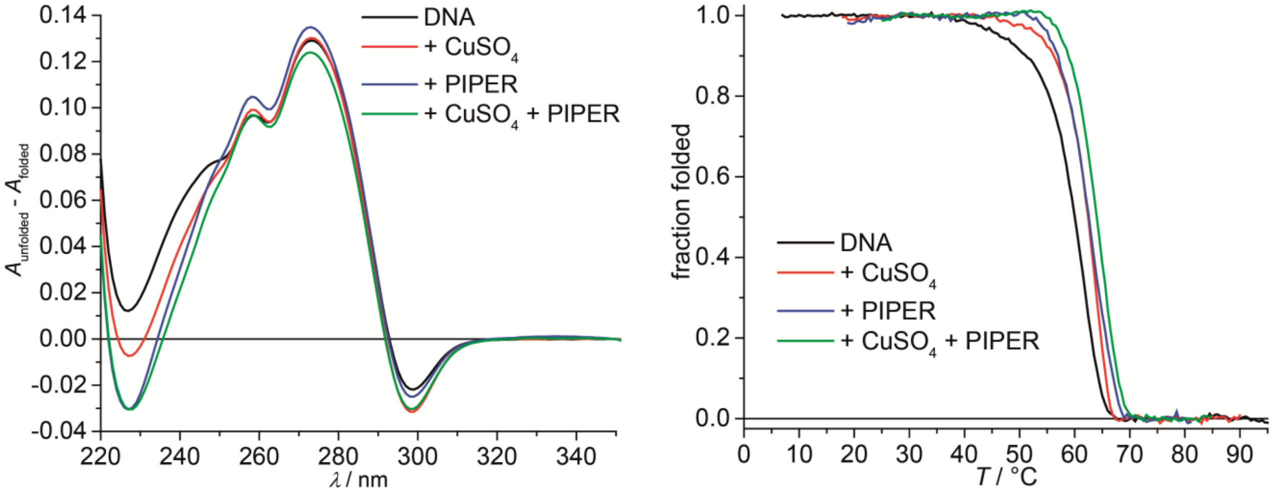

Fig. S17: Thermal difference spectra (left) and thermal denaturation profiles (right) of G-quadruplex (GGG G**L**T)$_4$ in NaCl containing solution in absence or presence of 1 equiv. of $CuSO_4$ or/and 0.5 equiv. of PIPER.

Oligo F (5’-TGG G**L**T T) in KCl

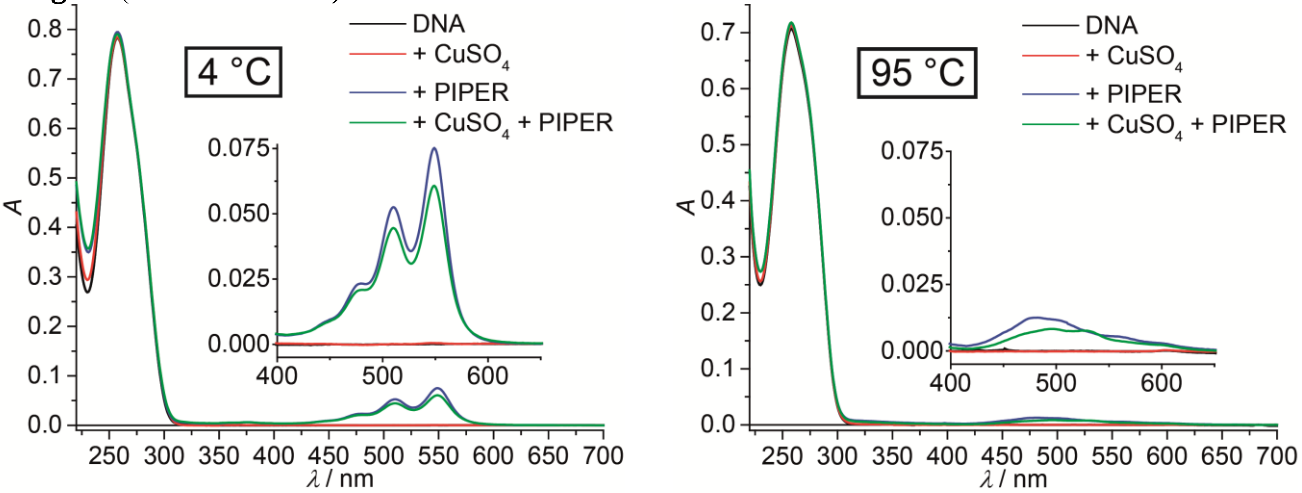

Fig. S18: UV-VIS spectra of folded (4 °C, left) and unfolded (95 °C, right) G-quadruplex (TGG G**L**T T)$_4$ in absence or presence of 1 equiv. of $CuSO_4$ or/and 0.5 equiv. of PIPER.

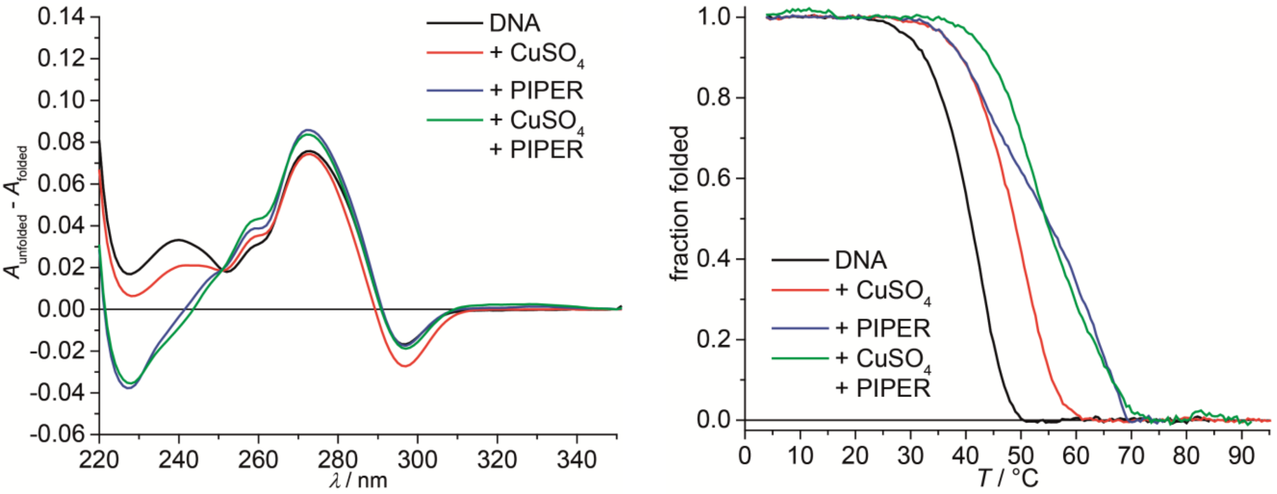

Fig. S19: Thermal difference spectra (left) and thermal denaturation profiles (right) of G-quadruplex (TGG G**L**T T)$_4$ in absence or presence of 1 equiv. of $CuSO_4$ or/and 0.5 equiv. of PIPER.

4 CD Spectroscopy of G-Quadruplexes

4.1 Sample Preparation

For standard CD measurements, the samples were prepared in the same way as for the UV-VIS-based thermal denaturation studies (chapter 3.1).

4.2 Spectrometer and Methods

CD spectra were measured on an *Applied Photophysics Chirascan qCD spectropolarimeter* (350 – 205 nm, 0.5 s time-per-point, step size 1 nm, bandwidth 0.5 nm, 3 repeats) at 4–7 °C. Temperature was controlled using a *Quantum Northwest* temperature control attached to a sample probe. The background was measured in the same cuvette as the sample. To avoid condensation of water onto the cuvette surface or cell window, a constant nitrogen gas flow was maintained.

All spectra were averaged, background corrected (cuvette, buffer and electrolyte), smoothed (Savitzky-Golay, window size 5) and zeroed to the signal at 350 nm.

4.3 CD Spectra

Oligo A (5’-TT**L** GGG) in KCl **Oligo C** (5’-TT**L** GGG T) in KCl

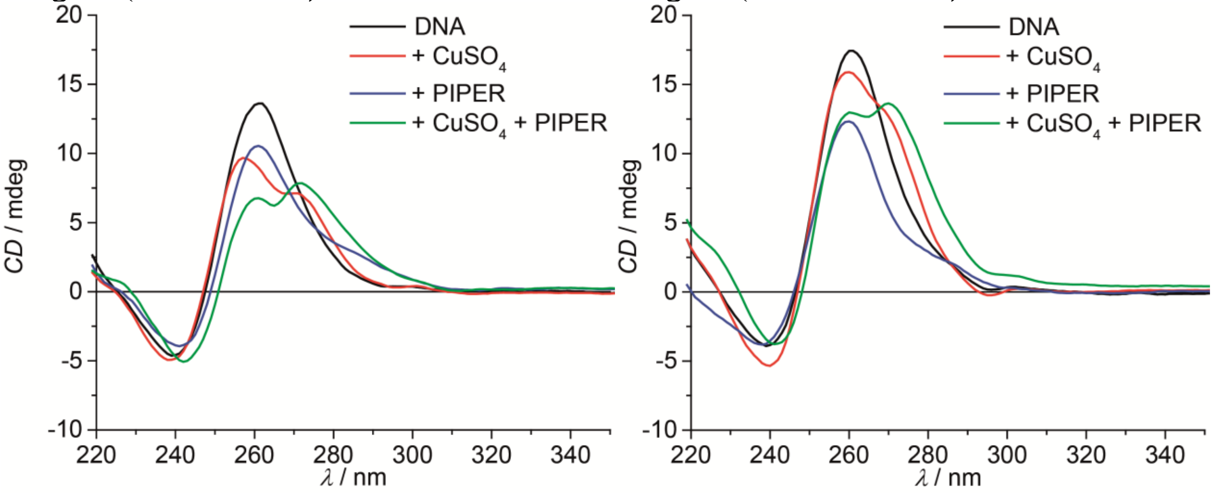

Fig. S20: CD spectra of folded G-quadruplex (TT**L** GGG)$_4$ (left) and (TT**L** GGG T)$_4$ (right) in absence or presence of 1 equiv. of $CuSO_4$ or/and 0.5 equiv. of PIPER.

Oligo B (5'-T**L**G GGG) in KCl **Oligo B** (5'-T**L**G GGG) in NaCl

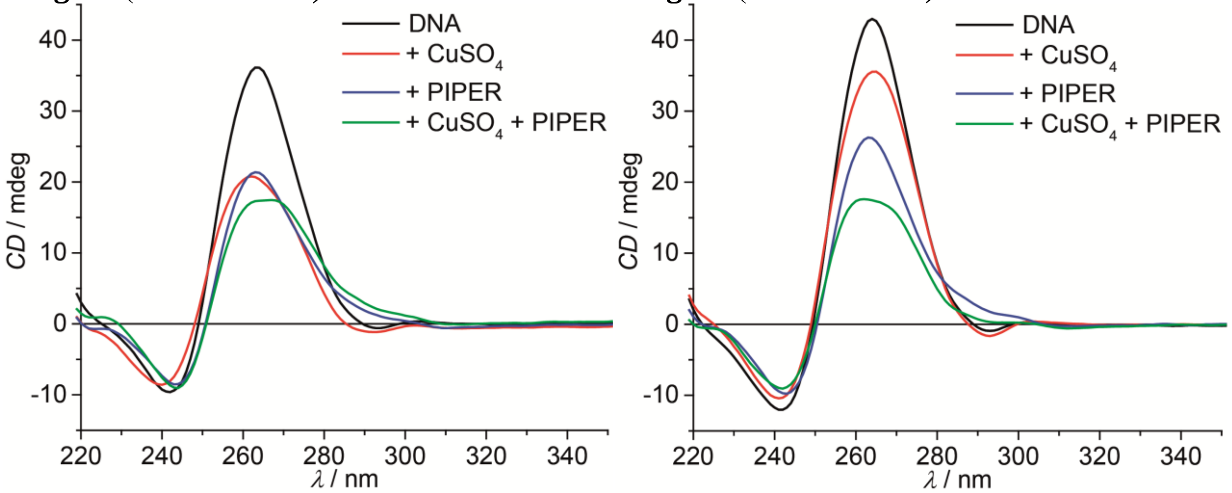

Fig. S21: CD spectra of folded G-quadruplex (T**L**G GGG)$_4$ in KCl (left) and NaCl (right) containing solution in absence or presence of 1 equiv. of $CuSO_4$ or/and 0.5 equiv. of PIPER.

Oligo D (5'-GGG **L**TT) in KCl **Oligo F** (5'-TGG G**L**T T) in KCl

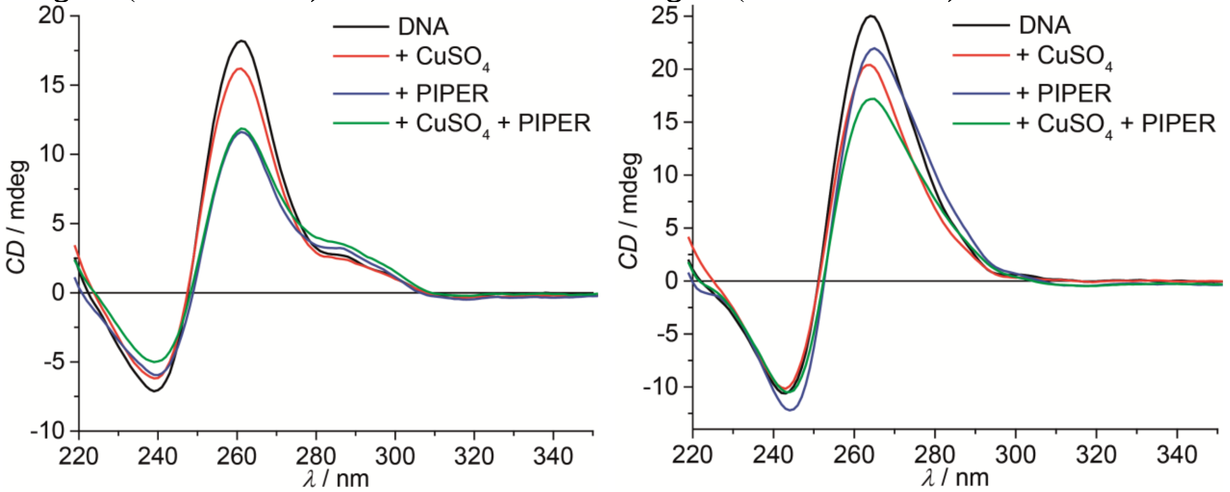

Fig. S22: CD spectra of folded G-quadruplex (GGG **L**TT)$_4$ (left) and (TGG G**L**T T)$_4$ (right) in absence or presence of 1 equiv. of $CuSO_4$ or/and 0.5 equiv. of PIPER.

Oligo E (5'-GGG G**L**T) in KCl **Oligo E** (5'-GGG G**L**T) in NaCl

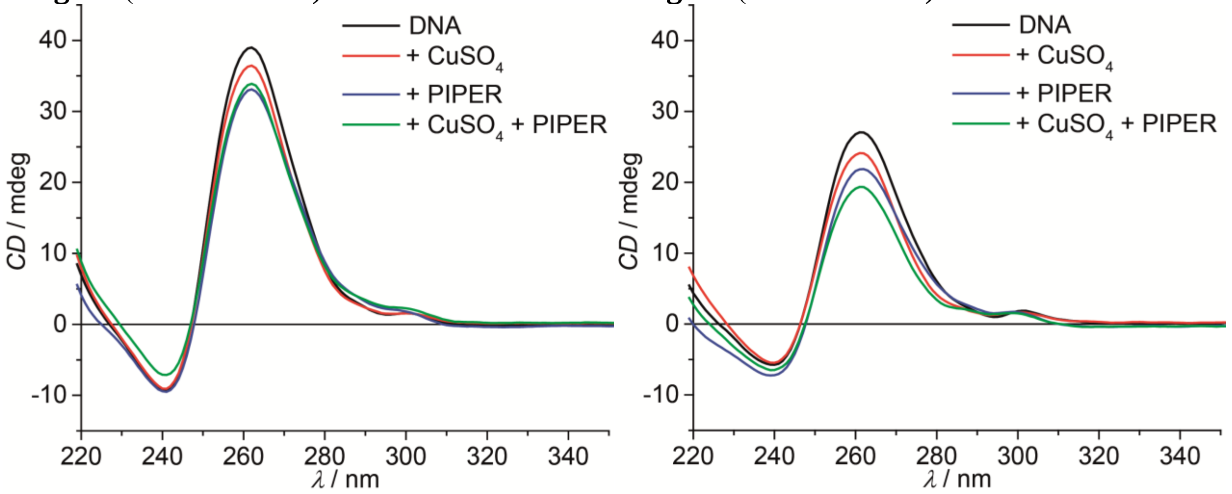

Fig. S23: CD spectra of folded G-quadruplex (GGG G**L**T)$_4$ in KCl (left) and NaCl (right) containing solution in absence or presence of 1 equiv. of $CuSO_4$ or/and 0.5 equiv. of PIPER.

4.4 Induced Circular Dichroism

G-quadruplex samples with higher concentration containing 80 µM single-stranded DNA (20 µM G-quadruplex DNA), 100 mM KCl, 10 mM lithium cacodylate buffer pH 7.2, 20 µM $CuSO_4$ and 10 µM PIPER were prepared. G-quadruplex formation was induced as described before (chapter 3.1) and CD spectra were recorded as described (chapter 4.2) but in a range of 700 – 205 nm.

CD bands were observed in the visible range of 400 – 650 nm. The chiral quadruplex DNA does not absorb light in this region, but PIPER does. The bands can be explained by induced circular dichroism (ICD)[10] due to interactions between the chiral G-quadruplexes and achiral PIPER. Interestingly, different ICD bands were observed in the presence of quadruplexes (T**L**G GGG)$_4$ or (GGG G**L**T)$_4$ which are believed to form dimers via tail-to-tail and head-to-head stacking of the terminal G-tetrads, respectively. This suggests a different binding mode of PIPER to the different DNA secondary structures. The results are compatible with EPR-based distance measurements that indicate intercalation of PIPER between the two monomers in the tail-to-tail stacked dimer (Figure S36). On the other hand, no intercalation can be detected into head-to-head stacked dimers (Figure S37) and PIPER binds presumably via groove binding.

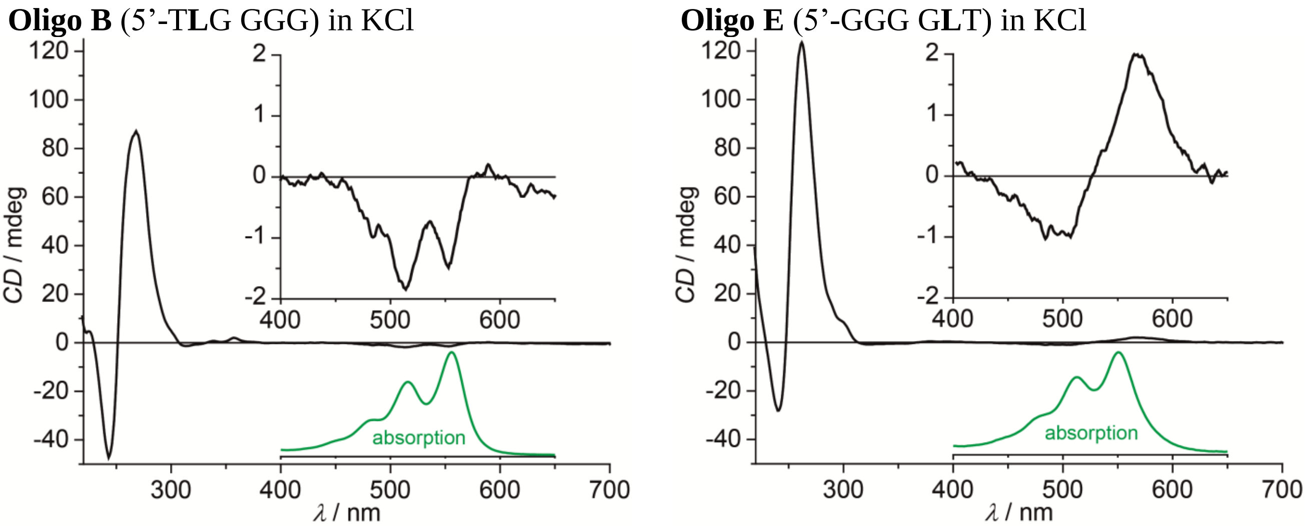

Fig. S24: CD spectra of folded G-quadruplex (T**L**G GGG)$_4$ (left) and (GGG G**L**T)$_4$ (right) in presence of 1 equiv. of $CuSO_4$ and 0.5 equiv. of PIPER. For comparison, the absorption bands of PIPER in the visible region are shown (green, from chapter 3.3).

5 EPR and PDEPR Spectroscopy

5.1 Sample Preparation

Unless stated otherwise, for all standard EPR measurements, solutions containing 1 mM single-stranded DNA, 50 mM potassium phosphate buffer pH 7.0, 375 µM $CuSO_4$ and, if present, various concentrations of PIPER, telomestatin, guanine, 7-deazaguanine, guanosine or guanosine monophosphate (GMP) were prepared. For all experiments, ultrapure water (type I, 18.2 MΩ cm) was used, obtained with a *VWR Puranity TU 3 UV*. Telomestatin was added as a 1 mM stock solution in DMSO, guanine and 7-deazaguanine were added as 20 mM stock solutions in 100 mM KOH and guanosine was added as 20 mM suspension in water.

Samples were heated to 85 °C for 10 min, slowly cooled to 4 °C with a cooling rate of 0.5 °C/min and then left at this temperature for several hours (typically overnight). To ensure full formation of G-quadruplexes, the samples were frozen at –20 °C for 1 h[7] and thawed again to 4 °C. The sample was diluted with glycerol (1:1 v/v), mixed, immediately frozen in liquid N_2.

5.2 Spectrometer and Methods

Q-Band pulse EPR measurements were carried out in the temperature range of 19 to 30 K using a *Bruker Elexsys E580* spectrometer equipped with a *Bruker ER 5106QT-2* resonator, *Bruker SpinJet AWG*, *Oxford Instruments CF935* continuous-flow helium cryostat and *Oxford Instruments MercuryiTC* temperature controller. Field-swept EPR spectra were obtained via integration of the electron spin echo (ESE) signal and simulated using the EasySpin toolbox[11] for MATLAB®. DEER experiments were performed at 19 K with the frequency separation of $\Delta f = f_{\mathrm{pump}} - f_{\mathrm{obs}}$ = 90 MHz using an overcoupled resonator, with f_{pump} set to the center of the resonator dip. Throughout the text, g_{eff} indicates the observer position. Gaussian pulses were used in order to minimize the pump and observer overlap and suppress the "2+1" artifact.[12] The optimal π-pulse lengths were determined using transient nutation experiments and were typically ~30 ns for the pump pulse and ~80 ns for the detection. The shot repetition time (SRT) was 500 µs. DEER time traces were background-corrected using DeerAnalysis 2018[13] either assuming an exponential background with dimensionality equal to 3 or by using an empirical polynomial fitting. RIDME experiments were performed at 30 K (T_{mix} = 15 µs, SRT = 300 µs) using a critically-coupled resonator with a Gaussian π-pulse length of ~30 ns. RIDME time traces were background-corrected using an empirical third-order polynomial fitting. Dipolar spectra were obtained from DEER and RIDME time traces using DeerAnalysis 2018. Distance distributions were derived from DEER time traces using PeldorFit 2019,[14] with traces' individual contributions to the overall RMSD normalized to their respective modulation depth values. RMSD heatmaps were plotted using the Python scripts supplied as part of the PeldorFit package.

5.3 EPR/DEER of Cu^{2+}-Based Spin Labels

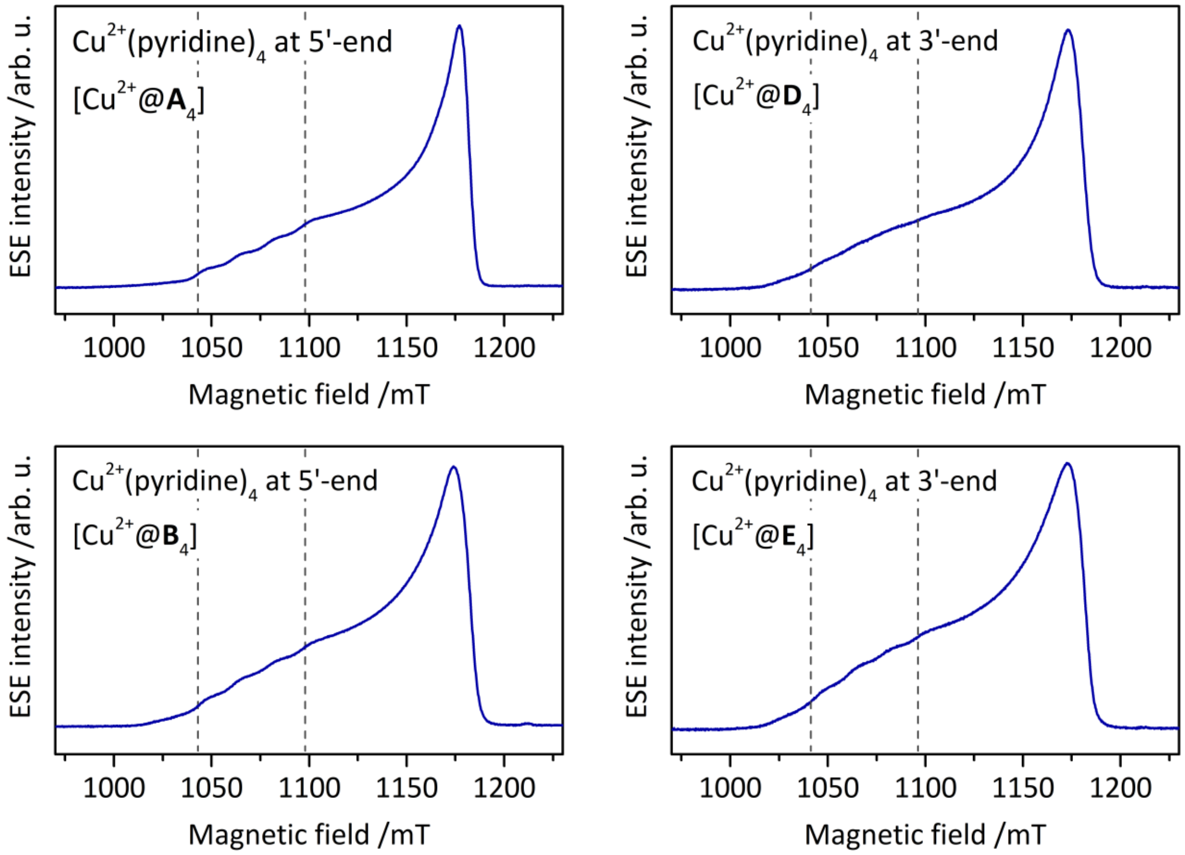

Fig. S25: Comparison of ESE-detected field-swept EPR spectra of the four G-quadruplex samples, with the Cu^{2+}-based spin label attached at the 5'-end for [Cu^{2+}@**A**$_4$] and [Cu^{2+}@**B**$_4$] and 3'-end for [Cu^{2+}@**D**$_4$] and [Cu^{2+}@**E**$_4$]. Cu^{2+} hyperfine structure in the $g_{\parallel}$ region is less pronounced for samples with the spin label attached at the 3'-end (dashed vertical lines mark the first and fourth lines of the hyperfine structure). Experimental parameters: t_π = 20 ns (rectangular pulses), τ = 300 ns, SRT = 1.2 ms, T = 19 K.

No distortion in the EPR line shape of the Cu^{2+}(pyridine)$_4$ spin label within [Cu^{2+}@**A**$_4$] was detected upon the addition of excess Cu^{2+} at 1.5 equiv. per G-quadruplex monomer (Figure S26). As the Cu^{2+} concentration was increased from 0.5 equiv. per monomer to 1.5 equiv., the EPR intensity doubled. Further increase in the Cu^{2+} concentration did not lead to a detectable increase in the EPR intensity of the spin label. However, at the Cu^{2+} concentration of 3 equiv. per monomer or higher, the spin label's EPR spectrum started to show distortions that could be attributed to the presence of free or non-specifically bound Cu^{2+}. Bottom trace in Figure S26 was recorded for a reference sample with free Cu^{2+} in phosphate buffer without DNA, which demonstrated that free Cu^{2+} in the buffer is mostly EPR silent. Thus, the Cu^{2+} concentration of 1.5 equiv. per G-quadruplex was used throughout this work, as it yields the highest EPR intensity of the spin label without introducing significant distortions into its EPR line shape.

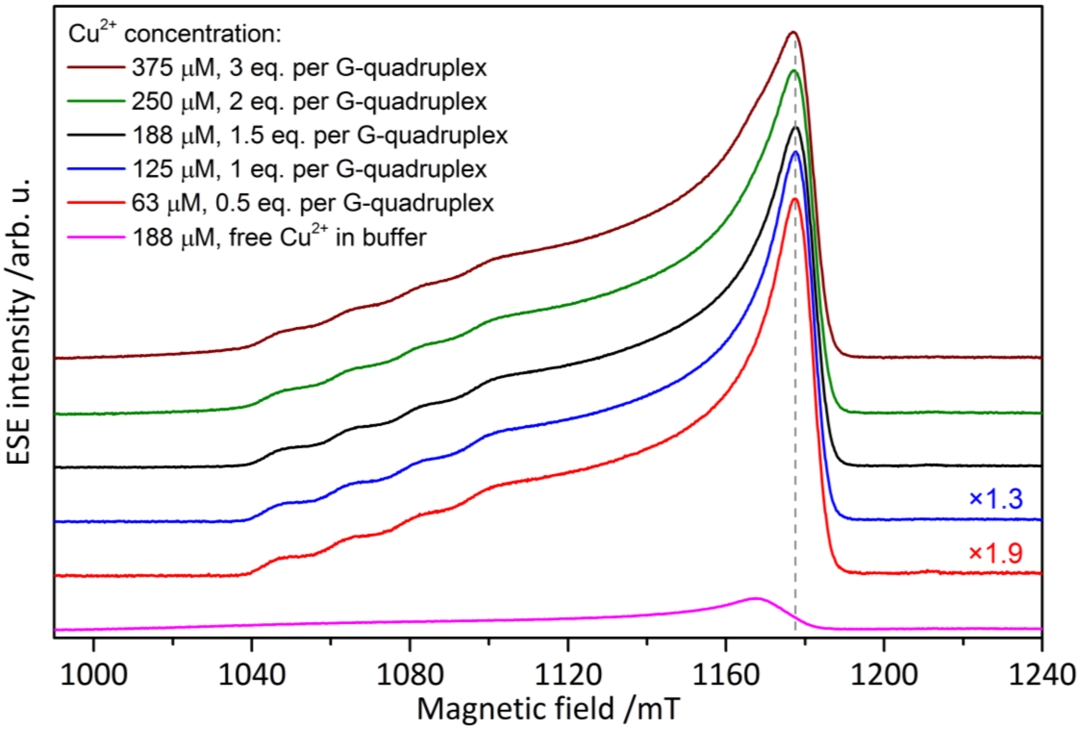

Fig. S26: Comparison of ESE-detected field-swept EPR spectra of $[Cu^{2+}@\mathbf{A}_4]_2$ prepared with various Cu^{2+} concentrations (top five traces) and spectrum of free Cu^{2+} solution in buffer (bottom trace). Two of the traces were normalized to the EPR intensity of the G-quadruplex sample with the Cu^{2+} concentration of 1.5 equiv. per monomer. G-quadruplex monomer concentration was 125 μM. Experimental parameters: t_π = 20 ns (rectangular pulses), τ = 300 ns, SRT = 1.2 ms, T = 19 K.

To test if the excess copper influences DEER modulation depths (Δ), DEER time traces were measured for $[Cu^{2+}@\mathbf{A}_4]$ G-quadruplex samples containing 1.5, 2, 3 and 5 equiv. of Cu^{2+} per monomer. Modulation depth (observer position g_{eff} = 2.061) as a function of Cu^{2+} concentration is shown in Fig. S27. Considering batch-to-batch variation in Δ for nominally identical samples, the DEER modulation depth stays virtually constant between 1.5 and 3 equiv. of Cu^{2+}. At 5 equiv. of Cu^{2+} per monomer, the modulation depth appears to marginally decrease. Thus, at the Cu^{2+} concentration of 1.5 equiv. per monomer used throughout this work, the excess copper does not affect the observed DEER modulation depths.

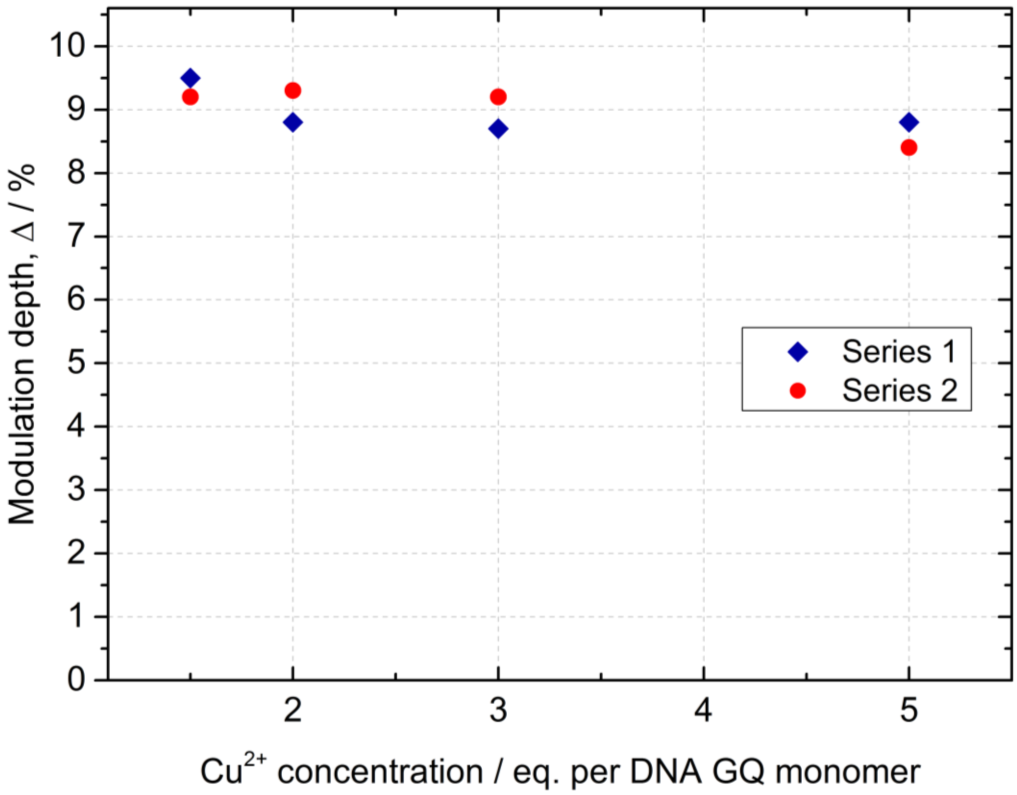

Fig. S27: DEER modulation depth (observer position g_{eff} = 2.061) as a function of Cu^{2+} concentration for two series of [Cu^{2+}@**A**$_4$]$_2$ samples. G-quadruplex monomer concentration was 125 µM.

Primary DEER data (black) measured at multiple field positions for spin-labeled G-quadruplex samples used in the present work, overlaid with the background fits (red), are shown in Fig. S28. DEER time traces measured at the observer position g_{eff} = 2.121 (traces *c* in Fig. S28 A, F, G, I, J and K) demonstrate a nearly complete dampening of dipolar modulations.

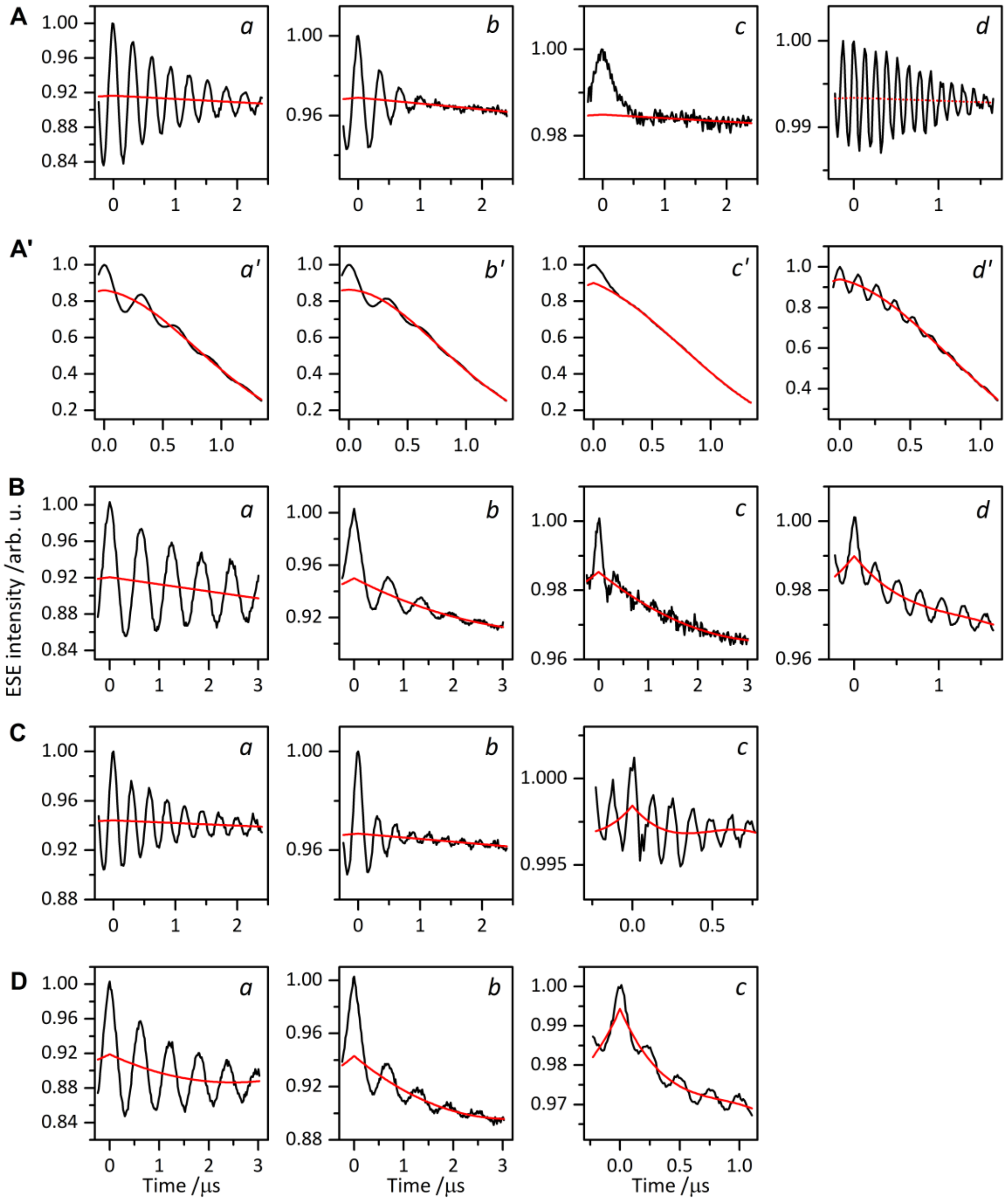

Fig. S28: Primary orientation-selective DEER (RIDME in **A'**) traces (black) overlaid with the background fits (red) for several observer positions denoted by g_{eff}. (**A**) [Cu^{2+}@$\mathbf{A}_4$]$_2$: g_{eff} = 2.061 (*a*), 2.071 (*b*), 2.121 (*c*), 2.315 (*d*); (**A'**) same as A for RIDME; **(B)** [Cu^{2+}@$\mathbf{B}_4$]$_2$: g_{eff} = 2.061 (*a*), 2.071 (*b*), 2.225 (*c*), 2.315 (*d*); (**C**) [Cu^{2+}@$\mathbf{D}_4$]$_2$: g_{eff} = 2.061 (*a*), 2.071 (*b*), 2.315 (*c*); (**D**) [Cu^{2+}@$\mathbf{E}_4$]$_2$: g_{eff} = 2.061 (*a*), 2.071 (*b*), 2.315 (*c*);

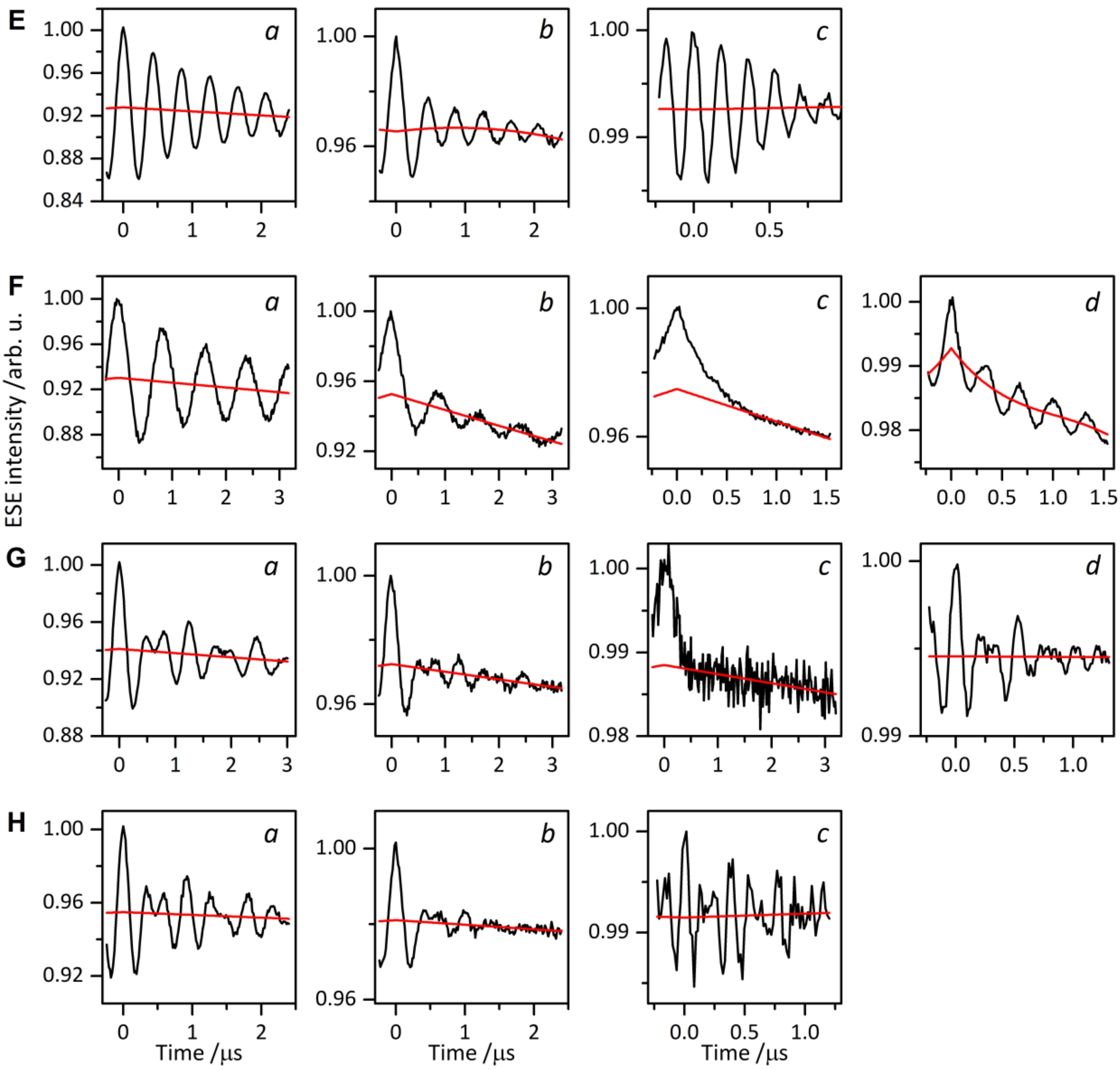

Fig. S28 (cont.): Primary orientation-selective DEER traces (black) overlaid with the background fits (red) for several observer positions denoted by g_{eff}. (**E**) [Cu^{2+}@**A**$_4$]$_2$ with PIPER (1 equiv. per dimer): g_{eff} = 2.061 (*a*), 2.071 (*b*), 2.315 (*c*); (**F**) [Cu^{2+}@**B**$_4$]$_2$ with PIPER (1 equiv. per dimer): g_{eff} = 2.061 (*a*), 2.071 (*b*), 2.121 (*c*), 2.315 (*d*); (**G**) [Cu^{2+}@**A**$_4$]$_2$ with PIPER (2 equiv. per dimer): g_{eff} = 2.061 (*a*), 2.071 (*b*), 2.121 (*c*), 2.315 (*d*); (**H**) [Cu^{2+}@**A**$_4$]$_2$ with telomestatin (1 equiv. per dimer): g_{eff} = 2.061 (*a*), 2.071 (*b*), 2.315 (*c*);

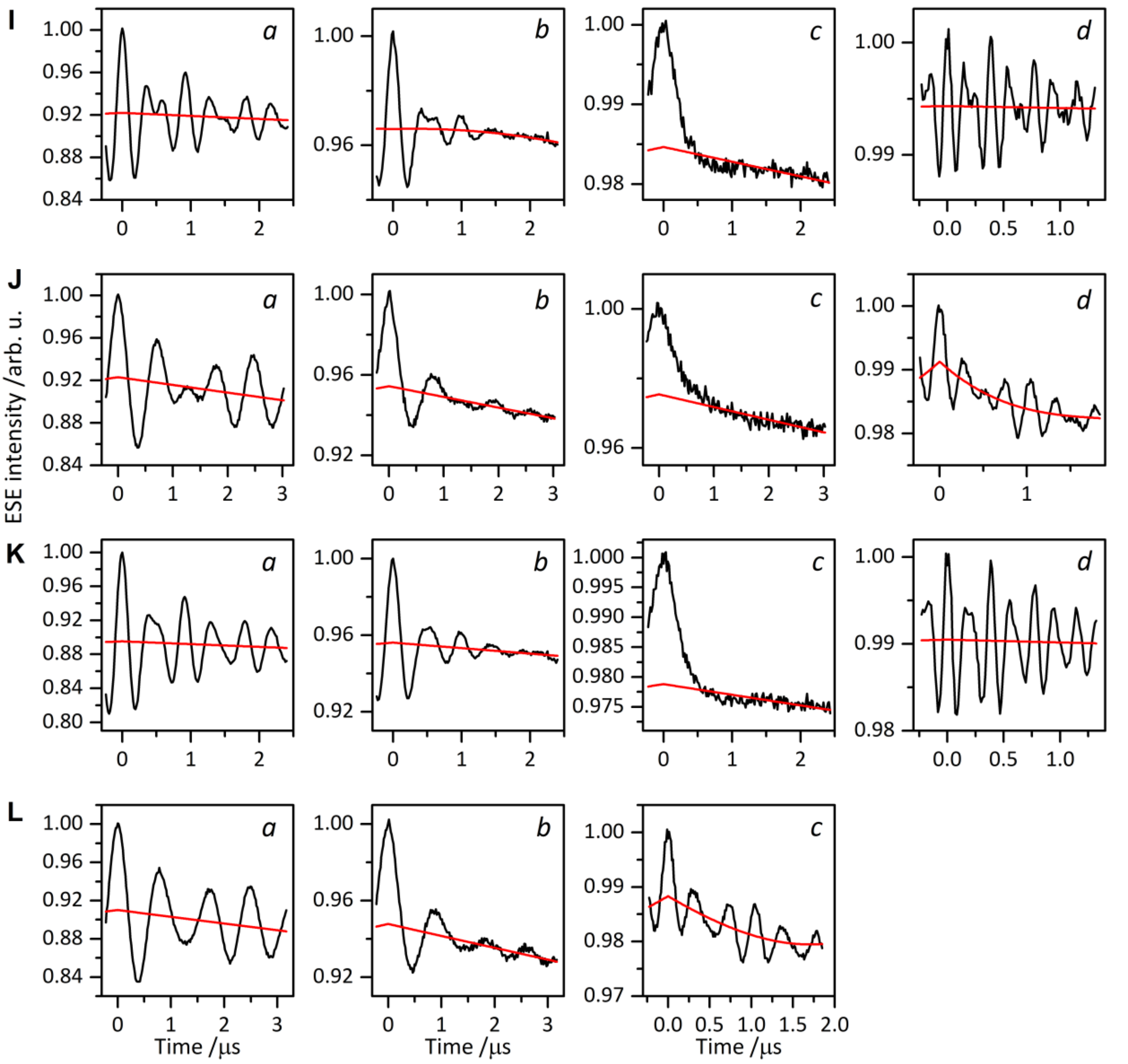

Fig. S28 (cont.): Primary orientation-selective DEER traces (black) overlaid with the background fits (red) for several observer positions denoted by g_{eff}. (**I**) [Cu^{2+}@**A**$_4$]$_2$ with guanine (4 equiv. per dimer): g_{eff} = 2.061 (*a*), 2.071 (*b*), 2.121 (*c*), 2.315 (*d*); (**J**) [Cu^{2+}@**B**$_4$]$_2$ with guanine (4 equiv. per dimer): g_{eff} = 2.061 (*a*), 2.071 (*b*), 2.121 (*c*), 2.315 (*d*); (**K**) [Cu^{2+}@**A**$_4$]$_2$ with guanosine (4 equiv. per dimer): g_{eff} = 2.061 (*a*), 2.071 (*b*), 2.121 (*c*), 2.315 (*d*); (**L**) [Cu^{2+}@**B**$_4$]$_2$ with guanosine (4 equiv. per dimer): g_{eff} = 2.061 (*a*), 2.071 (*b*), 2.315 (*c*);

5.4 Comparison of Orientation-Selective DEER and RIDME Datasets

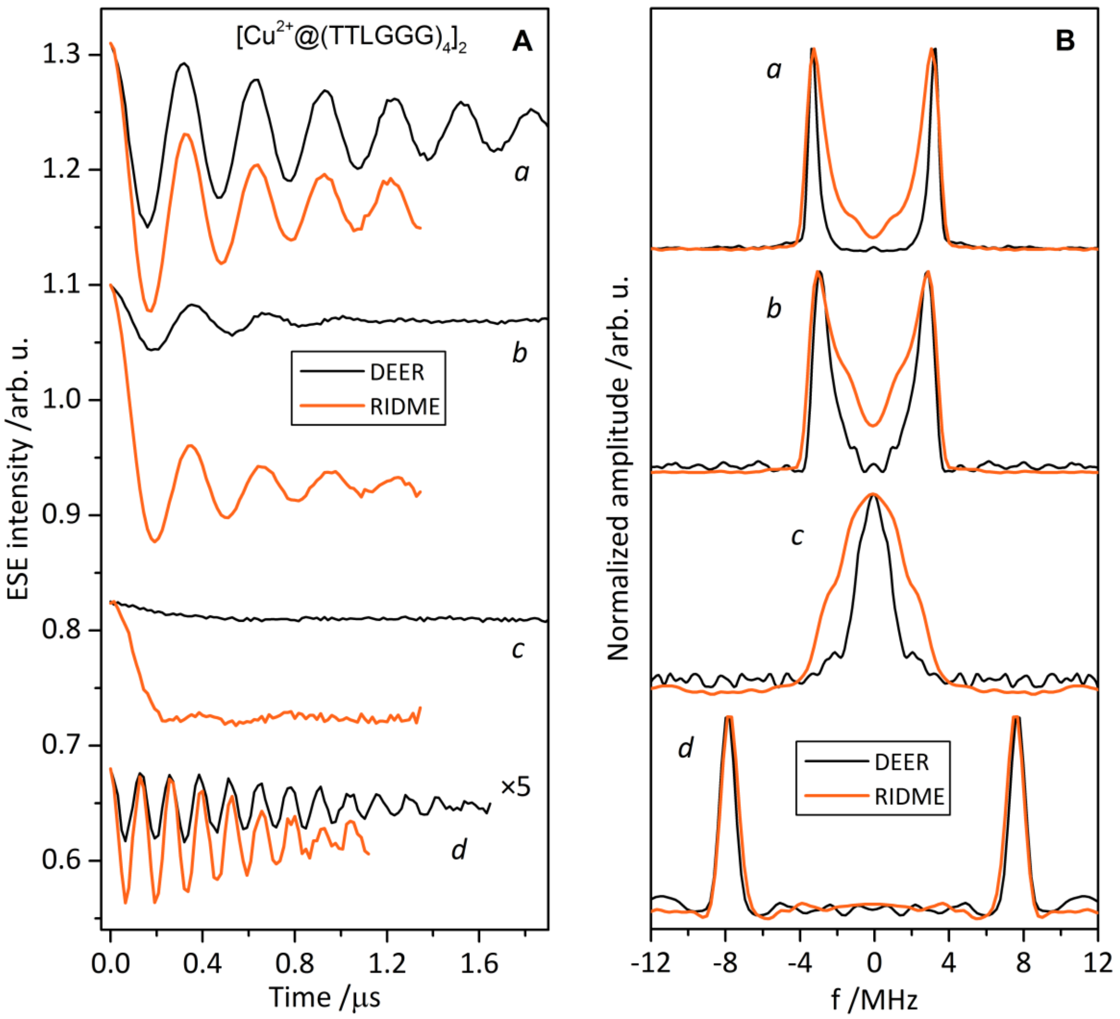

Fig. S29: (**A**) Background-corrected orientation-selective DEER (black lines) and RIDME (orange lines) time traces of $[Cu^{2+}@\mathbf{A}_4]_2$ measured at four field positions. Observer positions are marked with *a*-*d* and correspond to g_{eff} = 2.061, 2.071, 2.121 and 2.315, respectively; (**B**) Dipolar spectra obtained from the corresponding DEER (black lines) and RIDME (orange lines) time traces.

5.5 Orientation-Selective DEER Data

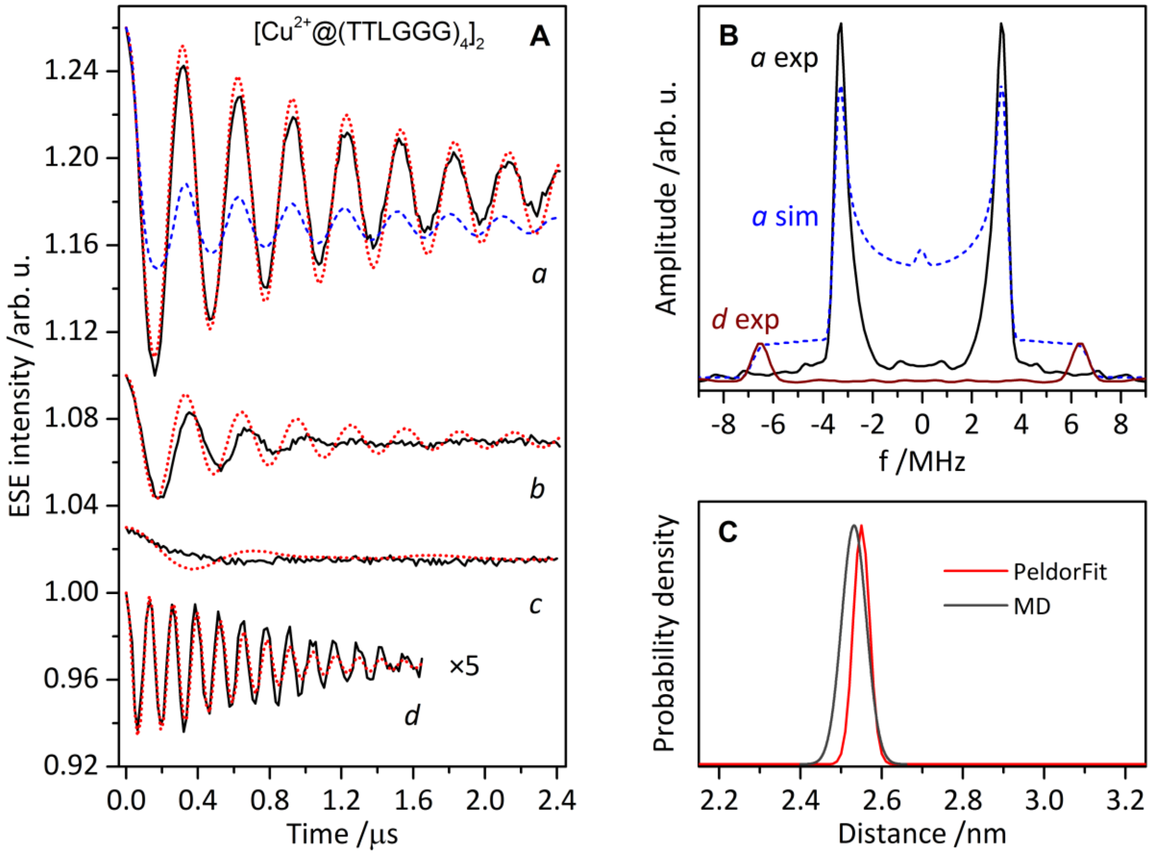

Fig. S30: (**A**) Background-corrected orientation-selective DEER time traces of $[Cu^{2+}@\mathbf{A}_4]_2$ measured at four field positions (black solid lines) overlaid with the best fit results from PeldorFit (red dotted lines) and DeerAnalysis (blue dashed line). Observer positions are marked with *a*-*d* and correspond to g_{eff} = 2.061, 2.071, 2.121 and 2.315, respectively; Trace *a* corresponds to the $g_\perp$ region and trace *d* to $g_\parallel$. (**B**) Dipolar spectra detected at positions *a* (black solid line) and *d* (frequency axis scaled by $g_\perp^2/g_\parallel^2$, amplitude normalized to the Pake pattern intensity, dark red solid line) overlaid with a Pake pattern simulated by DeerAnalysis based on time trace *a* (blue dashed line); (**C**) Distance distributions obtained from experiment using PeldorFit (red solid line) and from MD simulations (grey solid line).

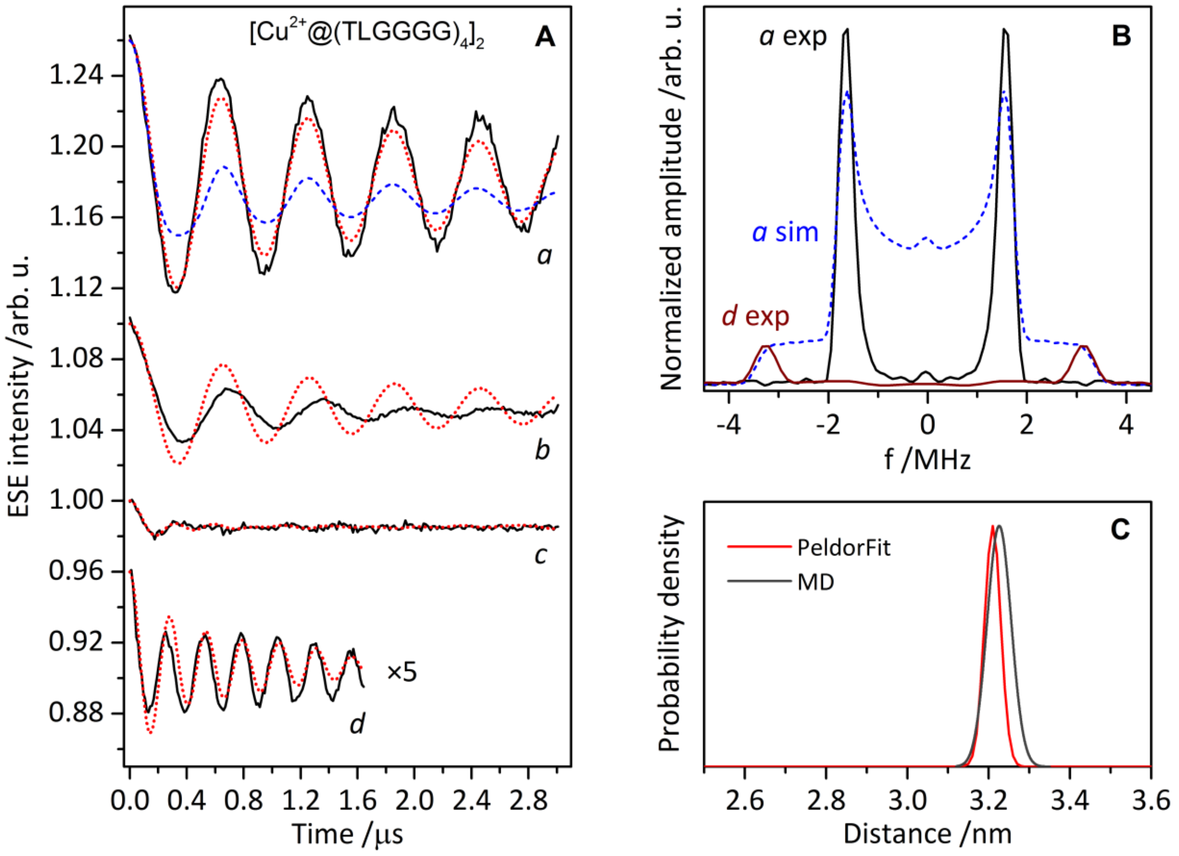

Fig. S31: (**A**) Background-corrected orientation-selective DEER time traces of $[Cu^{2+}@\mathbf{B}_4]_2$ measured at four field positions (black solid lines) overlaid with the best fit results from PeldorFit (red dotted lines) and DeerAnalysis (blue dashed line). Observer positions are marked with *a*-*d* and correspond to g_{eff} = 2.061, 2.071, 2.225 and 2.315, respectively; Trace *a* corresponds to the $g_{\perp}$ region and trace *d* to $g_{\parallel}$. (**B**) Dipolar spectra detected at positions *a* (black solid line) and *d* (frequency axis scaled by $g_{\perp}^2/g_{\parallel}^2$, amplitude normalized to the Pake pattern intensity, dark red solid line) overlaid with a Pake pattern simulated by DeerAnalysis based on time trace *a* (blue dashed line); (**C**) Distance distributions obtained from experiment using PeldorFit (red solid line) and from MD simulations (grey solid line).

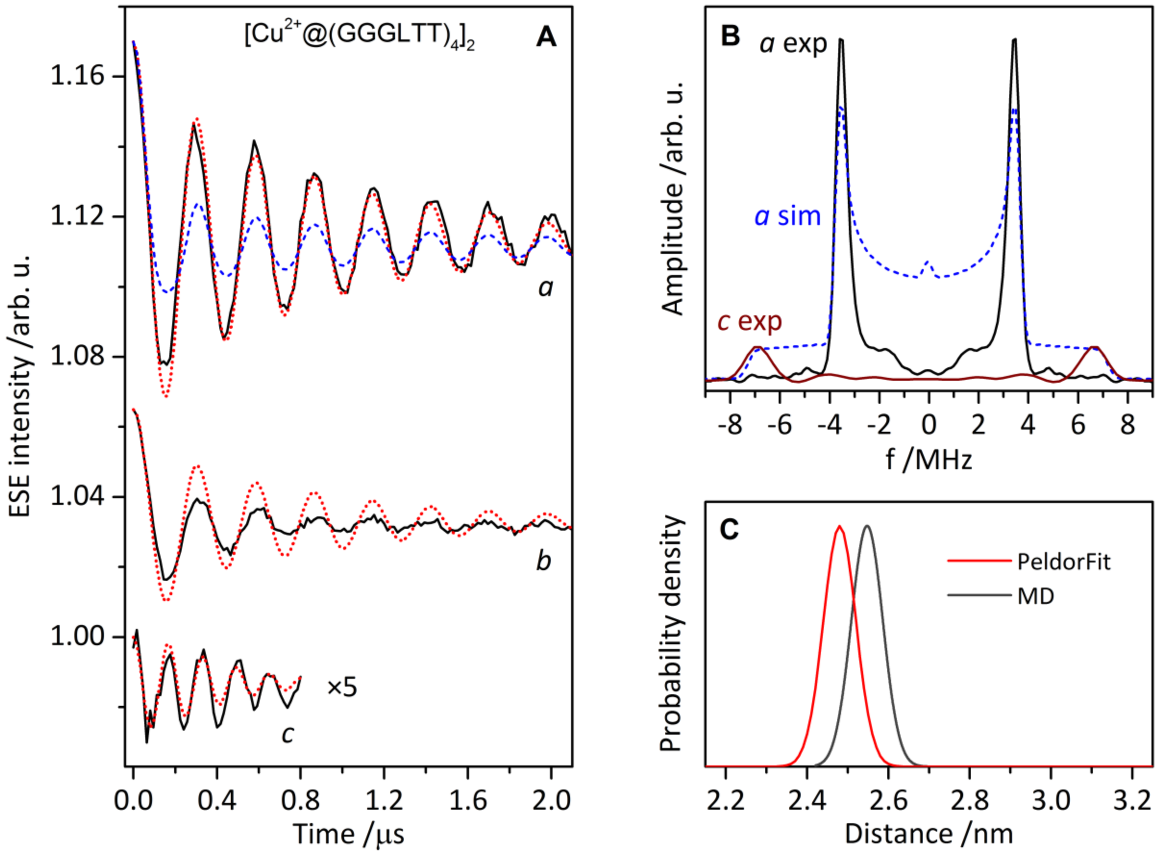

Fig. S32: (**A**) Background-corrected orientation-selective DEER time traces of [Cu^{2+}@$\mathbf{D}_4$]$_2$ measured at three field positions (black solid lines) overlaid with the best fit results from PeldorFit (red dotted lines) and DeerAnalysis (blue dashed line). Observer positions are marked with *a*-*c* and correspond to g_{eff} = 2.061, 2.071, and 2.315, respectively; Trace *a* corresponds to the $g_\perp$ region and trace *c* to $g_\parallel$. (**B**) Dipolar spectra detected at positions *a* (black solid line) and *c* (frequency axis scaled by $g_\perp^2/g_\parallel^2$, amplitude normalized to the Pake pattern intensity, dark red solid line) overlaid with a Pake pattern simulated by DeerAnalysis based on time trace *a* (blue dashed line); (**C**) Distance distributions obtained from experiment using PeldorFit (red solid line) and from MD simulations (grey solid line).

The sample containing G-quadruplex dimer [Cu^{2+}@$\mathbf{D}_4$]$_2$ shows the lowest modulation depth of all samples indicating only a smaller fraction of G-quadruplexes formed dimers. This observation is consistent with the biphasic UV-based denaturation profiles found for samples containing oligo **D** (Figure S13). A different species (other than the usual parallel tetramolecular G-quadruplex) might be present, which is unable to form dimers.

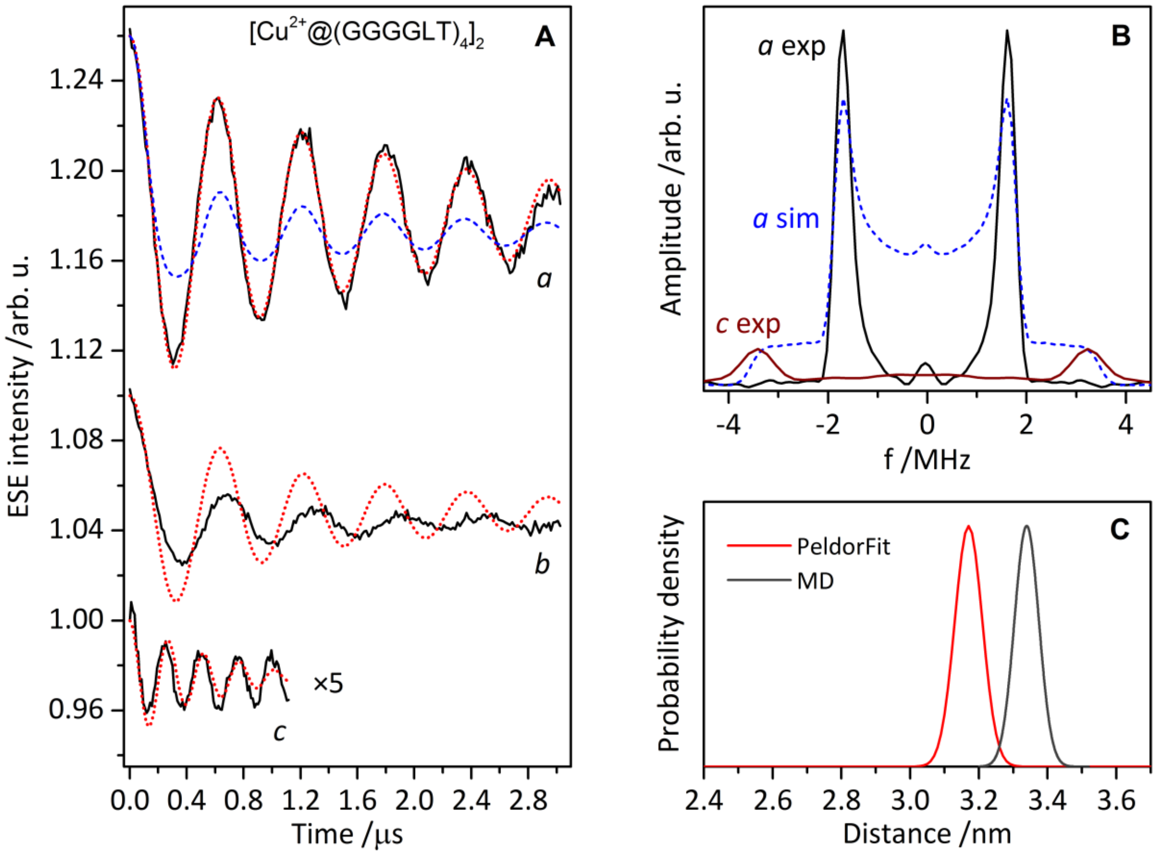

Fig. S33: (**A**) Background-corrected orientation-selective DEER time traces of $[Cu^{2+}@\mathbf{E}_4]_2$ measured at three field positions (black solid lines) overlaid with the best fit results from PeldorFit (red dotted lines) and DeerAnalysis (blue dashed line). Observer positions are marked with *a*-*c* and correspond to g_{eff} = 2.061, 2.071, and 2.315, respectively; Trace *a* corresponds to the $g_\perp$ region and trace *c* to $g_\parallel$. (**B**) Dipolar spectra detected at positions *a* (black solid line) and *c* (frequency axis scaled by $g_\perp^2/g_\parallel^2$, amplitude normalized to the Pake pattern intensity, dark red solid line) overlaid with a Pake pattern simulated by DeerAnalysis based on time trace *a* (blue dashed line); (**C**) Distance distributions obtained from experiment using PeldorFit (red solid line) and from MD simulations (grey solid line).

5.6 Structural Details of Dimers Obtained from PeldorFit Simulations

PeldorFit simulations of the orientation-selective DEER data provide information not only on the Cu-Cu distances, but also on the inter-spin vector orientation, as well as relative orientation of the two *g*-frames.[14] A geometrical model of a G-quadruplex dimer, analogous to that used for a doubly labeled monomer,[15] is shown in Fig. S34. Relevant angles used in the model are ξ, polar angle of the inter-spin vector, and β, tilting angle of the two equatorial planes of the Cu^{2+}(pyridine)$_4$ complexes.

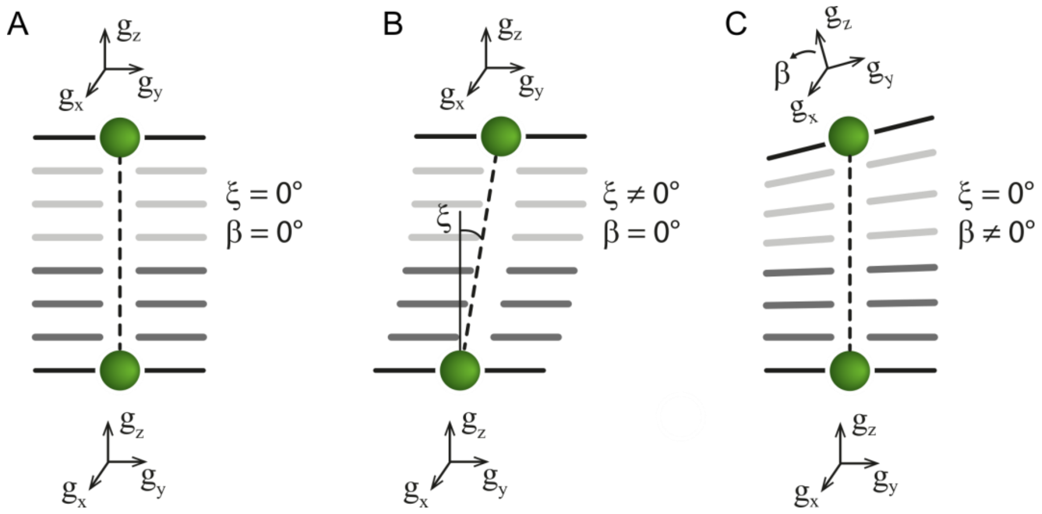

Fig. S34: Schematic representation of a π-stacked G-quadruplex dimer showing structural details affecting the EPR distance measurements. Green spheres: Cu^{2+} ions; light and dark grey bars: G-tetrads; black bars: Cu^{2+} coordination planes. (**A**) Coplanar G-quartet stacking. The inter-spin vector (dashed black line) is collinear with the g_z-axis of the observer spin, and the g-frames of the two Cu^{2+} ions are collinear. (**B**) Deviation from collinearity between the observer spin g_z axis and inter-spin vector. (**C**) Tilting of the plane of (at least) one Cu^{2+} complex.[15]

Mean values and gaussian distribution widths of ξ, β and Cu-Cu distance *d* for the various dimeric species as determined by the PeldorFit simulations are listed in Table S2. While the listed values were found to produce the best fit to the experimental traces, the uncertainties in both mean values and distribution widths can be estimated from the RMSD plots shown in Fig. S35 (see ref [14]). The uncertainties in mean values $\langle d \rangle$ and distribution widths Δd of Cu-Cu distances were estimated as ranges in which the RMSD reaches 110% if its minimal value[14] (columns 3 and 4 of Table S2).

As seen in the RMSD plots for the ξ angle (second column in Figure S35), its mean values generally tend to be close to zero for both pure DNA dimers and sandwich-type complexes with intercalating ligands. Its distribution width, Δξ, as well as the uncertainty range of Δξ varies significantly for different dimeric species (e.g., compare the plots for $[Cu^{2+}@\mathbf{A}_4]_2$ and $[Cu^{2+}@\mathbf{E}_4]_2$ shown in Fig. S35 A and D, respectively). Mean values of β show a larger variation based on the type of dimeric species than those of ξ. They do not, however, exceed 20 degree. We note that a proper angle analysis requires a two-dimensional DEER dataset, rather than discrete measurements at several field positions. However, despite the uncertainties in the mean values and distribution widths of ξ and β, the obtained Cu–Cu distances are well-defined and show extremely narrow distributions for both pure DNA dimers and sandwich-type complexes (see the first column in Figure S35). These results demonstrate that all the samples under investigation in this work show highly rigid nature, the $[Cu^{2+}@\mathbf{A}_4]_2$ dimer being the most rigid one.

Tab. S2: Cu^{2+}–Cu^{2+} distances derived from DEER experiments using PeldorFit and those obtained from MD simulations for the various dimeric species formed by G-quadruplexes with and without intercalating ligands. Angles used in PeldorFit simulations are given in the last two columns.

G-quadruplex dimer	⟨*d*⟩ (Δ*d*) /nm, PeldorFit[a]	⟨*d*⟩, nm uncertainty range[b]	Δ*d*, nm uncertainty range[b]	⟨*d*⟩ (Δ*d*) /nm, MD simulations	⟨ξ⟩ (Δξ) /deg [a,c]	⟨β⟩ (Δβ) /deg [a,c]
$[Cu^{2+}@\mathbf{A}_4]_2$	2.55 (0.02)	2.54-2.55	0.02-0.02	2.53 (0.03)	0 (1)	0 (1)
$[Cu^{2+}@\mathbf{B}_4]_2$	3.21 (0.02)	3.19-3.23	0.01-0.03	3.23 (0.03)	8 (19)	4 (2)
$[Cu^{2+}@\mathbf{D}_4]_2$	2.48 (0.04)	2.47-2.49	0.04-0.06	2.55 (0.04)	6 (6)	18 (8)
$[Cu^{2+}@\mathbf{E}_4]_2$	3.17 (0.04)	3.15-3.20	0.04-0.08	3.34 (0.04)	3 (14)	6 (11)
PIPER@$[Cu^{2+}@\mathbf{A}_4]_2$	2.82 (0.03)	2.81-2.83	0.03-0.05	2.84 (0.03)	4 (8)	1 (12)
PIPER@$[Cu^{2+}@\mathbf{B}_4]_2$	3.48 (0.05)	3.47-3.49	0.05-0.05	3.46 (0.05)	6 (23)	4 (3)
2PIPER@$[Cu^{2+}@\mathbf{A}_4]_2$	3.21 (0.05)	3.20-3.22	0.05-0.07	3.18 (0.03)	3 (17)	15 (14)
telomestatin@$[Cu^{2+}@\mathbf{A}_4]_2$	2.88 (0.04)	2.84-2.90	0.04-0.06	2.88 (0.04)	4 (13)	9 (27)
guanine$_4$@$[Cu^{2+}@\mathbf{A}_4]_2$	2.88 (0.03)	2.88-2.90	0.03-0.05	-	1 (0)	11 (24)
guanine$_4$@$[Cu^{2+}@\mathbf{B}_4]_2$	3.54 (0.03)	3.51-3.55	0.03-0.04	-	0 (0)	14 (25)
guanosine$_4$@$[Cu^{2+}@\mathbf{A}_4]_2$	2.88 (0.02)	2.87-2.94	0.02-0.03	2.88 (0.03)	1 (1)	6 (18)
guanosine$_4$@$[Cu^{2+}@\mathbf{B}_4]_2$	3.54 (0.01)	3.51-3.57	0.01-0.02	3.56 (0.03)	0 (6)	1 (10)

[a] Mean values with standard deviations providing the best fit to the experimental data; the uncertainties in these values can be estimated from the RMSD plots shown in Fig. S35. [b] The range in which 110% of the minimal RMSD is reached. [c] Angles used in PeldorFit simulations; ξ: polar angle of the inter-spin vector; β: tilting angle of the two equatorial planes of the complexes (see Fig. S34).

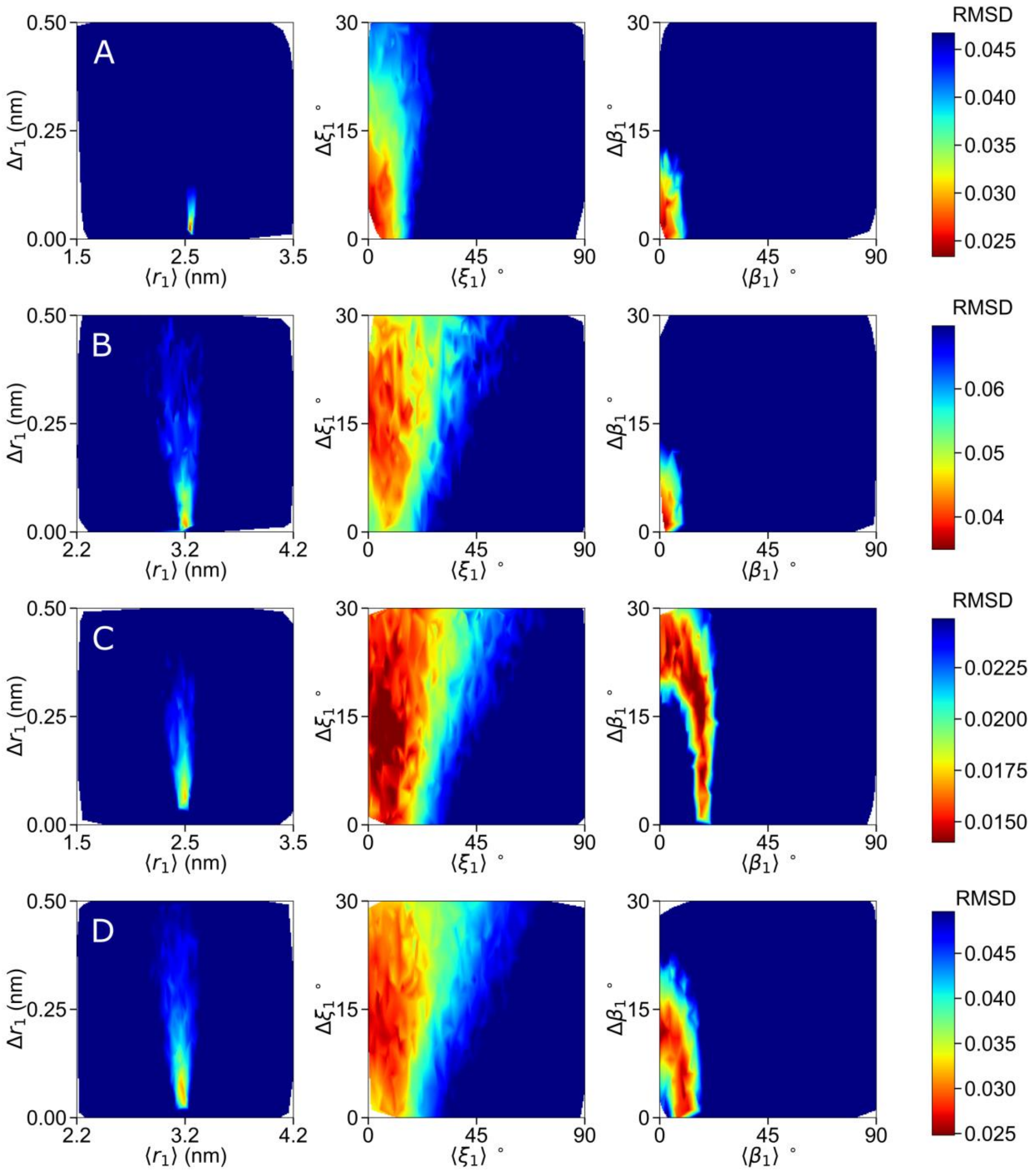

Fig. S35: RMSD surfaces for the geometric parameters (see Figure S34) used to fit the orientation-selective DEER data (see ref. [14]). Each heatmap is plotted for the mean value and distribution width of one geometric parameter, with all other parameters set to their optimized values (Table S2). (**A**) $[Cu^{2+}@\mathbf{A}_4]_2$; (**B**) $[Cu^{2+}@\mathbf{B}_4]_2$; (**C**) $[Cu^{2+}@\mathbf{D}_4]_2$; (**D**) $[Cu^{2+}@\mathbf{E}_4]_2$;

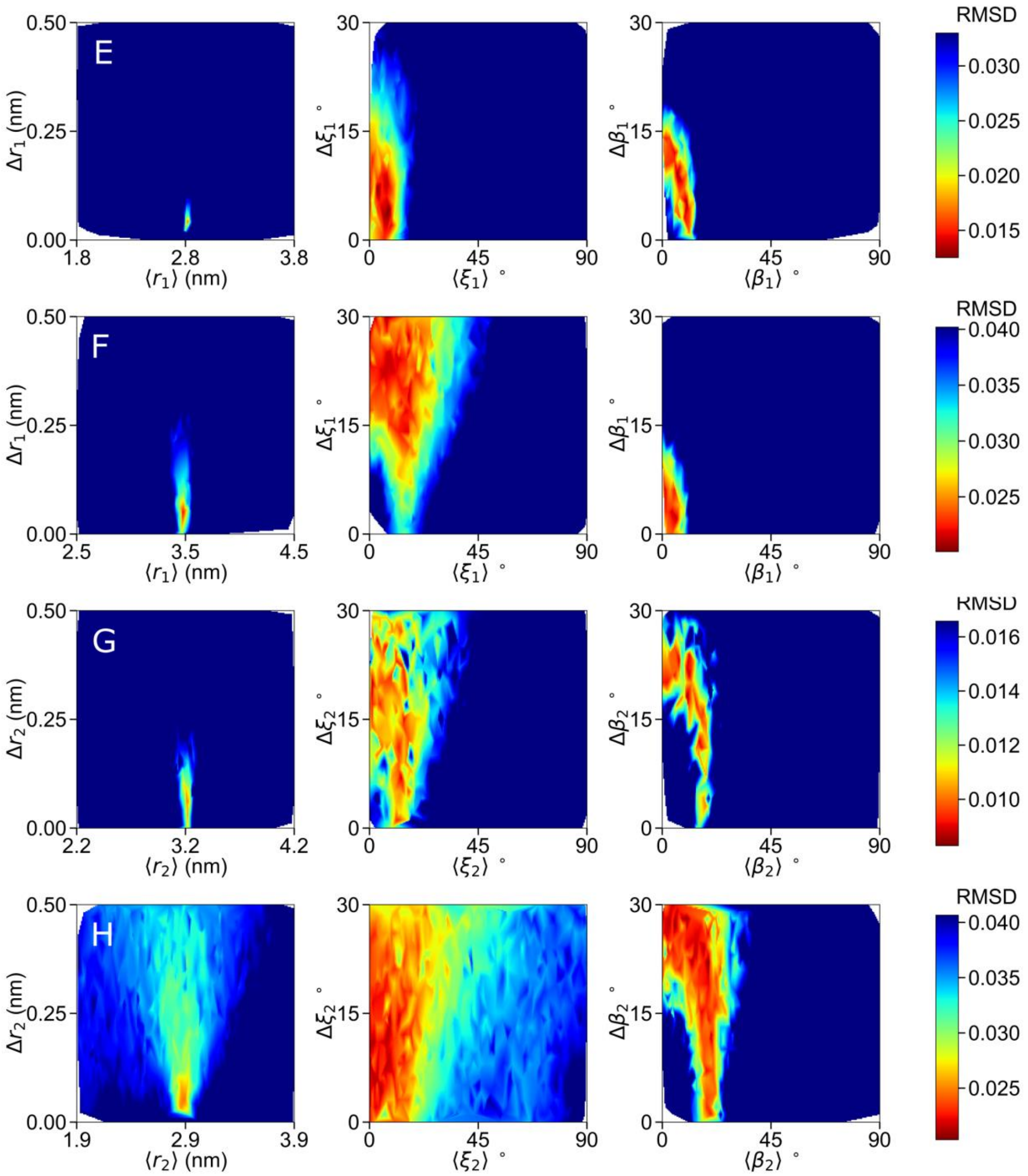

Fig. S35 (cont.): RMSD surfaces for the geometric parameters (see Figure S34) used to fit the orientation-selective DEER data (see ref. [14]). Each heatmap is plotted for the mean value and distribution width of one geometric parameter, with all other parameters set to their optimized values (Table S2). (**E**) PIPER@[Cu^{2+}@**A**$_4$]$_2$ (1 equiv. per dimer); (**F**) PIPER@[Cu^{2+}@**B**$_4$]$_2$ (1 equiv. per dimer); (**G**) 2PIPER@[Cu^{2+}@**A**$_4$]$_2$ (2 equiv. per dimer); (**H**) telomestatin@[Cu^{2+}@**A**$_4$]$_2$ (1 equiv. per dimer);

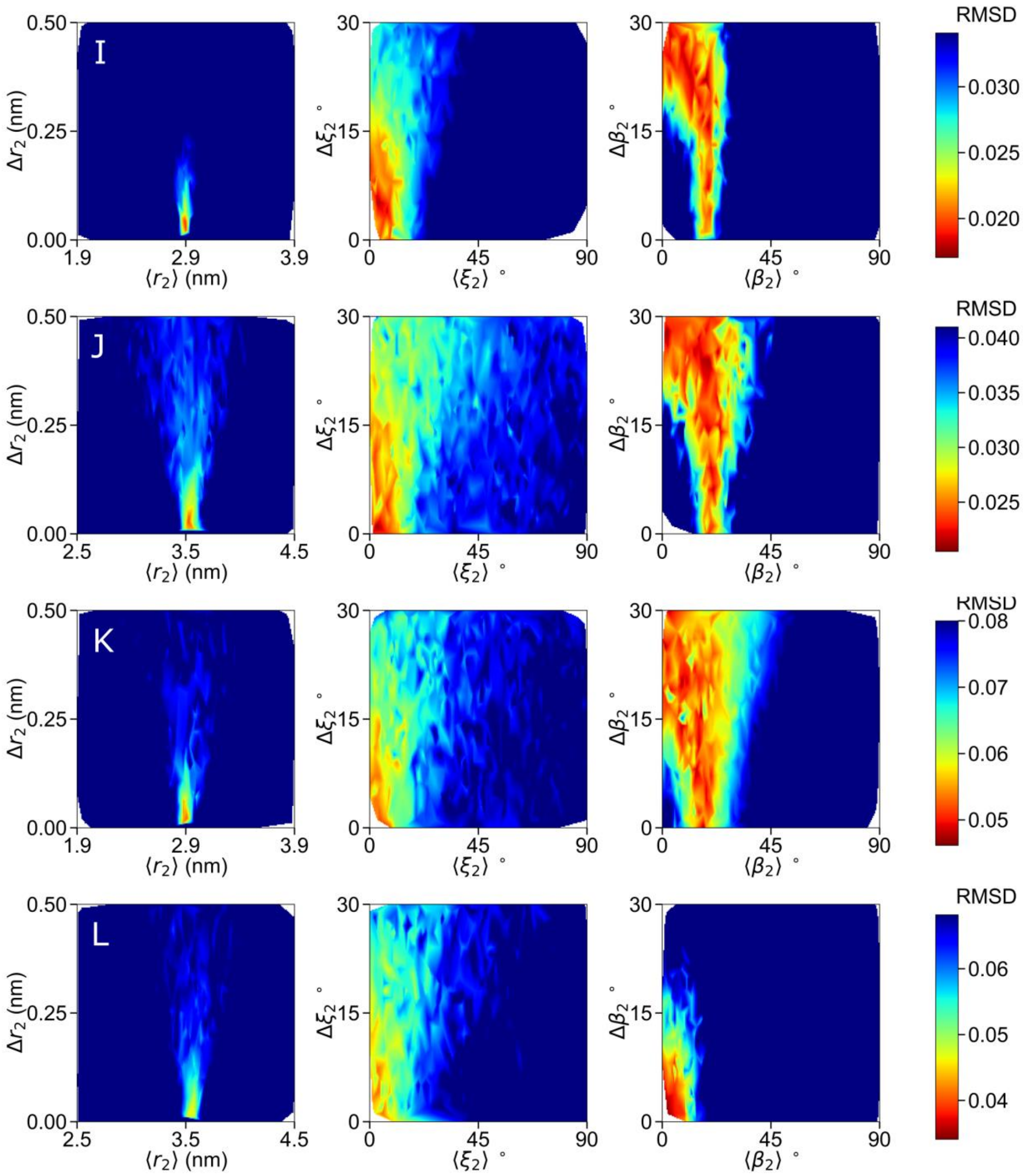

Fig. S35 (cont.): RMSD surfaces for the geometric parameters (see Figure S34) used to fit the orientation-selective DEER data (see ref. [14]). Each heatmap is plotted for the mean value and distribution width of one geometric parameter, with all other parameters set to their optimized values (Table S2). (**I**) guanine@[Cu^{2+}@**A**$_4$]$_2$ (4 equiv. per dimer); (**J**) guanine@[Cu^{2+}@**B**$_4$]$_2$ (4 equiv. per dimer); (**K**) guanosine@[Cu^{2+}@**A**$_4$]$_2$ (4 equiv. per dimer); (**L**) guanosine@[Cu^{2+}@**B**$_4$]$_2$ (4 equiv. per dimer).

5.7 Obstruction of Dimer Formation

Introducing additional thymidines next to the terminal G-quartet at either 3'-end (quadruplex [Cu^{2+}@**C**$_4$]) or 5'-end (quadruplex [Cu^{2+}@**F**$_4$]) is expected to prevent stacking of the terminal tetrads, thus blocking the dimer formation.[16] DEER experiments showed no dipolar modulation for the two modified quadruplexes, confirming that the extra 3'- or 5'-terminal thymidines prevent the dimerization.

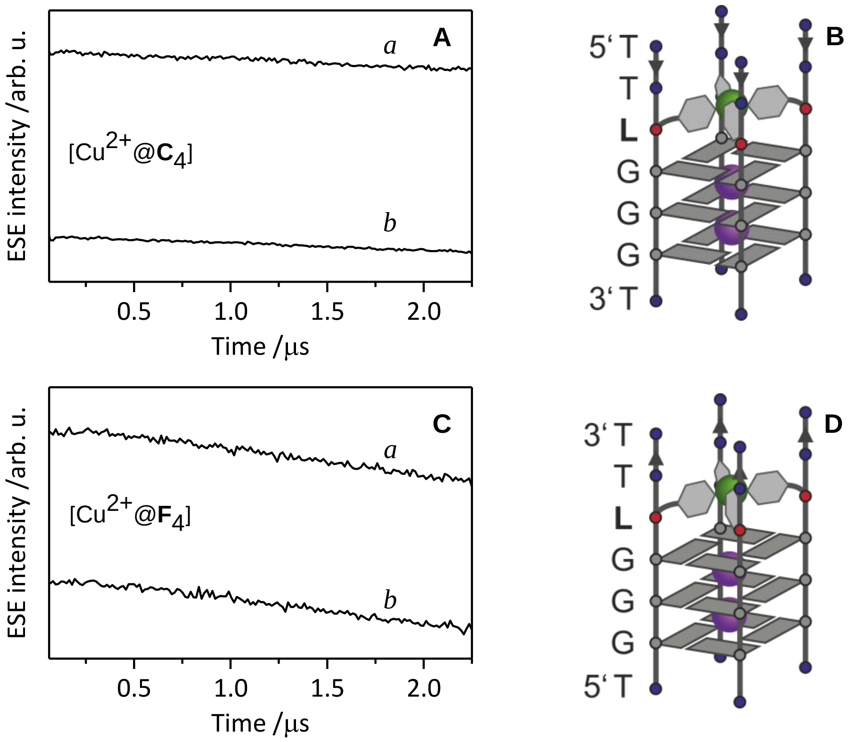

Fig. S36: (**A**) and (**C**) Primary DEER time traces measured at two field positions for [Cu^{2+}@**C**$_4$] and [Cu^{2+}@**F**$_4$] G-quadruplexes. Observer positions are marked with *a* and *b* and correspond to g_{eff} = 2.061 and 2.071, respectively; (**B**) and (**D**) Structures of the corresponding G-quadruplexes.

5.8 Intercalation of PIPER into 3'-3' Stacked Dimers

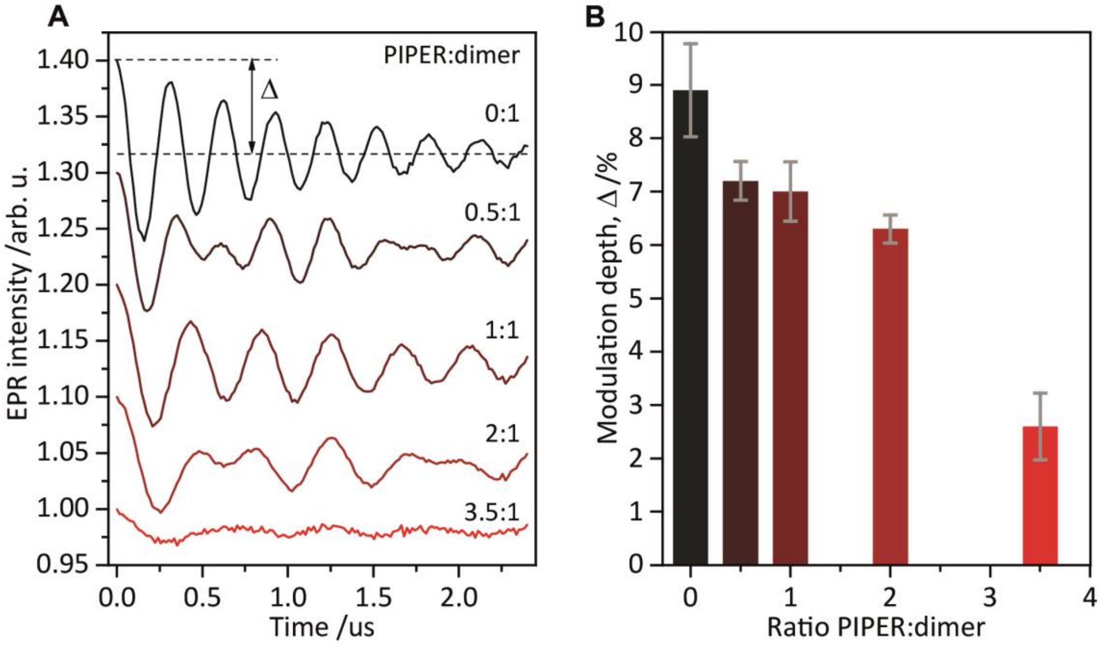

Fig. S37: (**A**) Background-corrected orientation-selective DEER time traces of [Cu^{2+}@**A**$_4$]$_2$ with varying PIPER to DNA ratios measured at the observer position g_{eff} = 2.061; (**B**) DEER modulation depths obtained for the corresponding time traces.

For ligand-containing dimeric species in our study that were only found in combination with the corresponding pure (shorter) dimers ([Cu^{2+}@**A**$_4$]$_2$ and [Cu^{2+}@**B**$_4$]$_2$ with guanine and guanosine, [Cu^{2+}@**A**$_4$]$_2$ with 2 PIPER molecules and [Cu^{2+}@**A**$_4$]$_2$ with telomestatin), the DEER data were simulated by fixing the geometric parameters of the pure (shorter) dimers and varying parameters of a new species and its relative weight. This was done using the "Model 2" mode of PeldorFit 2019. The weight parameter as determined in the simulation was then used to plot the overall distance distribution.

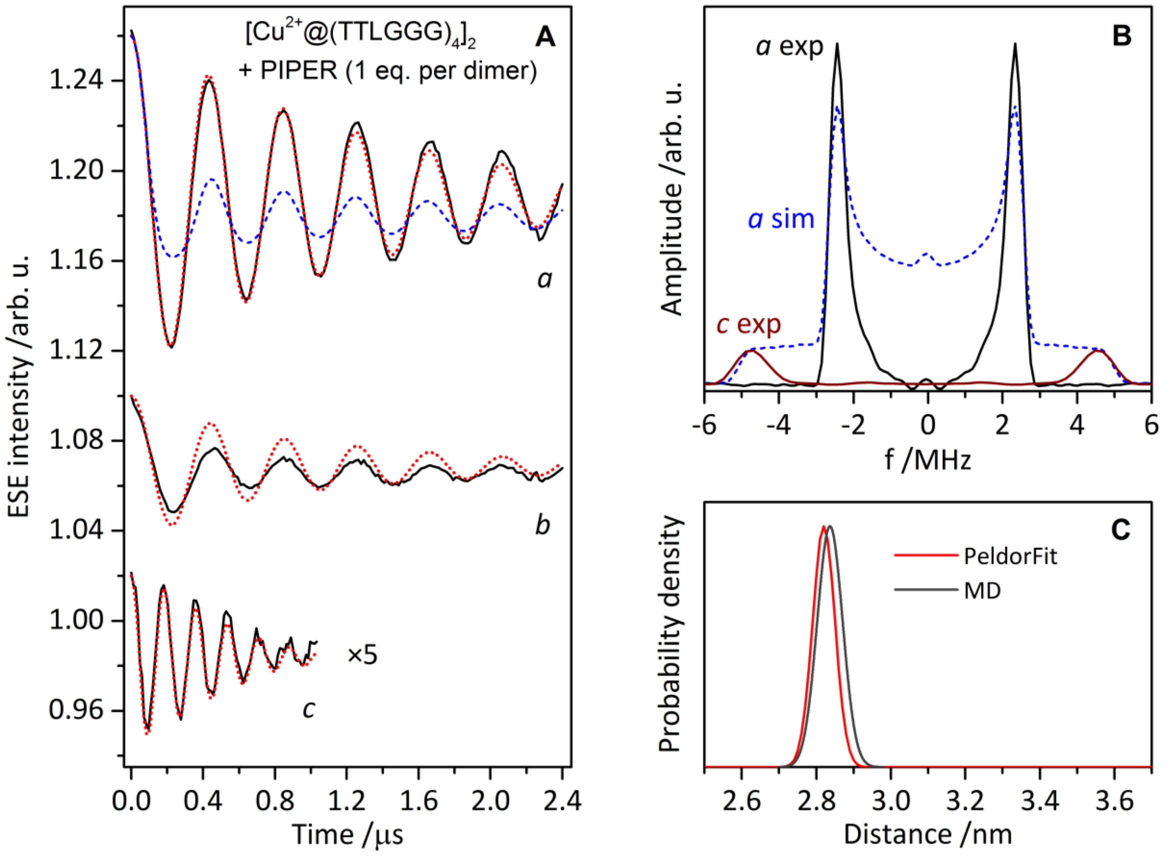

Fig. S38: (**A**) Background-corrected orientation-selective DEER time traces of $[Cu^{2+}@\mathbf{A}_4]_2$ with PIPER (1 equiv. per dimer) measured at three field positions (black solid lines) overlaid with the best fit results from PeldorFit (red dotted lines) and DeerAnalysis (blue dashed line). Observer positions are marked with *a*-*c* and correspond to $g_{\mathrm{eff}} = 2.061$, 2.071, and 2.315, respectively; Trace *a* corresponds to the $g_\perp$ region and trace *c* to $g_\parallel$. (**B**) Dipolar spectra detected at positions *a* (black solid line) and *c* (frequency axis scaled by $g_\perp^2/g_\parallel^2$, amplitude normalized to the Pake pattern intensity, dark red solid line) overlaid with a Pake pattern simulated by DeerAnalysis based on time trace *a* (blue dashed line); (**C**) Distance distributions obtained from experiment using PeldorFit (red solid line) and from MD simulations (grey solid line).

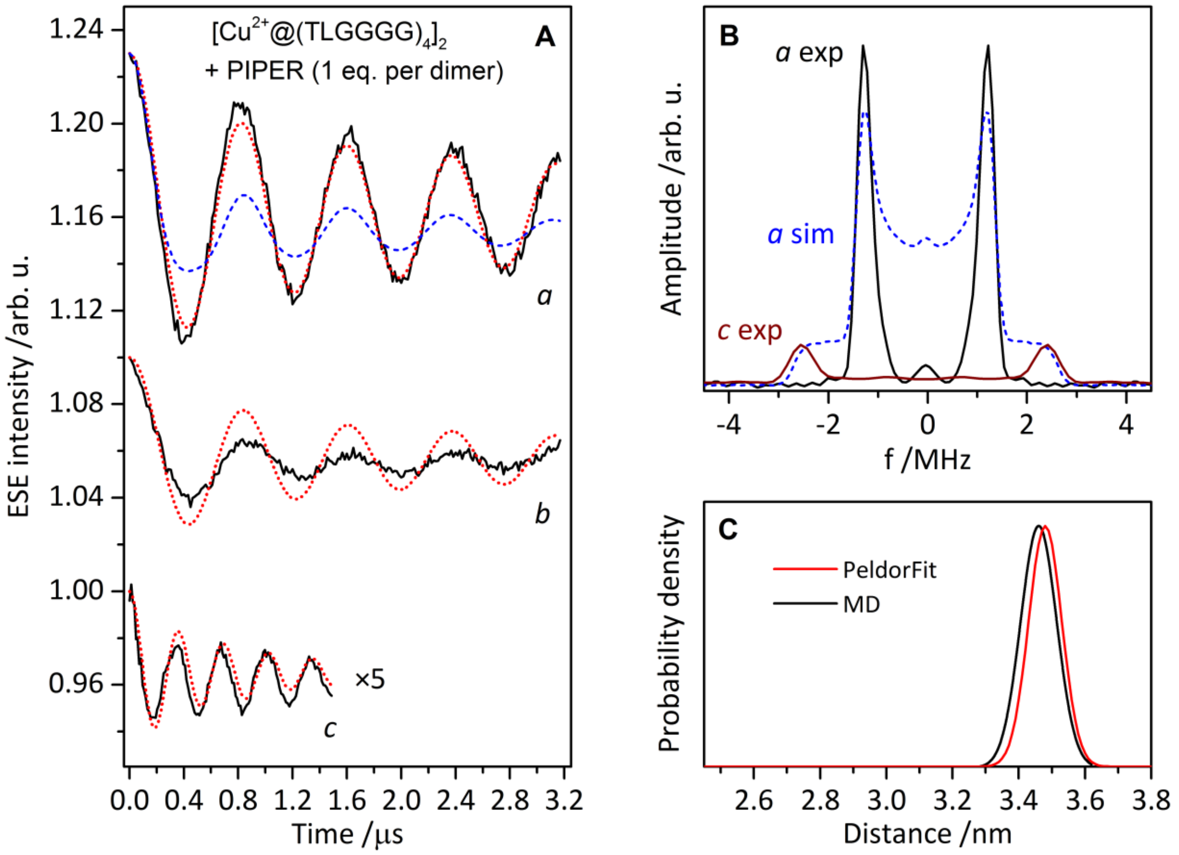

Fig. S39: (**A**) Background-corrected orientation-selective DEER time traces of $[Cu^{2+}@\mathbf{B}_4]_2$ with PIPER (1 equiv. per dimer) measured at three field positions (black solid lines) overlaid with the best fit results from PeldorFit (red dotted lines) and DeerAnalysis (blue dashed line). Observer positions are marked with *a*-*c* and correspond to $g_{eff} = 2.061$, 2.071, and 2.315, respectively; Trace *a* corresponds to the $g_\perp$ region and trace *c* to $g_\parallel$. (**B**) Dipolar spectra detected at positions *a* (black solid line) and *c* (frequency axis scaled by $g_\perp^2/g_\parallel^2$, amplitude normalized to the Pake pattern intensity, dark red solid line) overlaid with a Pake pattern simulated by DeerAnalysis based on time trace *a* (blue dashed line); (**C**) Distance distributions obtained from experiment using PeldorFit (red solid line) and from MD simulations (grey solid line).

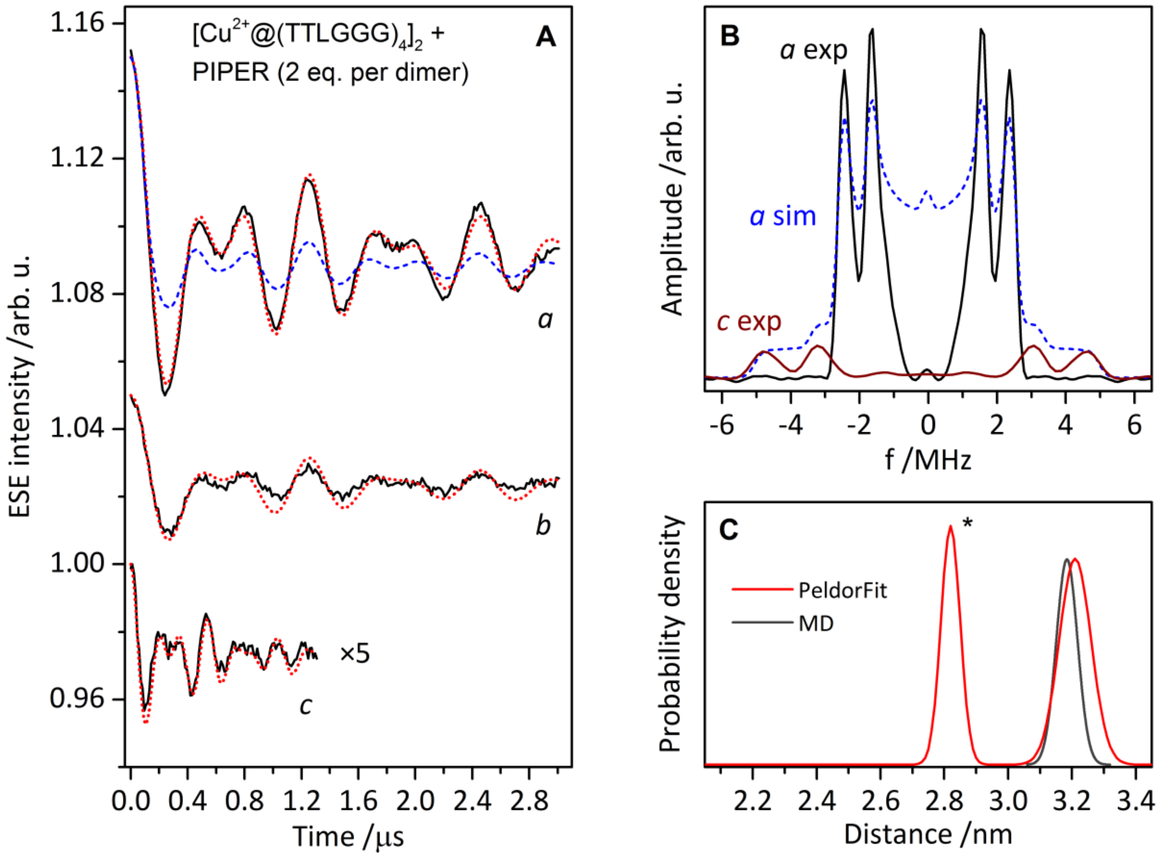

Fig. S40: (**A**) Background-corrected orientation-selective DEER time traces of $[Cu^{2+}@\mathbf{A}_4]_2$ with PIPER (2 equiv. per dimer) measured at three field positions (black solid lines) overlaid with the best fit results from PeldorFit (red dotted lines) and DeerAnalysis (blue dashed line). Observer positions are marked with *a*-*c* and correspond to $g_{\text{eff}} = 2.061$, 2.071, and 2.315, respectively; Trace *a* corresponds to the $g_{\perp}$ region and trace *c* to $g_{\parallel}$. (**B**) Dipolar spectra detected at positions *a* (black solid line) and *c* (frequency axis scaled by $g_{\perp}^2/g_{\parallel}^2$, amplitude normalized to the Pake pattern intensity, dark red solid line) overlaid with a Pake pattern simulated by DeerAnalysis based on time trace *a* (blue dashed line); (**C**) Distance distributions obtained from experiment using PeldorFit (red solid line) and from MD simulations (grey solid line). The asterisk marks the distance distribution originating from the PIPER@$[Cu^{2+}@\mathbf{A}_4]_2$ dimer subpopulation.

5.9 No Intercalation of PIPER into 5'-5' Stacked Dimers

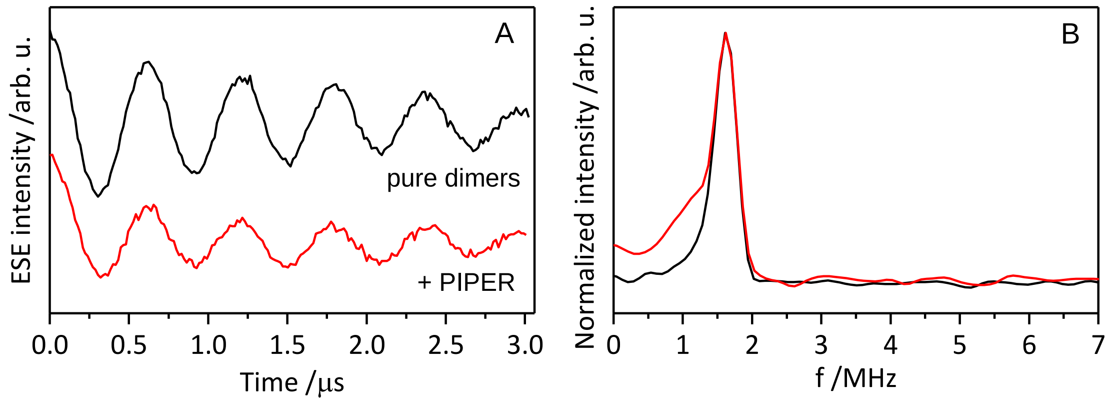

Fig. S41: (**A**) DEER time traces recorded at g_{eff} = 2.061 for [Cu^{2+}@$\mathbf{E}_4$] samples without PIPER (black trace) and with 1 equiv. of PIPER per dimer (red trace). (**B**) Corresponding dipolar spectra.

The lack of change in the dipolar frequency upon the addition of PIPER (1 equiv. per dimer) indicates that the dye does not intercalate into 5'-5' stacked dimers. However, DEER modulation depth somewhat decreased upon the PIPER addition (~8% and ~10% with and without PIPER, respectively), suggesting that the formation of monomeric adducts cannot be excluded.

5.10 Intercalation of Telomestatin into 3’-3’ Stacked Dimers

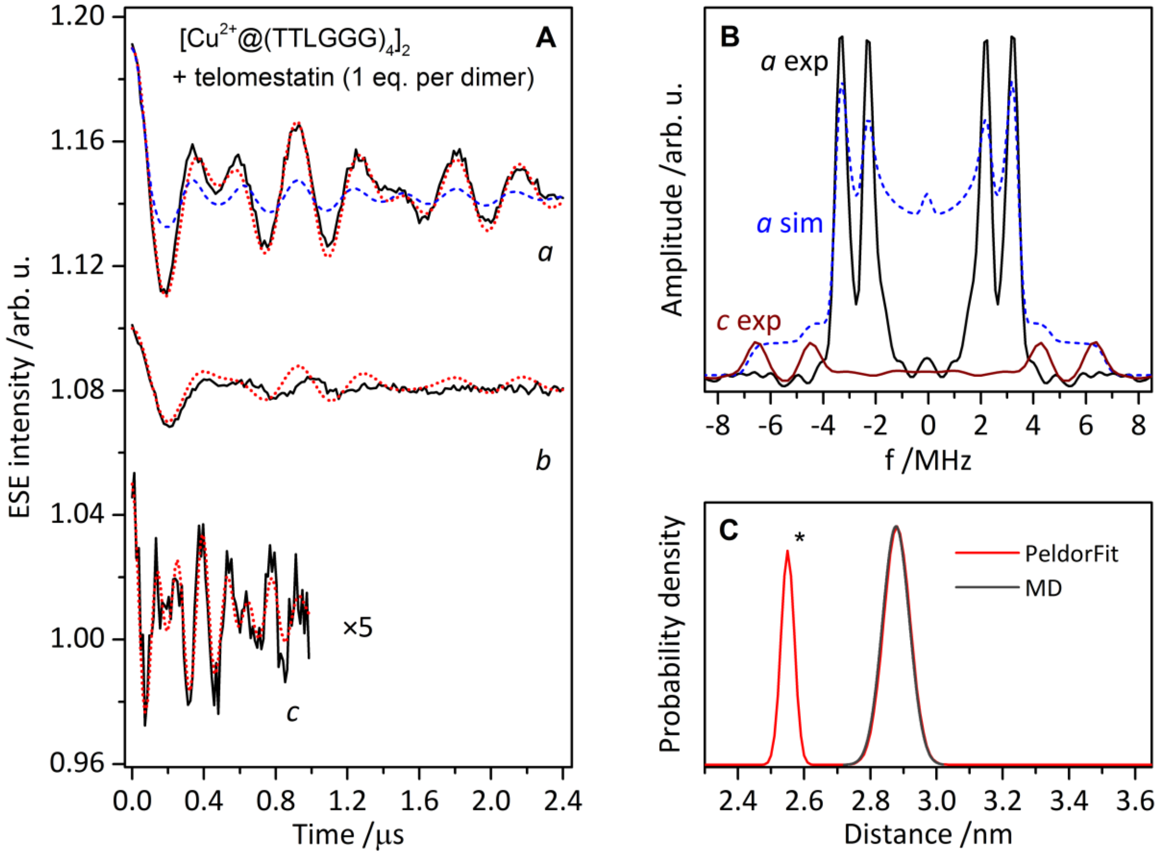

Fig. S42: (**A**) Background-corrected orientation-selective DEER time traces of [Cu^{2+}@**A**$_4$]$_2$ with telomestatin (1 equiv. per dimer) measured at three field positions (black solid lines) overlaid with the best fit results from PeldorFit (red dotted lines) and DeerAnalysis (blue dashed line). Observer positions are marked with *a*-*c* and correspond to g_{eff} = 2.061, 2.071, and 2.315, respectively; Trace *a* corresponds to the $g_\perp$ region and trace *c* to $g_\parallel$. (**B**) Dipolar spectra detected at positions *a* (black solid line) and *c* (frequency axis scaled by $g_\perp^2/g_\parallel^2$, amplitude normalized to the Pake pattern intensity, dark red solid line) overlaid with a Pake pattern simulated by DeerAnalysis based on time trace *a* (blue dashed line); (**C**) Distance distributions obtained from experiment using PeldorFit (red solid line) and from MD simulations (grey solid line). The asterisk marks the distance distribution originating from the pure [Cu^{2+}@**A**$_4$]$_2$ dimer subpopulation.

5.11 Intercalation of Free G-Tetrads into 3'-3' Stacked Dimers

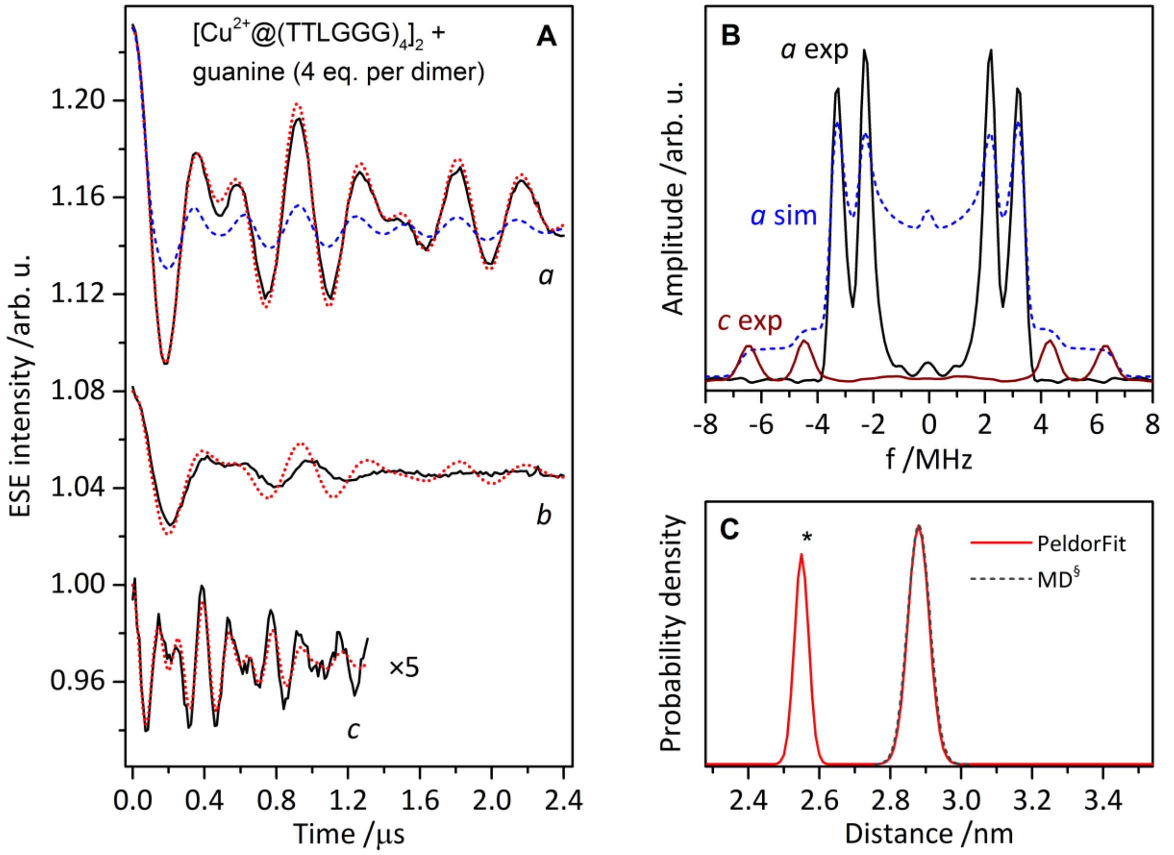

Fig. S43: (A) Background-corrected orientation-selective DEER time traces of [Cu^{2+}@**A**$_4$]$_2$ with guanine (4 equiv. per dimer) measured at three field positions (black solid lines) overlaid with the best fit results from PeldorFit (red dotted lines) and DeerAnalysis (blue dashed line). Observer positions are marked with *a*-*c* and correspond to g_{eff} = 2.061, 2.071, and 2.315, respectively; Trace *a* corresponds to the $g_{\perp}$ region and trace *c* to $g_{\parallel}$. (**B**) Dipolar spectra detected at positions *a* (black solid line) and *c* (frequency axis scaled by $g_{\perp}^2/g_{\parallel}^2$, amplitude normalized to the Pake pattern intensity, dark red solid line) overlaid with a Pake pattern simulated by DeerAnalysis based on time trace *a* (blue dashed line); (**C**) Distance distributions obtained from experiment using PeldorFit (red solid line) and from MD simulations (grey dashed line). §The MD results are shown for guanosine G-tetrads. The asterisk marks the distance distribution originating from the pure [Cu^{2+}@**A**$_4$]$_2$ dimer subpopulation.

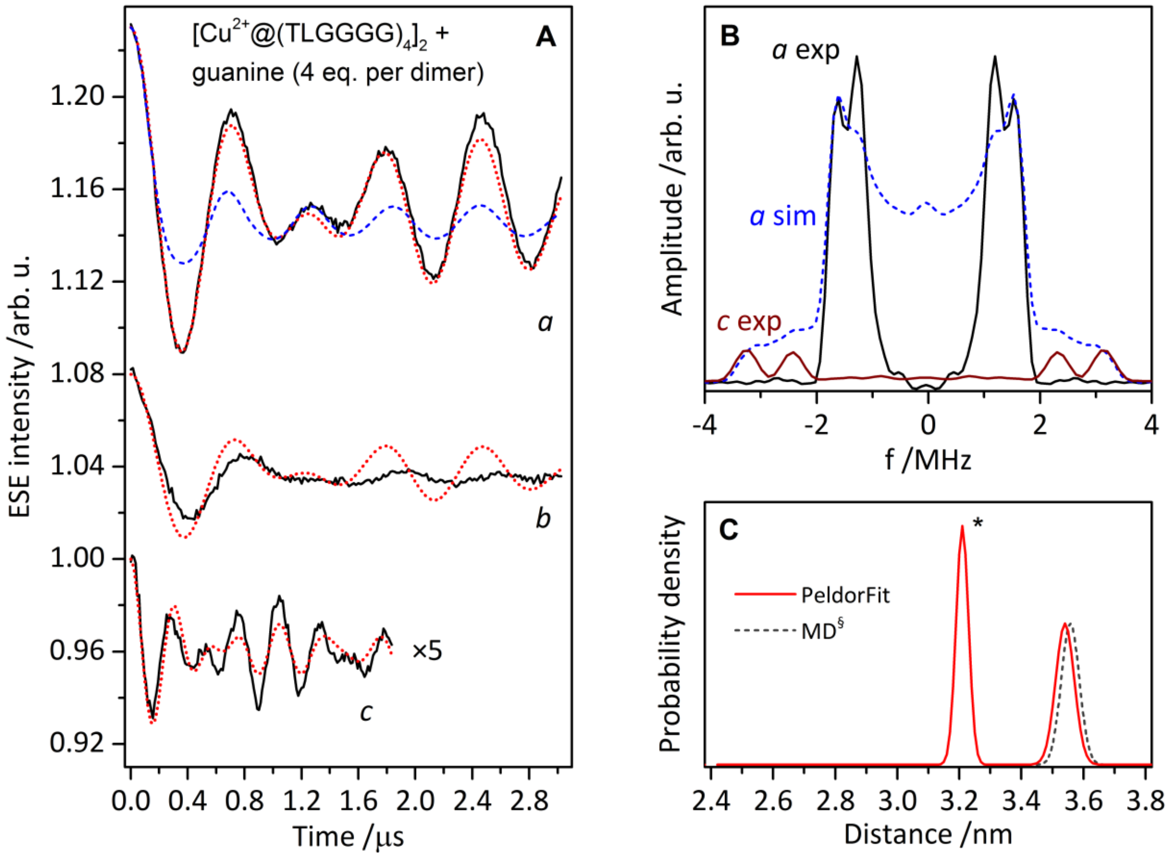

Fig. S44: (A) Background-corrected orientation-selective DEER time traces of $[Cu^{2+}@\mathbf{B}_4]_2$ with guanine (4 equiv. per dimer) measured at three field positions (black solid lines) overlaid with the best fit results from PeldorFit (red dotted lines) and DeerAnalysis (blue dashed line). Observer positions are marked with *a*-*c* and correspond to g_{eff} = 2.061, 2.071, and 2.315, respectively; Trace *a* corresponds to the $g_{\perp}$ region and trace *c* to $g_{\parallel}$. (**B**) Dipolar spectra detected at positions *a* (black solid line) and *c* (frequency axis scaled by $g_{\perp}^2/g_{\parallel}^2$, amplitude normalized to the Pake pattern intensity, dark red solid line) overlaid with a Pake pattern simulated by DeerAnalysis based on time trace *a* (blue dashed line); (**C**) Distance distributions obtained from experiment using PeldorFit (red solid line) and from MD simulations (grey dashed line). §The MD results are shown for guanosine G-tetrads. The asterisk marks the distance distribution originating from the pure $[Cu^{2+}@\mathbf{B}_4]_2$ dimer subpopulation.

Upon the intercalation of guanine tetrads into $[Cu^{2+}@\mathbf{A}_4]_2$ and $[Cu^{2+}@\mathbf{B}_4]_2$, the Cu-Cu distance increased by Δd = 0.33 nm for both types of dimers. However, due to the $1/d^3$ dependence of dipolar frequency on the interspin distance, the same Δd resulted in a much smaller dipolar frequency separation between $guanine_4@[Cu^{2+}@\mathbf{B}_4]_2$ and $[Cu^{2+}@\mathbf{B}_4]_2$ ($\Delta\nu_B \approx$ 0.4 MHz) than between $guanine_4@[Cu^{2+}@\mathbf{A}_4]_2$ and $[Cu^{2+}@\mathbf{A}_4]_2$ ($\Delta\nu_A \approx 1.0$ MHz).

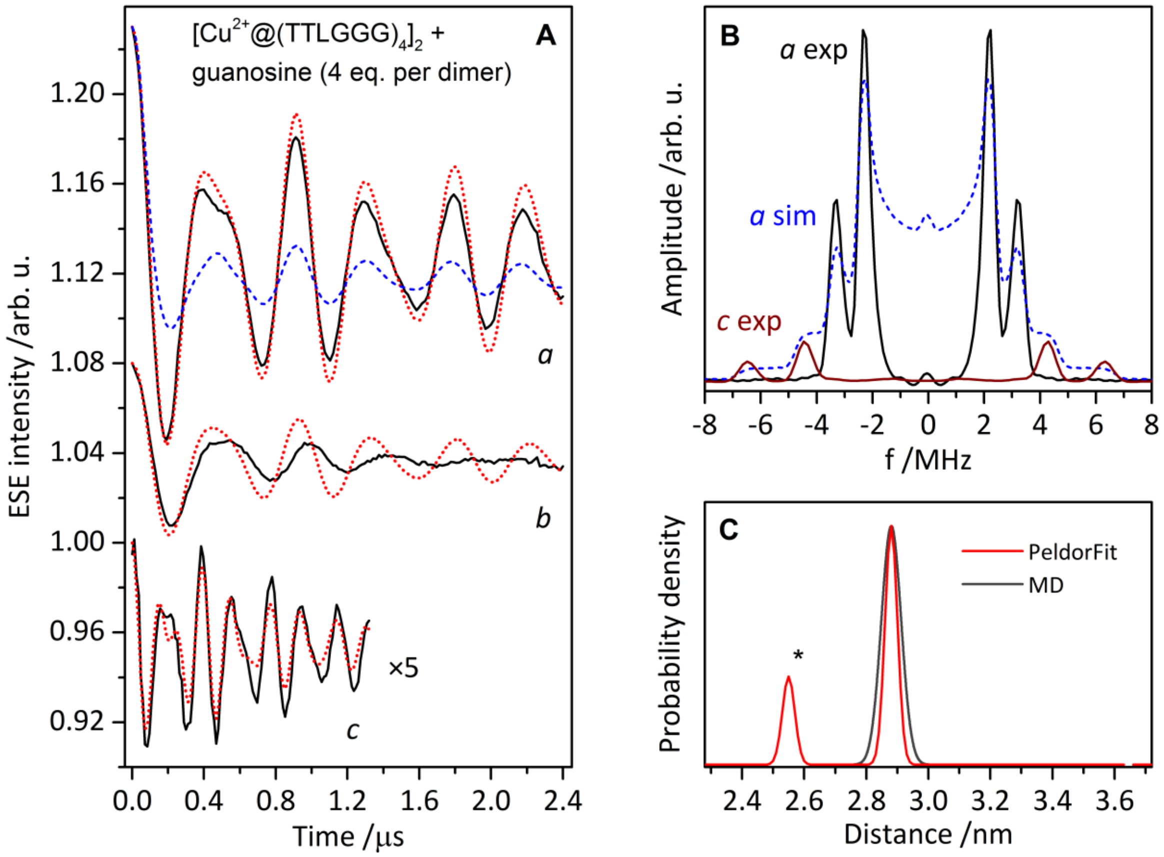

Fig. S45: (A) Background-corrected orientation-selective DEER time traces of $[Cu^{2+}@\mathbf{A}_4]_2$ with guanosine (4 equiv. per dimer) measured at three field positions (black solid lines) overlaid with the best fit results from PeldorFit (red dotted lines) and DeerAnalysis (blue dashed line). Observer positions are marked with *a*-*c* and correspond to g_{eff} = 2.061, 2.071, and 2.315, respectively; Trace *a* corresponds to the $g_{\perp}$ region and trace *c* to $g_{\parallel}$. (**B**) Dipolar spectra detected at positions *a* (black solid line) and *c* (frequency axis scaled by $g_{\perp}^2/g_{\parallel}^2$, amplitude normalized to the Pake pattern intensity, dark red solid line) overlaid with a Pake pattern simulated by DeerAnalysis based on time trace *a* (blue dashed line); (**C**) Distance distributions obtained from experiment using PeldorFit (red solid line) and from MD simulations (grey solid line). The asterisk marks the distance distribution originating from the pure $[Cu^{2+}@\mathbf{A}_4]_2$ dimer subpopulation.

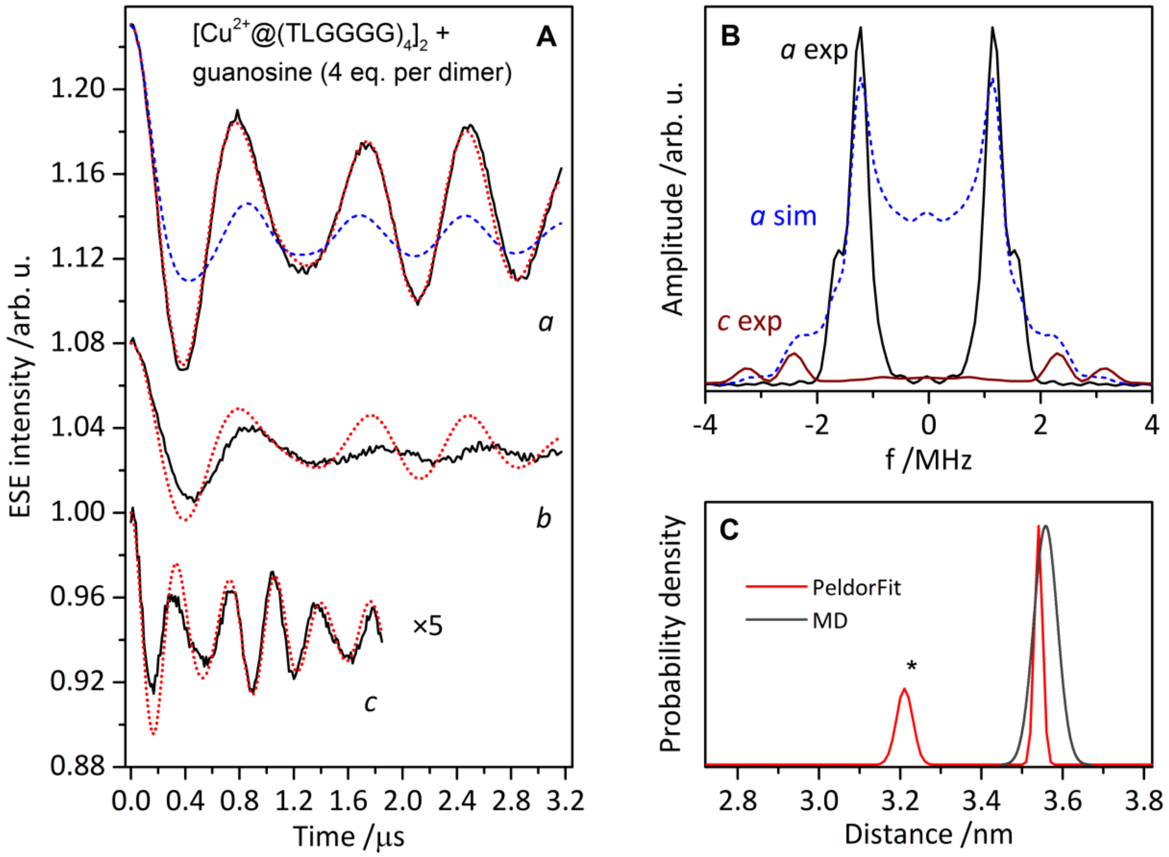

Fig. S46: (A) Background-corrected orientation-selective DEER time traces of $[Cu^{2+}@\mathbf{B}_4]_2$ with guanosine (4 equiv. per dimer) measured at three field positions (black solid lines) overlaid with the best fit results from PeldorFit (red dotted lines) and DeerAnalysis (blue dashed line). Observer positions are marked with *a*-*c* and correspond to g_{eff} = 2.061, 2.071, and 2.315, respectively; Trace *a* corresponds to the $g_{\perp}$ region and trace *c* to $g_{\parallel}$. (**B**) Dipolar spectra detected at positions *a* (black solid line) and *c* (frequency axis scaled by $g_{\perp}^2/g_{\parallel}^2$, amplitude approximately normalized to the Pake pattern intensity, dark red solid line) overlaid with a Pake pattern simulated by DeerAnalysis based on time trace *a* (blue dashed line); (**C**) Distance distributions obtained from experiment using PeldorFit (red solid line) and from MD simulations (grey solid line). The asterisk marks the distance distribution originating from the pure $[Cu^{2+}@\mathbf{B}_4]_2$ dimer subpopulation.

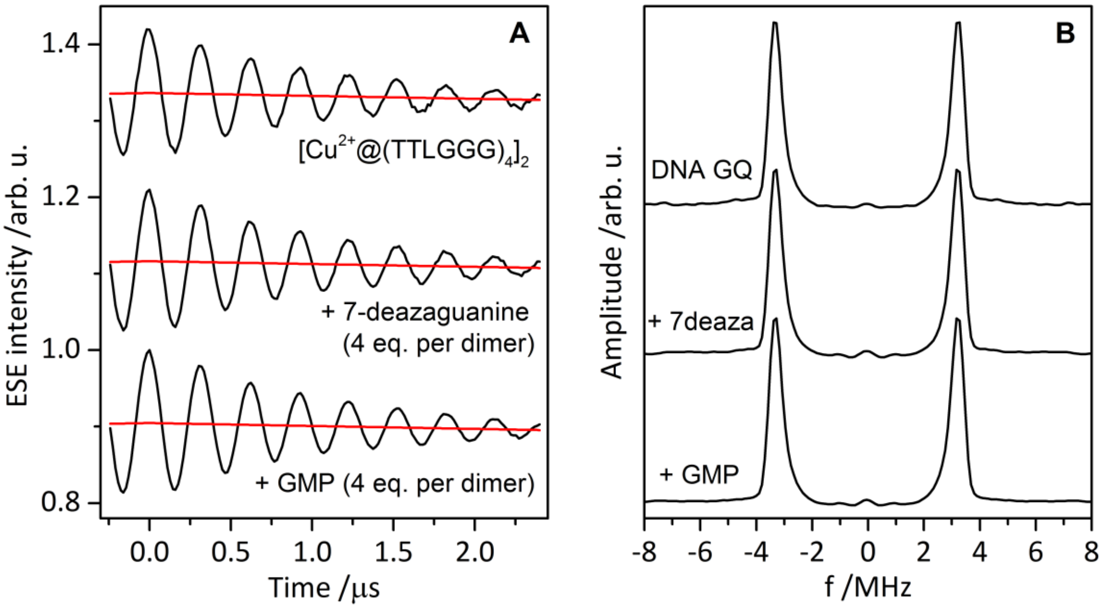

Fig. S47: (A) Primary DEER traces (black) overlaid with the background fits (red) at the observer position g_{eff}= 2.061 for the pure $[Cu^{2+}@\mathbf{A}_4]_2$ dimers (top trace) and with the addition of 7-deazaguanine (middle trace) and guanosine monophosphate (GMP, bottom trace). **(B)** Comparison of the corresponding dipolar spectra demonstrates that 7-deazaguanine and GMP did not intercalate into the 3'-3'stacked dimers.

6 Molecular Dynamics (MD) Simulations

MD simulations were carried out as previously described[4,5] using the Gromacs 2019.2 program,[17–19] the AMBER force field ff99bsc1[20,21] for nucleic acid parts and the General Amber Force Field (GAFF)[22] for the PIPER cation and the telomestatin molecule.

6.1 Generation of Missing Parameters

Force field parameters and RESP charges for the artificial nucleotide **L** and its Cu^{2+} complex were generated in earlier studies[4] and used without changes.
To obtain RESP charges[23] for the PIPER cation and telomestatin, the ESP were calculated at Hartree-Fock level with a 6-31G* basis set and RESP charges were obtained by a two-stages fitting procedure using Antechamber in the AmberTools19 package.[24] Also GAFF atom types were determined using Antechamber. Topology files for Gromacs were obtained using the LEaP program in the AmberTools19 package and the ACPYPE program.[25]

6.2 Generation of Initial Structures

First, initial structures for the G-quadruplex monomers ([Cu^{2+}@**A**$_4$], [Cu^{2+}@**B**$_4$], [Cu^{2+}@**D**$_4$] and [Cu^{2+}@**E**$_4$]) were constructed by using the solid-state structure of the G-quadruplex dimer [$(TG_4T)_4$]$_2$ (PDB entry 2O4F).[26] All manipulations were carried out in UCSF Chimera.[27] Na^+ ions were replaced by K^+ ions. The second monomer, water molecules, redundant ions and redundant nucleotides were deleted, the geometry-optimized Cu^{2+}-complex was inserted manually and missing nucleotides were duplicated and also inserted manually.

In a next step, initial structures for G-quadruplex dimers were generated. Therefore, the monomer structures were duplicated and arranged to tail-to-tail ([Cu^{2+}@**A**$_4$]$_2$, [Cu^{2+}@**B**$_4$]$_2$) or head-to-head dimers ([Cu^{2+}@**D**$_4$]$_2$ and [Cu^{2+}@**E**$_4$]$_2$), respectively. G-tetrad stacking distances at the interface of the stacked G-quadruplexes were set in the same range as G-tetrad stacking distances within the G-quartet core. The relative rotation angles at the interfaces were set to a '6-ring' stacking mode for tail-to-tail dimers and to a '5/6-ring' stacking mode for head-to-head dimers, respectively.[28] A K^+ ion was placed between the stacking G-tetrads at the interface, which was found in a dimeric solid-state structure of an unmodified tetramolecular G-quadruplex.[26]

To generate initial structures with a free guanosine quartet intercalating between the two monomers of a dimer, one deoxyguanosine tetrad was cut out from the above-mentioned solid-state structure of a G-quadruplex[26] and the 2'-OH groups were added manually. The free guanosine tetrad was then inserted manually between the two monomers of a dimer. The π-stacking distances were set in the same range as the ones within the G-tetrad cores. Relative rotation angles at the interfaces between G-quadruplexes and free guanosine tetrads were set according to a '6-ring' stacking mode for opposite-polarity stacking and to a 'partial 5/6-ring' stacking mode for same-polarity stacking, respectively.[28] One K^+ ion was placed into every stacking interspace.

To generate initial structures with PIPER or telomestatin, respectively, intercalating between the two monomers of a dimer, the geometry optimized intercalators were inserted manually between the two monomers. The π-stacking distances were set in the same range as the ones within the G-tetrad cores. For telomestatin as the intercalator, one K^+ ion was placed into every stacking interspace between telomestatin and the quadruplex monomers.[29] No K^+ ions were placed into the stacking interspaces between PIPER and the quadruplex monomers.[30] For PIPER as the intercalator, the piperidine side chains were pointing into the grooves of the G-quadruplexes. To create a starting structure for complex 2PIPER@[Cu^{2+}@**A**$_4$]$_2$, the two PIPER molecules were aligned in an orthogonal way to each other.[30]

6.3 MD Simulation Procedure

The respective models obtained above were put in a periodic rhombic dodecahedron box (cutoff 1.5 nm) and energy minimized 2000 steps of steepest descent (600 kJ/mol nm tolerance) in vacuum. PME and van-der-Waals cutoff of 1.3 nm were used. The system was solvated with TIP3P water molecules and the negative charge of the system was neutralized with the corresponding amount of randomly positioned K^+ ions. An additional 100 mmol/L KCl was added to simulate the ionic strength of the experiments.

The system was then energy minimized in two steps, first 500 steps of steepest descent (500 kJ/mol nm tolerance) and then 3000 steps of conjugate gradient minimization (300 kJ/mol nm tolerance). The non-bonded Lennard-Jones cutoff was set to 1.3 nm, the non-bonded pair list updated every 50 steps. For the coulombic interactions, Particle-mesh Ewald summation (PME) was used.[31] Next, the system was equilibrated with positional constraints on the model's heavy-atoms 100 ps in a first round (NVT ensemble, constraints

1000 kJ/mol Å^2, time step 2 fs; Temperature coupling modified Berendsen, 298 K); second round 100 ps with additional pressure coupling (isotropic, Berendsen, 1 bar, time constant for coupling 0.1 ps, compressibility 4.5 10^{-5}) and a third round 100 ps but with lower constraints (100 kJ/mol Å^2, Nose-Hoover temperature coupling, 2 ps coupling, Parinello-Rahman isotroic pressure coupling, 2 ps coupling time). The equilibration phase was finished with 200 ps of an unconstraint DNA MD run (coupling times increased to 4 ps). A 50 ns MD production run was then performed. Coordinates were written every 10 ps, resulting in 5001 frames per trajectory. Trajectories were centered, aligned and fitted to the first frame using the built-in Gromacs tools and then analyzed and visualized with UCSF Chimera.[27] For comparison of the simulated Cu^{2+}-Cu^{2+} distance distributions with the experimentally derived ones (see Fig. S30–46), the Cu^{2+}-Cu^{2+} distances were extracted from each frame and the 5001 distance values were used to plot a distribution curve with the Origin software (type of distribution curve: normal).

6.4 MD-derived Structures, Cu^{2+}–Cu^{2+} Distances and RMSD Trajectories

The average Cu^{2+}-Cu^{2+} distances and standard deviations were calculated based on the distances of the complete trajectories extracted with Chimera. The depicted structures are representatives of the MD trajectories. For the RMSD plots, the first frame of the trajectory was used as the reference.

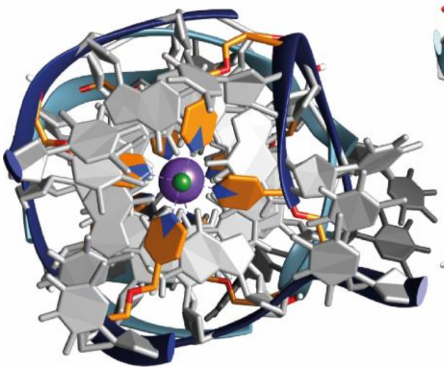

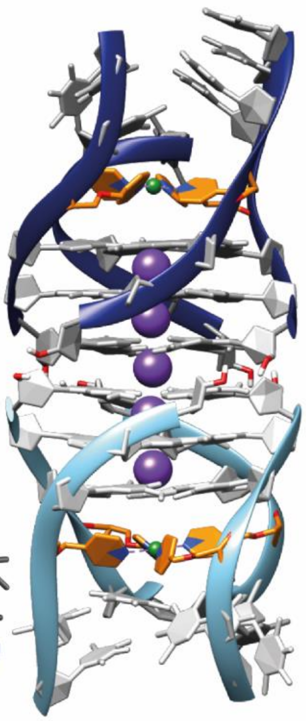

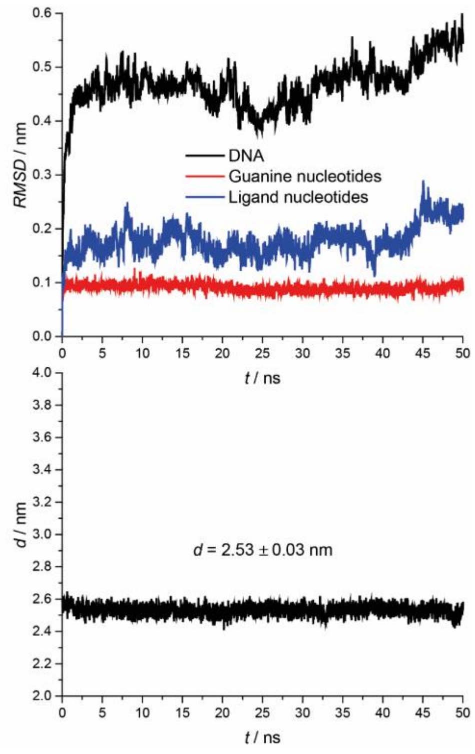

Fig. S48:
MD-derived structural model of the G-quadruplex dimer $[Cu^{2+}@\mathbf{A}_4]_2$ (top view and side view), the Cu^{2+}-Cu^{2+} distance throughout the full MD simulation and the corresponding RMSD plots.

Fig. S49:
MD-derived structural model of the G-quadruplex dimer $[Cu^{2+}@\mathbf{B}_4]_2$ (top view and side view), the Cu^{2+}-Cu^{2+} distance throughout the full MD simulation and the corresponding RMSD plots.

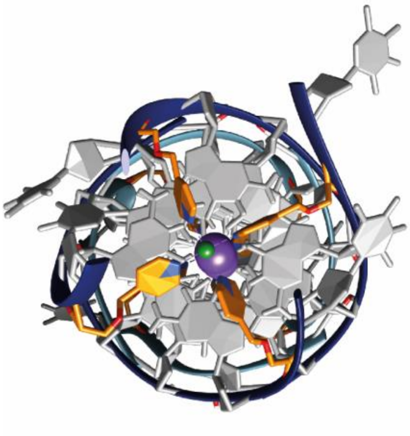

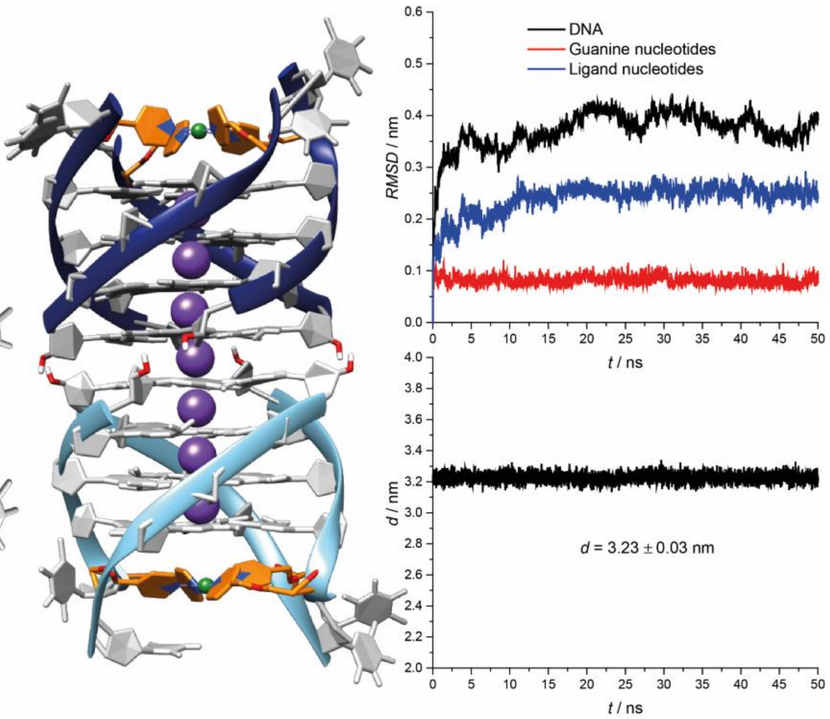

Fig. S50:
MD-derived structural model of the G-quadruplex dimer $[Cu^{2+}@\mathbf{D}_4]_2$ (top view and side view), the Cu^{2+}-Cu^{2+} distance throughout the full MD simulation and the corresponding RMSD plots.

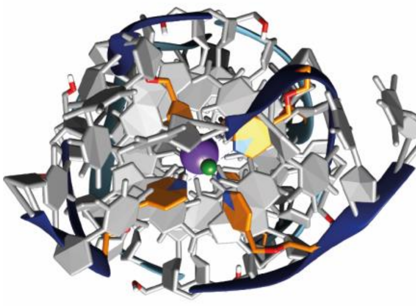

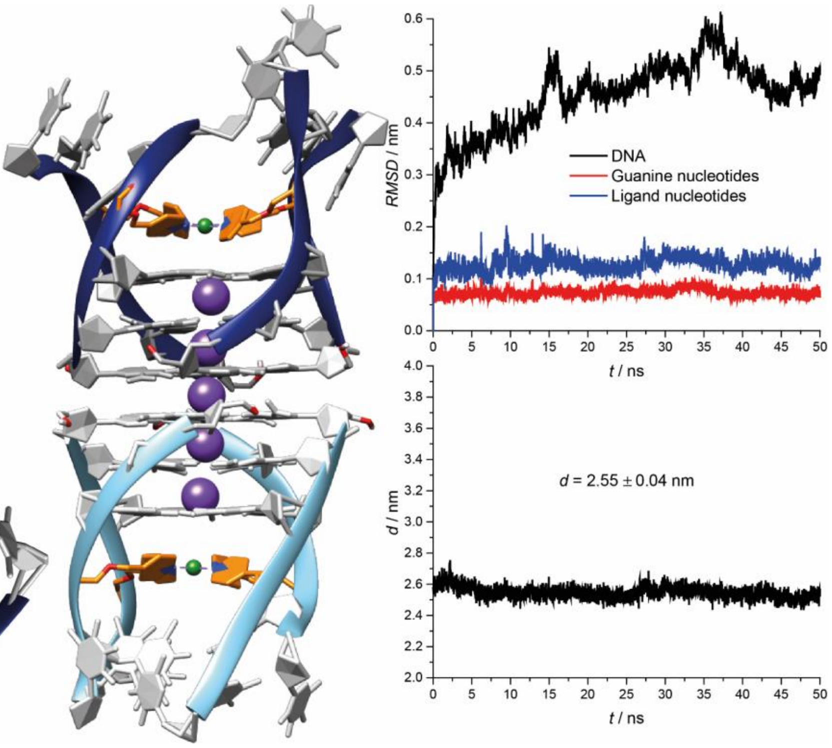

Fig. S51:
MD-derived structural model of the G-quadruplex dimer [Cu^{2+}@**E**$_4$]$_2$ (top view and side view), the Cu^{2+}-Cu^{2+} distance throughout the full MD simulation and the corresponding RMSD plots.

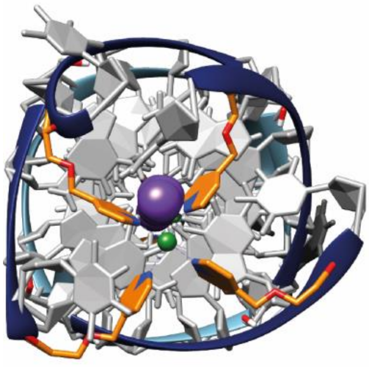

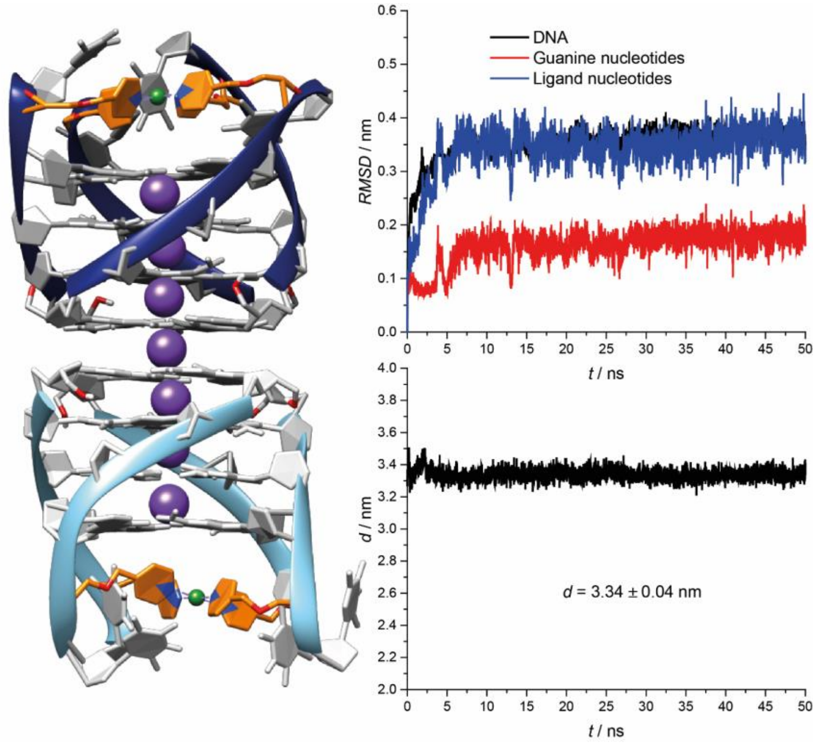

Fig. S52:
MD-derived structural model of the sandwich-type complex guoanosine$_4$@[Cu^{2+}@**A**$_4$]$_2$ (top view and side view, the free guanosine tetrad is shown in green), the Cu^{2+}-Cu^{2+} distance throughout the full MD simulation and the corresponding RMSD plots.

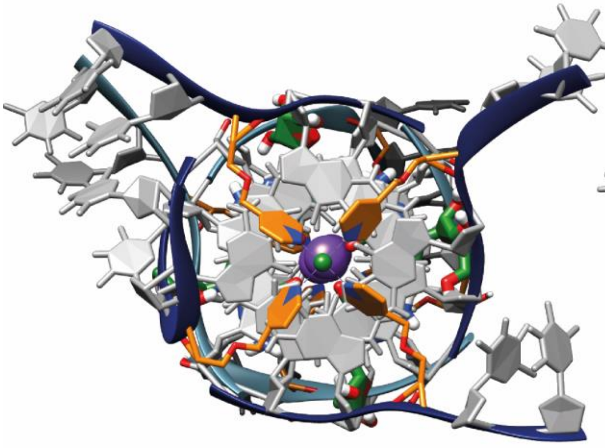

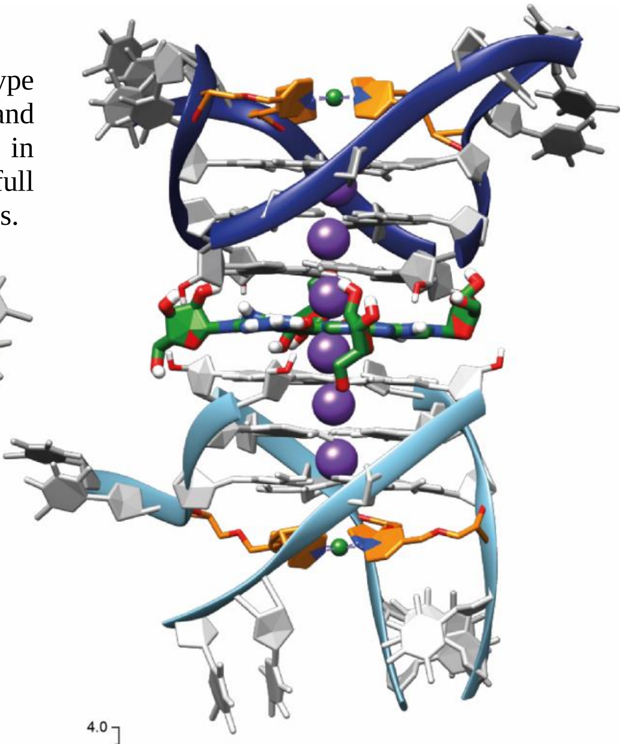

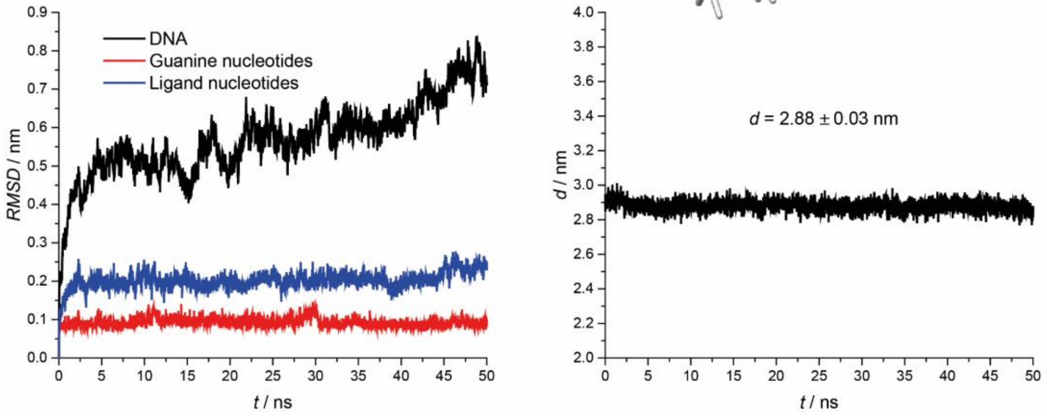

Fig. S53:
MD-derived structural model of the sandwich-type complex guoanosine$_4$@[Cu^{2+}@**B**$_4$]$_2$ (top view and side view, the free guanosine tetrad is shown in green), the Cu^{2+}-Cu^{2+} distance throughout the full MD simulation and the corresponding RMSD plots.

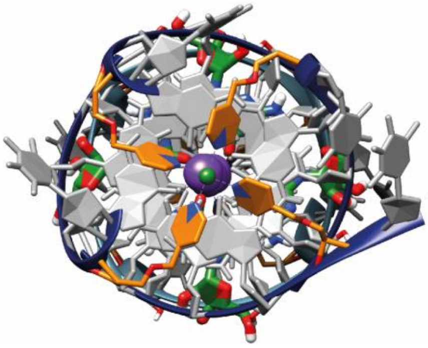

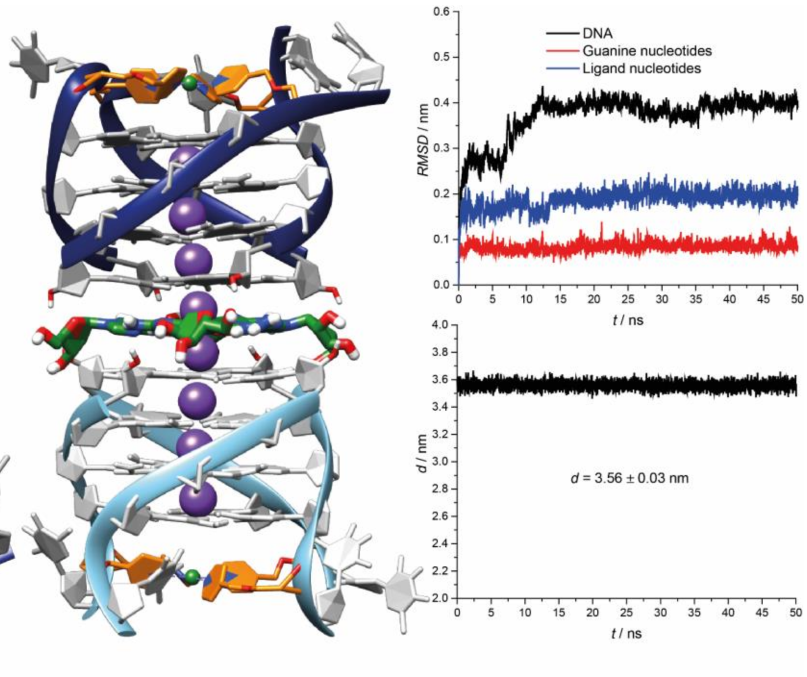

Fig. S54:
MD-derived structural model of the sandwich-type complex PIPER@[Cu^{2+}@**A**$_4$]$_2$ (top and side view, PIPER is shown in red), the Cu^{2+}-Cu^{2+} distance throughout the full MD simulation and the respective RMSD plots.

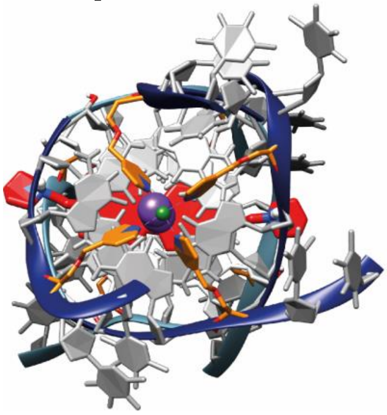

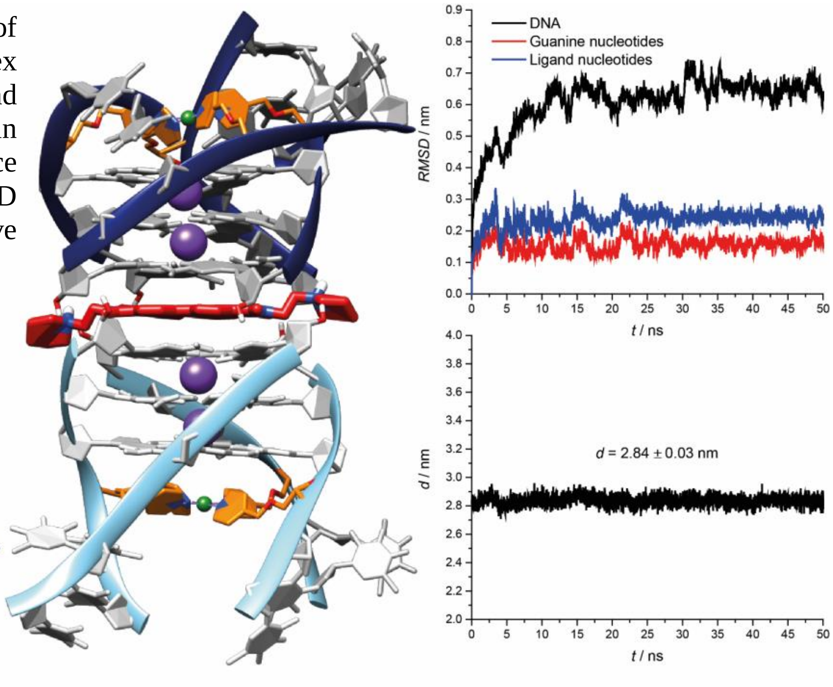

Fig. S55:
MD-derived structural model of the sandwich-type complex PIPER@[Cu^{2+}@**B**$_4$]$_2$ (top and side view, PIPER is shown in red), the Cu^{2+}-Cu^{2+} distance throughout the full MD simulation and the respective RMSD plots.

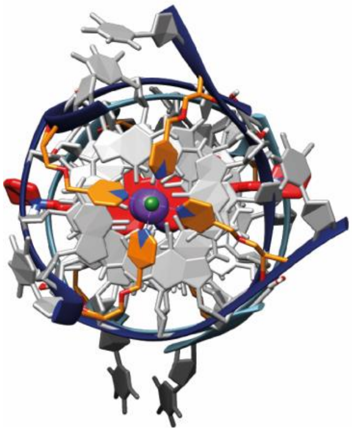

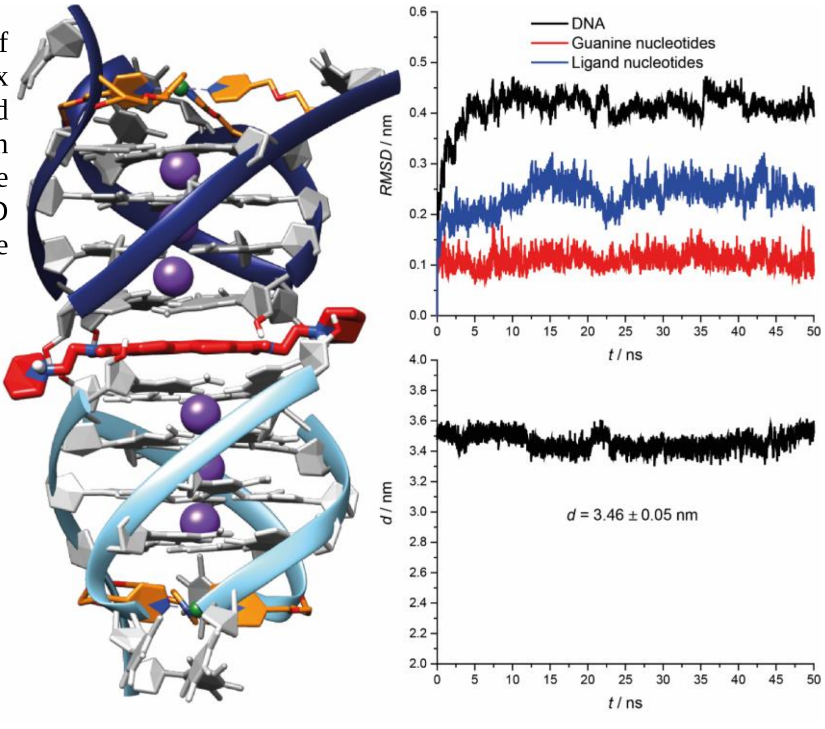

Fig. S56:
MD-derived structural model of the sandwich-type complex telomestatin@[Cu^{2+}@**A**$_4$]$_2$ (top and side view, telomestatin is shown in black), the Cu^{2+}-Cu^{2+} distance throughout the full MD simulation and the respective RMSD plots.

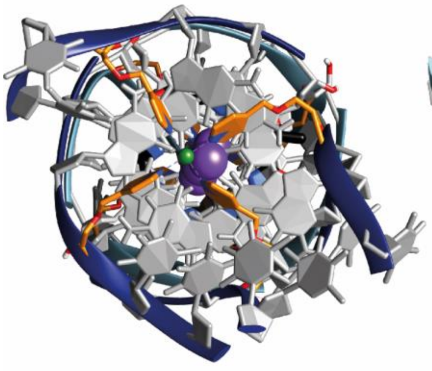

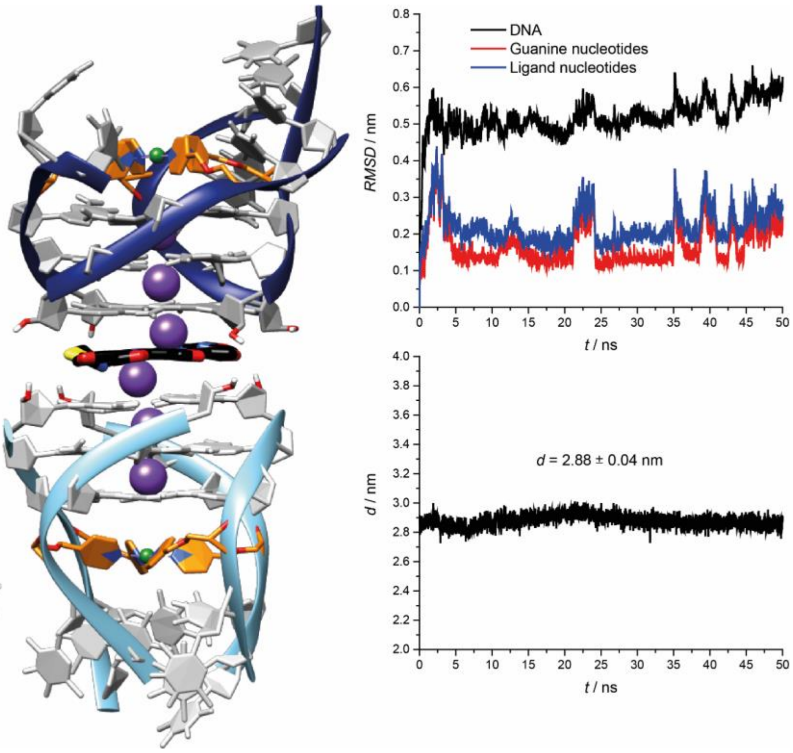

The slightly different effects on the extended Cu^{2+}-Cu^{2+} distances in the sandwich complexes caused by the macrocyclic intercalators (telomestatin and free G-quartets composed of either guanines or guanosines, Δd = 0.31 – 0.34 nm, Table 2) on the one hand, and PIPER (Δd = 0.27 – 0.28 nm) on the other hand, that were observed in the DEER experiments, were reproduced in the MD simulations. These differences might be caused by additional electrostatic interactions between the DNA and positively charged PIPER (while the other

intercalators are neutral). Another possible explanation deals with the presence of additional K^+ ions in the interspaces between the quadruplex monomers and the macrocyclic intercalators, where the K^+ ions interact with the inwards pointing donor groups of the macrocycles (we inserted the additional K^+ ions in the starting structures). On the other hand, additional K^+ ions in the interspaces between quadruplex monomers and PIPER seemed unreasonable and we did not insert them in the starting structures.

Fig. S57:
MD-derived structural model of the sandwich-type complex 2PIPER@[Cu^{2+}@**A**$_4$]$_2$ (top and side view, PIPER is shown in red), the Cu^{2+}-Cu^{2+} distance throughout the full MD simulation and the respective RMSD plots. Also, the two intercalating PIPER molecules are shown from different perspectives to highlight their relative orientation with respect to each other (relative rotation angle of about 40 – 60° throughout the MD run).

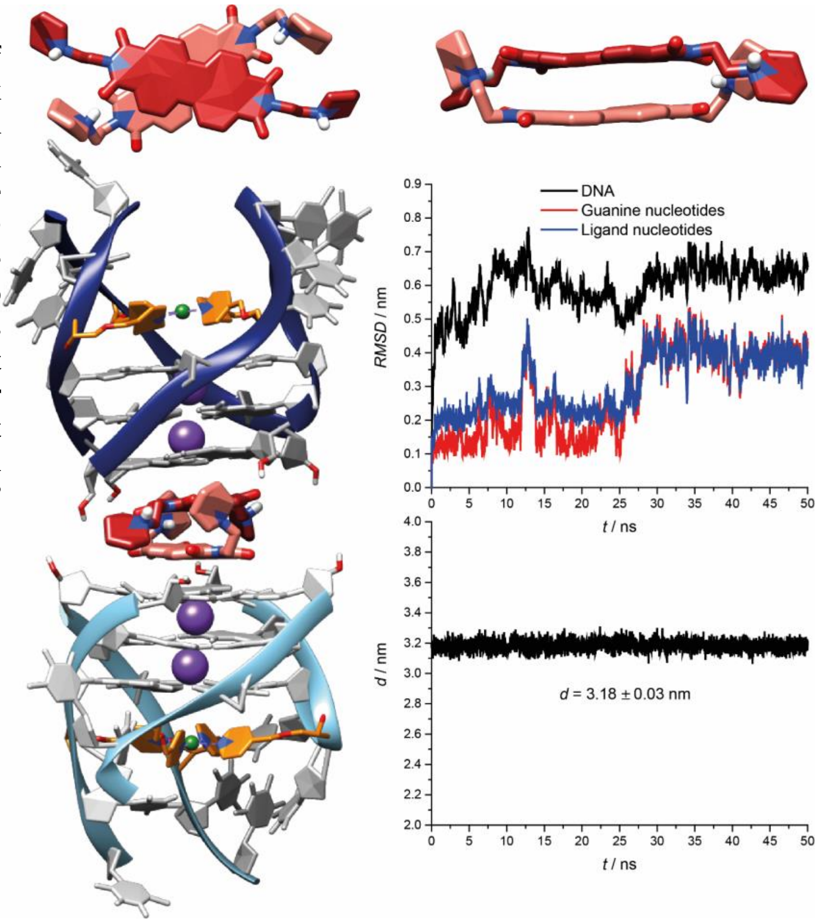

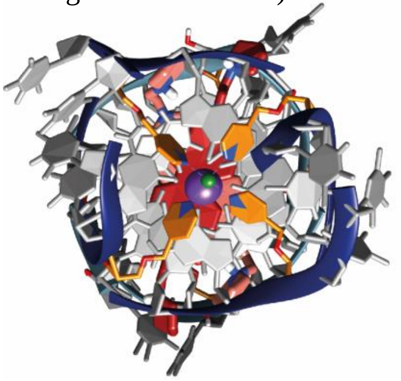

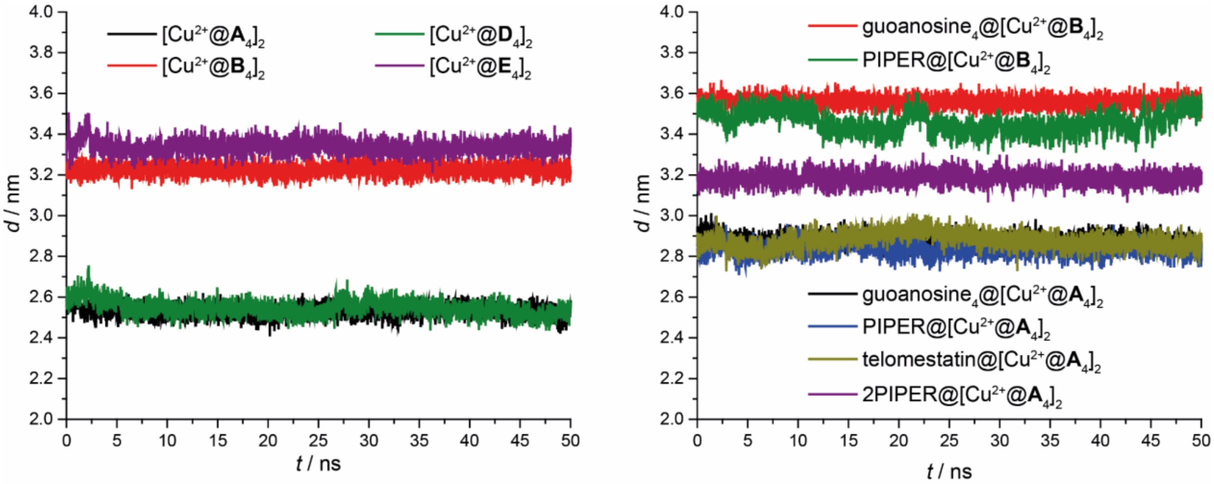

Fig. S58: Comparison of the Cu^{2+}-Cu^{2+} distances of different G-quadruplex dimers and related sandwich complexes derived from MD simulations.

7 References

[1] O. Y. Fedoroff, M. Salazar, H. Han, V. V. Chemeris, S. M. Kerwin, L. H. Hurley, *Biochemistry* **1998**, *37*, 12367–12374.

[2] C. Sissi, L. Lucatello, A. P. Krapcho, D. J. Maloney, M. B. Boxer, M. V. Camarasa, G. Pezzoni, E. Menta, M. Palumbo, *Bioorg. Med. Chem.* **2007**, *15*, 555–562.

[3] W. Tuntiwechapikul, T. Taka, M. Béthencourt, L. Makonkawkeyoon, T. R. Lee, *Bioorg. Med. Chem. Lett.* **2006**, *16*, 4120–4126.

[4] D. M. Engelhard, J. Nowack, G. H. Clever, *Angew. Chem. Int. Ed.* **2017**, *56*, 11640–11644.

[5] D. M. Engelhard, L. M. Stratmann, G. H. Clever, *Chem. Eur. J.* **2018**, *24*, 2117–2125.

[6] M. J. Cavaluzzi, P. N. Borer, *Nucleic Acids Res.* **2004**, *32*, e13.

[7] Q. Zhai, M. Deng, L. Xu, X. Zhang, X. Zhou, *Bioorg. Med. Chem. Lett.* **2012**, *22*, 1142–1145.

[8] J.-L. Mergny, J. Li, L. Lacroix, S. Amrane, J. B. Chaires, *Nucleic Acids Res.* **2005**, *33*, e138–e138.

[9] J.-L. Mergny, L. Lacroix, *Curr. Protoc. Nucleic Acid Chem.* **2009**, *Chapter 17*, 17.1.1-17.1.15.

[10] S. Allenmark, *Chirality* **2003**, *15*, 409–422.

[11] S. Stoll, A. Schweiger, *J. Magn. Reson.* **2006**, *178*, 42–55.

[12] M. Teucher, E. Bordignon, *J. Magn. Reson.* **2018**, *296*, 103–111.

[13] G. Jeschke, V. Chechik, P. Ionita, A. Godt, H. Zimmermann, J. Banham, C. R. Timmel, D. Hilger, H. Jung, *Appl. Magn. Reson.* **2006**, *30*, 473–498.

[14] D. Abdullin, G. Hagelueken, R. I. Hunter, G. M. Smith, O. Schiemann, *Mol. Phys.* **2015**, *113*, 544–560.

[15] D. M. Engelhard, A. Meyer, A. Berndhäuser, O. Schiemann, G. H. Clever, *Chem. Commun.* **2018**, *54*, 7455–7458.

[16] Y. Kato, T. Ohyama, H. Mita, Y. Yamamoto, *J. Am. Chem. Soc.* **2005**, *127*, 9980–9981.

[17] S. Pronk, S. Páll, R. Schulz, P. Larsson, P. Bjelkmar, R. Apostolov, M. R. Shirts, J. C. Smith, P. M. Kasson, D. van der Spoel, et al., *Bioinformatics* **2013**, *29*, 845–54.

[18] B. Hess, H. Bekker, H. J. C. Berendsen, J. G. E. M. Fraaije, *J. Comput. Chem.* **1997**, *18*, 1463–1472.

[19] D. V. D. Spoel, E. Lindahl, B. Hess, G. Groenhof, A. E. Mark, H. J. C. Berendsen, *J. Comput. Chem.* **2005**, *26*, 1701–1718.

[20] A. Pérez, I. Marchán, D. Svozil, J. Sponer, T. E. Cheatham, C. A. Laughton, M. Orozco, *Biophys. J.* **2007**, *92*, 3817–3829.

[21] I. Ivani, P. D. Dans, A. Noy, A. Pérez, I. Faustino, A. Hospital, J. Walther, P. Andrio, R. Goñi, A. Balaceanu, et al., *Nat. Methods* **2016**, *13*, 55–58.

[22] J. Wang, R. M. Wolf, J. W. Caldwell, P. A. Kollman, D. A. Case, *J. Comput. Chem.* **2004**, *25*, 1157–1174.

[23] C. I. Bayly, P. Cieplak, W. Cornell, P. A. Kollman, *J. Phys. Chem.* **1993**, *97*, 10269–10280.

[24] J. Wang, W. Wang, P. A. Kollman, D. A. Case, *J. Mol. Graphics Modell.* **2006**, *25*, 247–260.

[25] A. W. S. da Silva, W. F. Vranken, *BMC Res. Notes* **2012**, *5*, 367.

[26] C. Creze, B. Rinaldi, R. Haser, P. Bouvet, P. Gouet, *Acta Crystallogr. D Biol. Crystallogr.* **2007**, *63*, 682–688.

[27] E. F. Pettersen, T. D. Goddard, C. C. Huang, G. S. Couch, D. M. Greenblatt, E. C. Meng, T. E. Ferrin, *J. Comput. Chem.* **2004**, *25*, 1605–1612.

[28] C. J. Lech, B. Heddi, A. T. Phan, *Nucleic Acids Res.* **2013**, *41*, 2034–2046.

[29] F. Rosu, V. Gabelica, N. Smargiasso, G. Mazzucchelli, K. Shin-Ya, E. D. Pauw, *J. Nucleic Acids* **2010**, *2010*, 121259.

[30] G. N. Parkinson, F. Cuenca, S. Neidle, *J. Mol. Biol.* **2008**, *381*, 1145–1156.

[31] U. Essmann, L. Perera, M. L. Berkowitz, T. Darden, H. Lee, L. G. Pedersen, *J. Chem. Phys.* **1995**, *103*, 8577–8593.
